# Ovarian gene expression in the absence of FIGLA, an oocyte-specific transcription factor

**DOI:** 10.1186/1471-213X-7-67

**Published:** 2007-06-13

**Authors:** Saurabh Joshi, Holly Davies, Lauren Porter Sims, Shawn E Levy, Jurrien Dean

**Affiliations:** 1 Laboratory of Cellular and Developmental Biology, NIDDK, National Institutes of Health, Bethesda, MD 20892, USA; 2 Department of Biomedical Informatics, Vanderbilt University Medical Center, Nashville, TN 37232, USA

## Abstract

**Background:**

Ovarian folliculogenesis in mammals is a complex process involving interactions between germ and somatic cells. Carefully orchestrated expression of transcription factors, cell adhesion molecules and growth factors are required for success. We have identified a germ-cell specific, basic helix-loop-helix transcription factor, FIGLA (**F**actor **I**n the **G**erm**L**ine, **A**lpha) and demonstrated its involvement in two independent developmental processes: formation of the primordial follicle and coordinate expression of zona pellucida genes.

**Results:**

Taking advantage of *Figla *null mouse lines, we have used a combined approach of microarray and **S**erial **A**nalysis of **G**ene **E**xpression (SAGE) to identify potential downstream target genes. Using high stringent cutoffs, we find that FIGLA functions as a key regulatory molecule in coordinating expression of the NALP family of genes, genes of known oocyte-specific expression and a set of functionally un-annotated genes. FIGLA also inhibits expression of male germ cell specific genes that might otherwise disrupt normal oogenesis.

**Conclusion:**

These data implicate FIGLA as a central regulator of oocyte-specific genes that play roles in folliculogenesis, fertilization and early development.

## Background

Primordial germ cells migrate to and colonize the mouse gonad, completing the process during embryonic day 10.5 (E10.5) to E12.5 [1]. Subsequent phenotypic sexual dimorphism is defined by the gonad and mice lacking *Sry *located on the Y chromosome follow a constitutive female pathway [2], presumably instructed by a unique set of genes [3]. Entrance of female germ cells into meiosis at E13.5 is a defining event mediated by retinoid responsive genes [4,5]. A second major transition occurs perinatally when flattened granulosa cells surround individual germ cells to form primordial follicles [6], forming a reservoir of eggs available for subsequent fertilization [7]. Germ cells that fail to interact with the gonadal somatic cells, either by ectopic location [8] or in the absence of a critical oocyte-specific transcription factor, FIGLA (previously known as FIGα)[9], do not survive.

FIGLA (**F**actor **I**n the **G**erm**L**ine, **A**lpha), a basic helix-loop-helix transcription factor, was first defined in the coordinate regulation of three genes (*Zp1*, *Zp2*, *Zp3*) encoding proteins that form the zona pellucida surrounding ovulated eggs [10]. FIGLA transcripts are detected as early as E13.5 in the embryonic gonad and persist in the adult ovary [9]. FIGLA protein, however, is not detected until E19 based on sensitive gel mobility shift assays [11]. Mice lacking FIGLA have normal embryonic gonad development, but primordial follicles do not form at birth and germ cells are lost within days. Female, but not male, mice are sterile [9]. These data suggested that FIGLA plays critical roles in female germline and follicle development, but the full complement of downstream gene targets involved in these processes and when in development they become activated have not been defined

Using cDNA microarrays, we have compared the transcriptomes of normal and *Figla *null ovaries at four developmental time points (E12.5 to newborn). These results have been complemented with SAGE (**S**erial **A**nalysis of **G**ene **E**xpression) libraries derived from newborn ovaries to identify potential direct and indirect gene targets of FIGLA in female gonad development.

## Results

### Microarray data analysis

To identify potential downstream gene targets of FIGLA, total RNA was obtained from normal and *Figla *null gonads isolated from E12.5, E14.5, E17.5 and newborn female mice. Three independent biological samples obtained from each embryonic time point and four from newborn gonads were linearly amplified, labeled with Cy3 and Cy5 and hybridized to the National Institute of Aging (NIA) cDNA microarray consists of ~22K features enriched for transcripts from newborn ovaries, pre- and peri-implantation embryos [12,13]. After washing, the average statistically significant intensities for each element were analyzed with Gene Spring GX software. Features that varied more than 2-fold (with a coefficient of variance less than 30%) between normal and *Figla *null gonads were selected for further analysis.

**Figure 1:**
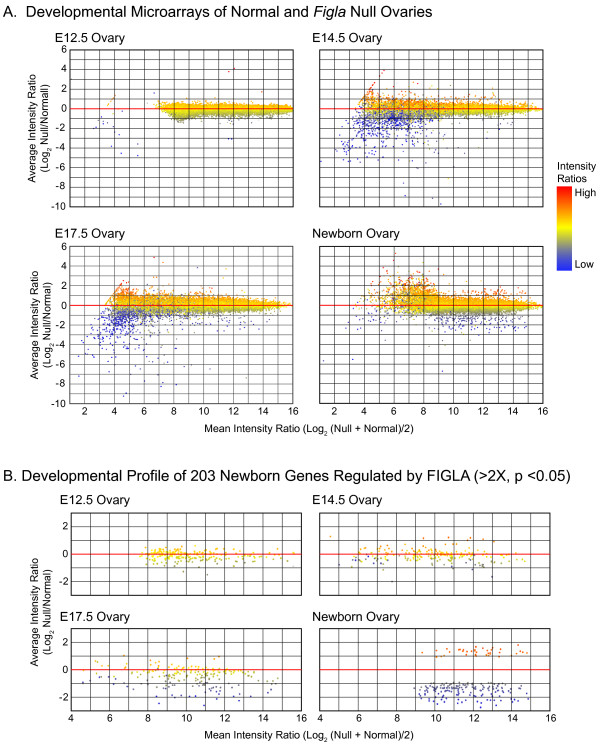
**Differential gene expression in normal and *Figla *null ovaries**. A. Embryonic ovarian transcriptomes of normal and *Figla *null mice at embryonic days E12.5, E14.5, E17.5 and newborn. For elements in the NIA microarray, the mean intensity ratio log_2 _null (red) over normal (blue) on X axis is plotted against the average intensity ratio log_2 _null and normal on Y axis. Data represent mean of 3–4 independent biological samples with Cy3 and Cy5 dye reversals and spiked Ambion RNA controls. B. Same as (A) except restricted to expression profiles of 203 newborn genes regulated by FIGLA (≥ 2 fold, ρ ≤ 0.05, after analysis of variance).

The M (mean log ratio) vs. A (average log_2 _signal value) scatter plots reflect the fold change of differentially expressed genes in *Figla *null and normal ovaries on the Y axis relative to their abundance on the X axis (Fig. 1). Thus, transcripts with low intensity ratios (blue) indicate genes that are potentially up-regulated by FIGLA and transcripts with high intensity ratios (red) represent genes that are potentially down-regulated by FIGLA. As expected, no genes were differentially expressed at E12.5, a point in development prior to the onset of *Figla *expression. Only a few differences were observed at E14.5 and E17.5 with 6 and 4 genes up-regulated ≥ 2-fold (ρ ≥ 0.05) in normal ovaries and 8 and 1 up-regulated in *Figla *null ovaries, respectively (Fig. 1A). In marked contrast, 176 transcripts were ≥ 2-fold more abundant in normal and 44 were ≥ 2-fold more abundant in *Figla *null newborn ovaries (ρ ≤ 0.05).

### Developmental hierarchical cluster analysis of FIGLA regulated genes

**Figure 2:**
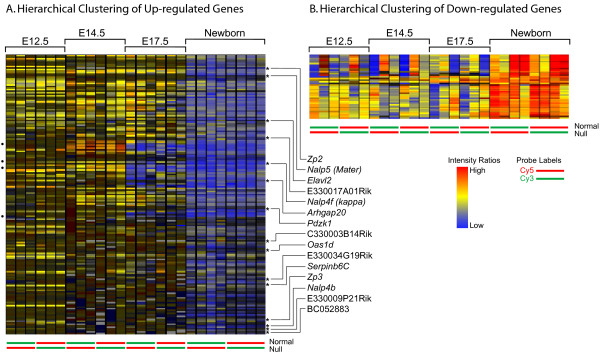
**Hierarchical clustering of transcripts**. Developmental hierarchical clustering of newborn transcripts potentially up- (A) or down-regulated (B) by FIGLA. Individual genes are represented by horizontal bar. Each lane represents an independently obtained biological sample (three for E12.5, E14.5 and E17.5; four for newborn) with Cy3 and Cy5 dye reversals indicated at the bottom. Blue represents greater abundance in the presence of FIGLA and red indicates less. Four genes [NIA:551381, NIA:H330A03, NIA:H3134D03, NIA:H3058H02] up-regulated at E17.5 and newborn are indicated with dots to the left. Genes encoding transcripts characterized in greater detail are indicated by an asterisk and are labeled to the right. Both *Zp2 *and *Zp3 *were identified by the screen, but not characterized further.

The average intensities of hybridization of the 176 genes that were less abundant (i.e. potentially up-regulated, Fig. 2A) and the 44 genes that were more abundant (i.e., potentially down-regulated, Fig. 2B) in *Figla *null newborn ovaries were compared by hierarchical cluster analysis. Almost all the 176 potentially up-regulated genes showed similar expression pattern wherein the expression of these genes commenced only at newborn stage of the ovary development. As expected, both *Zp2 *and *Zp3*, previously described direct downstream targets of FIGLA, were up-regulated in the normal newborn ovaries, although they were not closely clustered to each other. The four genes [NIA:551381, H330A03, H3134D03, H3058H02] that were first up-regulated E17.5 and persisted in the newborn also did not cluster together (dots, Fig. 2A). The corresponding positions of the genes which were further characterized by qRT-PCR and in situ hybridizations are marked by asterisks and labeled. The 44 genes which were potentially down regulated by FIGLA (i.e., more abundant in *Figla *null ovaries) all had similar expression patterns with the major change in expression occurring in the newborn ovary (Fig. 2B).

Of these, 165 of the potentially up-regulated and 38 of the potentially down-regulated transcripts were judged to differ with statistical significance after analysis of variance (ANOVA). There was no overlap of regulated genes (≥ 2-fold, ρ ≤ 0.05) among the various time points except for 2 genes [NIA:551381, NIA:H330A03] up-regulated at E17.5 that were also up-regulated in the newborn ovary (Fig. 1B). Genes that were potentially up- and down-regulated are provided (see Additional files 1 and 2). Using gene ontology software PANTHER [14], the 203 genes that were differentially-regulated in the newborn were analyzed based on their molecular function (Table 1). Of the 165 up-regulated genes, 25% were grouped in unknown molecular function category, 13% were nucleic acid binding proteins, 7% were transcription factors and 6% were genes with transferase activity. Of the 38 down-regulated genes, 23% were transcription factors, and 20% encoded proteins with nucleic acid binding functions. Two of these genes, *Taf7l *and *Tia1*, are normally expressed in the testes. Genes with unknown molecular function and transferase activity were comparable (25% and 7%, respectively) to the up-regulated genes.

**Table 1:** Differentially regulated genes

**Gene ontology**	**Up-regulated genes**		**Down-regulated genes**	
	
	**Microarray**	**SAGE**	**Microarray**	**SAGE**
Nucleic acid binding proteins and transcription factors	*Pou5f1, E2f5, Stat3, Lcp1, Zfp313, Og2X, Rex, Helic1, Cpeb3, Elav2, Cpeb1, Dhx40Top3b, Ccrn41*, E430034L04^b^, 5830484A20	*Pou5f1, Sp3, Dtx2, Peg3, Tcf21, Eef1a1, Rps2, Hdac2*	*Phtf1*, ***Taf7l***^a^*, Mrg1, Ncam2*, ***Tia1***, 1110031M08	*Mxd3, Pbx3, MkI1, Sox17, Jarid1c, Sp8, Mjd, Mkrn1, FhI4, Sdpr, Miz1, BC029103, Papolb, Rnaseh2a, Sox17, Tdrd6, Sf3a1, 5*, ***Tnp2****, Mgmt*, ***Hils1***, AV34037
Receptors	*Kit, Grid2, Drd3, Trpm7*	*Lum, Obrgrp*		*Gabra2, Pvrl3, Tcp10b, Plxnb3, Mass1*
Extracellular matrix proteins	*Zp2, Zp3, Col9a3*	*Zp2, Leprel2, Col1a1, Mfap2*,		
Cell adhesion molecules	*Pcdhga12*			
Chaperones	*Grpel2, Tor3a*	*Hspa8, Vbp1*		***Clgn****Dnajb1, CPN60, Osp94, Dnajb3, Hsbp9*
Cytoskeletonal proteins	*Kif14, Rdx, Lmo7, Lcp1*, AI427122, 2900002G04	*Flna*		*Kif2b, Dncl2b, Actl7a, Tuba3*, ***Tekt1***, ***Fscn3****, Tuba5, Rsn*, ***Dnahc8****, Tns*
Defense and immunity				*And, H2-Bf, Pvrl3*
Hydrolases	*Thedc1, Helic1, Exo1*, *D7Ertd445e*, 2610020H15			*Rnaseh2a, Ephx2, Asrgl1, Dnahc8*, *Dpysl3*, AV340375,
Oxido-reductases	*Aof1, Aass*	*Paox, Prdx1, Akr1a4*		*Ldhal6b, Sdh1*, ***Ldhc****, Cyp11a1, Txndc2, Gpd2, Sqrdl, prdx6-rs1, Cyp17a1, Akr1c19, Mcsp*, AL024210
Synthases	*Oas1c, Slc27a2*	*Oas1h*		
Isomerases	*Top3b*			*Pgam2, Ppil1*
Kinases	*Rock1, Ak4*			*Stk33, Dyrk3, Plxnb3, Cdc2l6*,
Ligases	*Herc3, Slc27a2, Rnf35*, 2610020H15			*Herc4, Fbox36, Rnf133, Acsl1, Miz1, Glul, Rnf139,, Senp7*, 630025C22
Proteases	*Ctrc, Prep*			*And, Tpp2, Ctsd, 3, Asrgl1, Klk6*, ***Adam3***, 1700074P1
Transferases	*Chst10, Fdft1, Bcat1, Nmna3, Ipmk*, Ak44930403J22, 4930487N19	*Ogt, Siat9*	*Ilvbl, Got2*	*Tcp10b, Sia5, B4galt1, Mgmt, Sas*
Cell junction proteins	*Pard3*			
Carrier proteins	*Osbpl8*	*Scamp5*		
Membrane traffic proteins	*Beta-NAP*	*Stxbp1, Clta*		*Stx6*
Ion channels	*Trpm7, Grid2*	*Prdx1*		*Gabra2, Kcne3, Kcnk4*
Select regulatory molecules	*Chn1, Argap20, Top3b, Serpinb6C*	*Rraga*		***Oaz3***, ***Akap3****, Ppp1r3a, Dusp18*, 4933414G08

^a^Testis-associated genes are in bold

^b^Genbank accession numbers

### Ovarian genes affected by *Figla *expression

**Figure 3:**
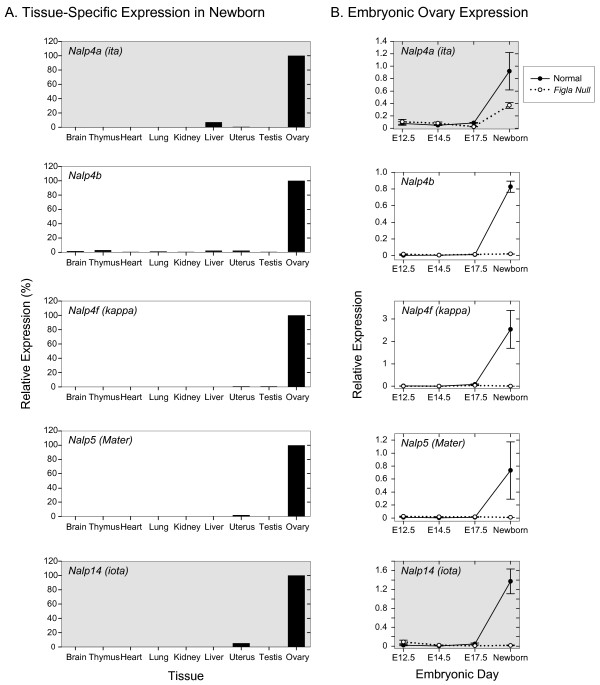
**Expression of NALP genes**. Tissue-specific and developmental expression of NALP genes potentially up-regulated by FIGLA. A. qRT-PCR of total RNA isolated from somatic and reproductive tract tissues normalized to HPRT and plotted relative to 100% expression in ovary. B. qRT-PCR of total ovarian RNA isolated from normal and *Figla *null mice at E12.5, E14.5, E17.5 and newborn and plotted relative to HPRT. Data is the average of two independently isolated biological samples, analyzed in triplicate at each developmental time point. *Nalp4a *(ita) and *Nalp14 *(iota)(shaded) were previously identified as ovary-specific and were not represented in microarray or SAGE data.

**Figure 4:**
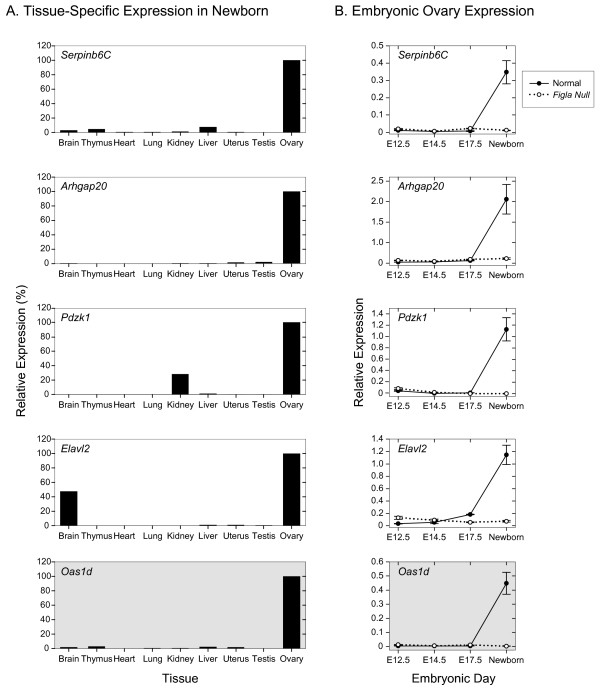
**Expression of known genes**. Tissue-specific and developmental expression of five known genes potentially up-regulated by FIGLA. A. qRT-PCR of total RNA isolated from somatic and reproductive tract tissues normalized to HPRT and plotted relative to 100% expression in ovary. B. qRT-PCR of total ovarian RNA isolated from normal and *Figla *null mice at E12.5, E14.5, E17.5 and newborn and plotted relative to HPRT. Data is the average (± s.e.m.) of two independently isolated biological samples, analyzed in triplicate at each developmental time point. *Oas1d *(shaded) missed the statistical cutoff because of a single (out of eight) outlying datum point.

**Figure 5:**
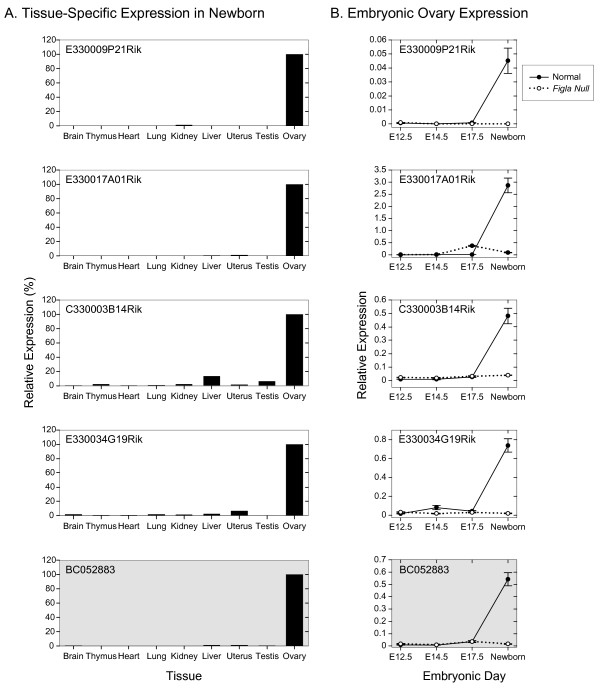
**Expression of un-annotated genes**. Tissue-specific and developmental expression of five functionally un-annotated genes potentially up-regulated by FIGLA. A. qRT-PCR of total RNA isolated from somatic and reproductive tract tissues normalized to HPRT and plotted relative to 100% expression in ovary. B. qRT-PCR of total ovarian RNA isolated from normal and *Figla *null mice at E12.5, E14.5, E17.5 and newborn and plotted relative to HPRT. Data is the average (± s.e.m.) of two independently isolated biological samples, analyzed in triplicate at each developmental time point. [Genbank:BC052883] (shaded) missed the statistical cutoff because of a single (out of eight) outlying datum point.

Thirteen genes that were ≥ 2-fold more highly expressed in normal than *Figla *null newborn ovaries were chosen for more detailed analysis. Three were members of the *Nalp *gene family that have oocyte-specific expression [15]; five were functionally annotated genes with oocyte-specific expression; and five were functionally un-annotated. Two additional members of the *Nalp *family (*Nalp4a, Nalp14*) that were up-regulated by FIGLA, albeit to a lesser extent, were included in the analysis and two of the selected genes (*Oas1d*, [Genbank:BC052883]) missed the statistical cutoff because of single (out of eight) outlying data points (shaded backgrounds in Figs 3, 4, 5). Primers specific for each gene were designed (see Additional file 3) and the presence and absence of specific transcripts in newborn normal and *Figla *null ovaries were confirmed in 14 of the 15 by qRT-PCR (all but *Nalp4a*).

Total RNA was isolated from 9 normal newborn organs and assayed for gene expression which was normalized to HPRT and standardized to 100% in the ovary (Fig. 3A). As previously reported, all five *Nalp *genes were expressed in the ovary with low levels of transcripts (< 5% of ovarian expression) observed in the uterus for *Nalp14 *and in the liver for *Nalp4a *of newborn mice. The developmental profiles of transcript abundance from E12.5 to newborn were consistent with FIGLA regulating expression of *Nalp4b*, *Nalp5*, *Nalp4f *and *Nalp14*. No expression was detected in E12.5, E14.5, E17.5 or newborn gonads isolated from *Figla *null mice, but all five genes were expressed in normal newborn ovaries. However, the absence of FIGLA did not preclude expression of *Nalp4a *in newborn ovaries, although expression was ~60% of normal. Although the members of the *Nalp *gene family share structural motifs and are co-expressed in oocytes, they are not functionally redundant and the inactivation of one (*Nalp5*) is sufficient to arrest pre-implantation development [16].

Among the other annotated genes, each was preferentially expressed in the ovary with only low levels (< 5% of ovarian expression) of *Oas1d*, *Serpinb6C *and *Arhgap20 *transcripts in other tissues in newborn mice (Fig. 4A). Expression of *Pdzk1 *and *Elavl2 *was less tightly controlled with transcripts detected in kidney (~25% of ovarian expression) and brain (~40%), respectively. Each of these five genes was first detected in the newborn ovary where their expression was dependent on FIGLA (Fig. 4B). These observations are consistent with each gene being a direct downstream target of FIGLA, but transcripts of each are detected in adult tissues, suggesting that other transcription factors play a role in developmental and organ specific expression. *Oas1d *encodes an oocyte-specific dsRNA, 2'5'-oligoadenylate synthetase that is ~60% identical to OAS1A that has been implicated in the interferon defense response against viral infections. Genetically altered mice, lacking OAS1D, are fertile, but have reduced fecundity associated with lowered ovulatory capacity and defects in early embryonic development [17]. Mice lacking PDZK1, implicated in controlling ion transport and cholesterol metabolism [18], had normal fertility and produced off-spring with the expected Mendelian inheritance of the *Pdzk1 *null allele [19].

### Tissue and developmental expression of unannotated genes

Five additional genes that were more abundant in normal than in *Figla *null newborn ovaries and functionally un-annotated were also selected for further evaluation (Fig. 5). Three [E330009P21Rik, E3300017A01Rik, Genbank:BC052883] were detected only in the ovary by qRT-PCR and the remaining two [C330003B14Rik, E330034G19Rik] had only low levels of expression in other tissues. All five genes had similar developmental profiles as the aforementioned genes with little expression prior to birth and virtual absence of expression in *Figla *null ovaries.

**Figure 6:**
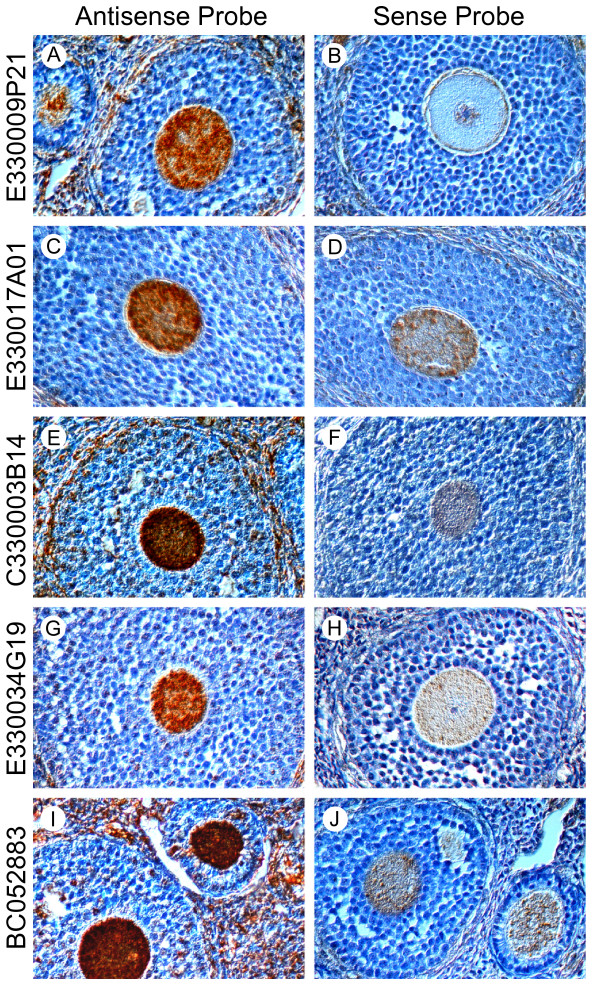
**In situ hybridization**. In situ hybridization of the five functionally un-annotated genes potentially up-regulated by FIGLA. Paraformaldehyde fixed and paraffin embedded adult ovarian section were hybridized with digoxigenin (DIG) labeled antisense (A, C, E, G, I) or sense (B, D, F, H, J) synthetic oligonuclotides probes specific to [E330009P21Rik] (A, B), [E330017A01Rik] (C, D), [C330003B14Rik] (E, F), [E330034G19Rik] (G, H) and [Genbank:BC052883] (I, J) cDNAs.

In situ hybridizations were performed using the digoxigenin-labeled oligonucleotide probes designed specifically for transcripts of each gene (Additional file 4). All the functionally unknown genes showed predominant expression in oocytes (Fig. 6) with only background staining surrounding ovarian tissue. Although some binding was observed with sense probes for [E330017A01Rik, C330003B14Rik and Genbank:BC052883](Fig. 6D, F, J), it was minor compared to the strong signals observed with anti-sense probes (Fig. 6C, E, F).

### SAGE analysis of normal and *Figla *null newborn ovaries

**Figure 7:**
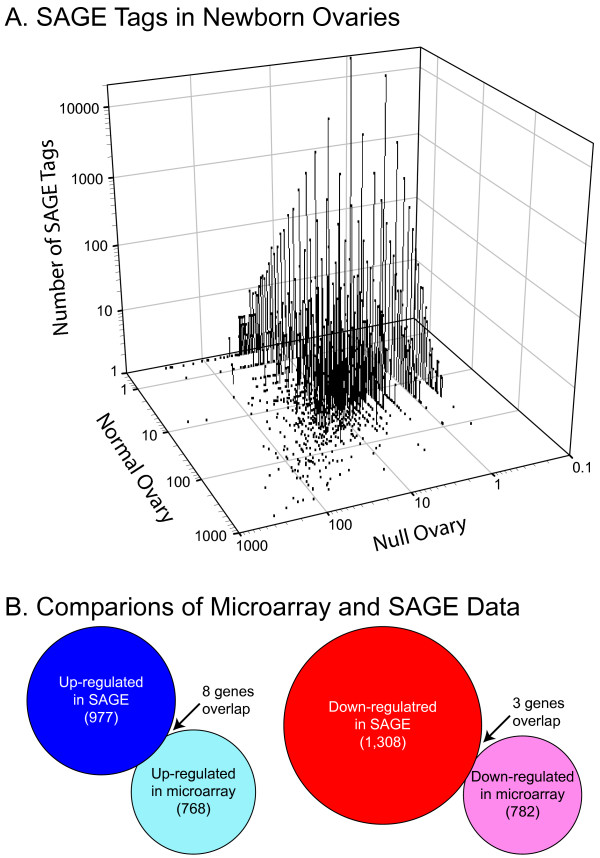
**Serial analysis of gene expression**. Serial analysis of gene expression (SAGE) of newborn ovaries of normal and *Figla *null mice. A. Three dimensional plot of the number of SAGE tags (10 bp) in normal and *Figla *null newborn ovaries. B. Comparison (ρ ≤ 0.05) of transcripts that are up-regulated (blue) or down- regulated (red) by FIGLA in SAGE analysis [20] and by microarray using an FDR threshold of 5% [37]. Size of circles reflects the number of transcripts. Eleven transcripts were detected in both platforms, eight of which were up-regulated (POU5F1, ZP2, IVNS1ABP, VBP1, PADI6, RBPMS2, Genbank:EG626058, 6430591E23Rik) and three of which were down-regulated (SP3, HDAC2, OGT) in newborn normal ovaries.

The developmental microarray analysis suggested that significant differences in potential FIGLA gene targets were not observed prior to birth. Therefore, to complement these data and avoid the bias introduced by pre-selection of elements on the microarrays, a SAGE analysis was undertaken to provide identity and relative abundance of individual transcripts. Total RNA was isolated from newborn ovaries and used to construct a normal and a *Figla *null SAGE library in which transcripts were identified by a 10 nt tag and their relative abundance by the number of tags detected. The 79,095 quality tags sequenced from the normal library and the 77,851 from the null library represented 23,595 and 32,123 different transcripts, respectively. 15,838 tags were unique to the normal library; 24,366 were unique to the null library and 7,757 were present in both. To avoid sequence-error artifacts, only tags that were observed at least twice in the normal (7,715) and *Figla *null (9,477) SAGE libraries were included in subsequent analyses (Fig. 7A).

Using the Audic and Claverie algorithm that incorporates Bayesian and false alarm analyses [20], 977 genes more highly expressed in the normal library and 1308 genes more highly expressed in the *Figla *null library were identified (Fig. 7B). The data also were analyzed with other statistical tests commonly used for SAGE [21] and Fisher's exact test provided similar results (> 90% overlap) with 810 genes more highly expressed in normal and 1318 more highly expressed in null ovaries. However, only *Pou5f1 *(*Oct4*) and *Zp2 *were observed as up-regulated in both newborn microarrays and SAGE libraries and no commonly down regulated genes were observed among those identified by SAGE and by microarray analyses. Although the microarray and SAGE screens were selected to complement one another in identifying potential downstream targets of FIGLA, the lack of overlap in targets was unanticipated. The data from the newborn normal and *Figla *null microarray data were reexamined by False Discovery Analysis [22]. Of 2,233 identified features (FDR threshold of 5%) from the microarray data, 1,550 had single unigene annotations [23], 202 mapped to multiple clusters and 248 were not mapped. Of the annotated genes, 768 were up-regulated (see Additional file 5) and 782 were down-regulated (see Additional file 6). Eleven genes (marked in Additional files 5, 6), including *Pou5fl *and *Zp2 *from our initial analysis, were also present in our SAGE analysis. Eight were up-regulated *(Pou5f1*, *Zp2*, *Ivns1abp*, *Vbp1*, *Padi6*, *Rbpms2*, Genbank:EG626058, 6430591E23Rik) and three were down-regulated (*Sp3*, *Hdac2*, *Ogt*) in newborn normal ovaries. Because alternative statistical analyses [24-26] may be useful for the comparison of these data sets, we have made the original microarray and SAGE data available at Gene Expression Omnibus [27] under accession GSE5558 and GSE5802, respectively.

Of the genes identified in the SAGE screen, 64 (see Additional file 7) of the potentially up-regulated genes had ≥ 10 tags in normal and none in the null and 312 (see Additional file 8) of the potentially down-regulated genes had ≥ 10 tags in the null and none in normal ovaries. These 376 tags were reliably mapped to genes using the NCBI SAGE database [28] and ontology of 112 was determined by PANTHER (Table 1). The greatest percentage (26%) of genes were nucleic-acid binding proteins (including transcription factors) followed by oxidoreductases (13%), cytoskeletonal proteins (10%) and ligases (8%). Ten of the down-regulated genes (*Tnp2*, *Hils1*, *Clgn [Calmegin]*, *Tekt1*, *Fscn3*, *Dnahc8*, *Ldh3*, *Adam3 [Cyritestin]*, *Oaz3*, *Akap3*), scattered among the categories, have sperm-associated patterns of expression (Table 1).

### Expression of eight up-regulated genes from the SAGE analysis

**Figure 8:**
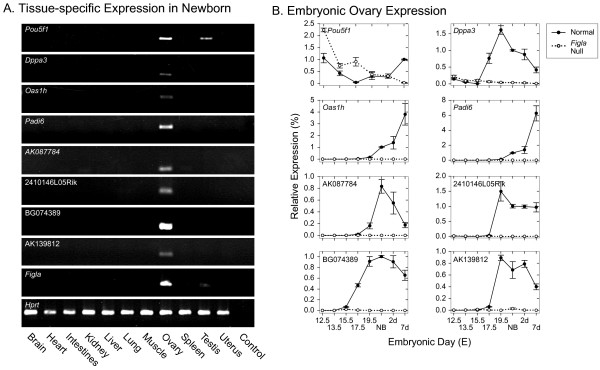
**Expression of FIGLA targets**. Tissue-specific and developmental expression of eight genes potentially up-regulated by FIGLA in SAGE analysis. SAGE tags for each gene were absent in the *Figla *null newborn library and present ≥ 10 in the normal newborn library. A. Semi-quantitative RT-PCR of total RNA isolated from somatic and reproductive tract tissues with primers specific for each of the eight genes. B. qRT-PCR of total ovarian RNA isolated from normal and *Figla *null mice at E12.5, E13.5, E15.5, E17.5, E19.5, newborn (NB), 2dpp and 7dpp and plotted relative to HPRT. Data is the average (± s.e.m.) of 2–6 independently isolated biological samples, analyzed in triplicate at each developmental time point.

Eight transcripts for which there were ≥ 10 tags in the normal and 0 tags in the *Figla *null newborn SAGE libraries (*Pou5f1*, *Dppa3*, *Oas1h*, *Padi6*, [Genbank:AK087784, 2410146L05Rik, Genbank:BG074389, Genbank:AK139812]) were analyzed further. Transcripts for each were detected by semi-quantitative RT-PCR in normal, but not *Figla *null newborn ovaries. Total RNA from 11 tissues was analyzed and transcripts for each gene were detected only in ovarian tissue, with the exception of *Pou5f1*, which was also present in testis (Fig. 8A).

*Pou5f1 *(*Oct4*) is expressed in pluripotent cells during mouse development before becoming restricted to germ cells [29] and regulates a significant number of downstream target genes by itself and in tandem with other transcription factors [30]. The complex pattern of POU5F1 transcripts in normal and *Figla *null mice (Fig 8B) indicates participation of other transcription factors in controlling its expression. Expression of the other seven genes was not detected in *Figla *null mice (except for a very modest accumulation of [Genbank:BG084789] at E15.5) and was first observed in normal ovaries at E15.5 (*Dppa3*, [Genbank:AK139812] or E19.5 (*Oash1*, *Padi6*, [Genbank:AK087784, 2410146L05Rik, Genbank:AK139812])(Fig. 8B).

*Dppa3 *(*Stella*), originally implicated in germ cell lineage specification [31], is a maternal effect gene required for pre-implantation development [32,33]. *Oas1h *encodes a 2'5'-oligoadenylate synthetase and clusters with *Oas1d *(detected by microarray analysis) along with 6 other closely related synthetases on mouse Chr5:119,938,130-120,073,460 [34]. *Padi6 *(*Padi5*) is one of 4 similar genes (*Padi1–4*) clustered on mouse Chr4:139,787,689-139,623,878 that encodes a peptidylarginine deiminase which converts arginine residues into citrulline [35]. It is expressed during oogenesis where it is associate with cytoplasmic sheets and the protein persists in the early embryo up to the blastocyst stage [36].

Each of the remaining four SAGE tags matched either cDNA or spliced EST that was expressed in ovarian tissue and/or the early mouse embryo. [Genbank:AK087784] is a full-length cDNA from a 2 days pregnant adult female ovary [E330020D12Rik] that maps to mouse Chr1:153,290,975-153,292,514 and [2410146L05Rik] is a female germline specific cDNA that encodes a hypothetical 166 amino acid protein mapping to mouse Chr9:78,577,285-78,578,468. [Genbank:BG074389], a 532 bp spliced EST (Chr11:7,077,950-7,079,635), is part of the NIA15K cDNA clone set and was up-regulated in the microarray screen, albeit not to a statistically significant extent. [Genbank:AK139812] was reliably matched by a SAGE tag that also matched two other sequences, neither of which was ovary specific. [Genbank:AK139812] corresponds to a full-length cDNA [B020018122Rik] obtained from a 2-cell embryonic library and maps as a spliced EST on mouse Chr12:107,907,672-107,914,726.

## Discussion

During ovarian development, oocytes accumulate maternal products necessary for successful folliculogenesis, fertilization and pre-implantation development. FIGLA is an oocyte-specific, basic helix-loop-helix transcription factor required for perinatal formation of primordial follicles and the establishment of the extracellular zona pellucida matrix that surrounds eggs to mediate fertilization and an embryonic block to polyspermy. Transcripts encoding FIGLA are first detected at E13.5 in female gonads and persist in adult ovaries [9,10]. The involvement of FIGLA in two, independent, oocyte-specific genetic pathways and its persistence during critical periods of ovarian development suggest that FIGLA may regulate other genes critical for successful gonadogenesis and development. Two complementary approaches using spotted-glass microarrays and **S**erial **A**nalysis of **G**ene **E**xpression (SAGE) have been combined to identify additional potential downstream targets of FIGLA.

Taking advantage of *Figla *null mice and the NIA ~22K microarray that contains elements enriched for expression in oocyte and early development [12,13], ovarian gene expression was profiled at four embryonic time points from E12.5 to newborn. No statistically significant differences (≥ 2X, ρ ≤ 0.05) in transcript abundance between normal and *Figla *null mice was observed at E12.5 and E14.5 and only two transcripts differed at E17.5. However, in newborn ovaries, 165 genes were up-regulated (i.e., less abundant in *Figla *null) and 38 were down-regulated (i.e., more abundant in *Figla *null). This developmental time point is just after FIGLA protein is detected in a sensitive gel mobility shift assay [11] and coincident with the first phenotypic manifestation of *Figla *null mice [9]. Thus, although *Figla *transcripts are detected as early as E13.5, the major affect on ovarian gene expression occurs perinatally.

Microarrays are limited by the elements spotted on glass during their fabrication. Therefore, to broaden the search for potential direct or indirect downstream gene targets, SAGE libraries were constructed from poly(A)^+ ^RNA isolated from newborn normal and *Figla *null ovaries. A 10 base tag immediately adjacent to the 3' most Sau3A1 restriction enzyme cleavage site was used to identify 7,715 transcripts in normal and 9,455 transcripts in *Figla *null mice. Of the genes encoding these transcripts, 977 were up-regulated and 1,308 were down-regulated when analyzed with statistics that incorporate Bayesian and false alarm analyses [20]. Unigene designations were available for 838 of the up-regulated and 648 of the down-regulated genes and 31% (334 up-regulated; 131 down-regulated) were represented among the elements on the NIA microarray. Initially only two (*Pou5f1 *and *Zp2*) were identified as up-regulated on both platforms. However, reanalysis of the newborn microarray data with an FDR threshold of 5% [37] identified 11 genes in common, eight of which were up-regulated (*Pou5f1*, *Zp2*, *Ivns1abp*, *Vbp1*, *Padi6*, *Rbpms2*, Genbank:EG626058, 6430591E23Rik) and three of which were down-regulated (*Sp3*, *Hdac2*, *Ogt*) in newborn normal ovaries. A similar lack of concordance among different platforms analyzing the same biological samples, particularly for genes with low abundance, has been observed previously [38-41].

'Guilt by association' has been invoked to identify genetic hierarchies, members of which are co-regulated by specific transcription factors (for review, see [42]). In examining the ontology of the differentially regulated genes detected by microarray and SAGE analysis, nucleic acid binding proteins and transcription factors formed the largest group. One gene, *Pou5f1 *(*Oct4*) in this grouping was up-regulated by FIGLA on both platforms and is initially present in primordial oogonia prior to down-regulation as female germ cells enter the prophase of meiosis I (~13.5E). Perinatally, *Pou5f1 *transcripts and POU5F1 protein once again become abundant in growing oocytes [43] and persist in the early embryo prior to zygotic expression which begins in 4-cell embryos. Although initially present in all blastomeres, *Pou5f1 *transcripts becomes restricted to the inner cell mass, the epiblast and finally primordial germ cells by E8.5 [44]. The expression of *Pou5f1 *is regulated by a TATA-less promoter with distal and proximal enhancers implicated in regulating transcript levels during embryogenesis [45]. However, the molecular control of *Pou5f1 *expression during gametogenesis has not been determined. A canonical E-box (CANNTG) is present -261 bp upstream of the *Pou5f1 *transcription start site and represents a well-defined binding sites for basic helix-loop-helix transcription factors [46]. FIGLA protein is first detected at E19 [11] just prior to the post-natal up-regulation of *Pou5f1 *[43] and in both microarray and SAGE analyses, *Pou5f1 *expression is down-regulated in *Figla *null mice. These data are consistent with FIGLA acting as a regulator of *Pou5f1 *expression in the female germline, although other factors must play a role as well.

Structural proteins, including those involved with extracellular matrices and intracellular cytoskeleton were also well represented in the gene ontology analysis and one, *Zp2*, was up-regulated by FIGLA on both platforms (microarray and SAGE). *Zp2 *encodes a major component of the mouse zona pellucida that surrounds growing oocytes, ovulated eggs and pre-implantation embryos [47]. Mice lacking ZP2 initially form a thin zona matrix composed of ZP1 and ZP3 that does not persist and no zona pellucida is observed in ovulated eggs which renders female mice sterile [48]. FIGLA was initially defined as transcription activating activity that bound to a conserved E-box, within the first 200 bp of *Zp1*, *Zp2 *and *Zp3 *promoters and was subsequently isolated by expression cloning [10,49]. ZP2 transcripts can be detected as early as E19, although the zona matrix does not form until oocytes begin to grow after birth; no ZP2 transcripts are detected in *Figla *null mice [9]. Thus, *Zp2 *expression appears to reflect direct targeting of FIGLA during the onset of oogenesis.

All twenty-three of the genes selected from the microarray and SAGE screens were preferentially expressed in newborn ovarian tissue, although significant levels of *Pdzk1 *and *Elavl2 *transcripts were detected in kidney and brain, respectively. There was considerable variation in levels of expression in normal newborn ovaries, ranging from 14–400% of HPRT levels which may indicate the importance of additional co-factors in regulating gene expression, although differing efficiencies of PCR amplification may affect these comparisons. All genes (except *Nalp4a*) were virtually absent in newborn *Figla *null ovaries. Those genes expressed uniquely in oocytes, may be of particular importance in ovarian gonadogenesis.

Although well appreciated in simple model organisms, only in recent years have maternal effect genes have become well documented in mice. The protein products of these genes accumulate during oocyte growth and are required for successful embryogenesis. While it remains controversial whether such maternal products play a role in establishing embryonic polarity [50,51], there is increasingly ample molecular evidence that maternal effect genes are critical in pre- [16,32,33,52-59] and post-implantation [57,60-63] development. *Mater *(***M****aternal ***a***ntigen ****T****hat ****E****mbryos ****R****equire*) was one of the earliest maternal effect genes molecularly characterized in mice. Mice lacking this 125 kDa cytoplasmic protein have normal gonadogenesis and ovulate eggs that can be fertilized, but do not progress beyond the two-cell stage [16]. *Mater *(*Nalp5*) is a member of the Nalp family and genetic studies are underway to determine if other members regulated by FIGLA (*Nalp4b*, *Nalp4f*, *Nalp14*) affect pre-implantation in mice. A second maternal effect gene *Dppa3 *(*Stella*), also required for pre-implantation development [32,33], was detected by the SAGE analysis and there may be others as well.

Of the 350 genes that were significantly less abundant in normal ovaries (see Additional files 2 and 6), 30 were testis-associated and 12 were classified by the Panther gene ontology software (Table 1). Two were transcription factors, preferentially expressed in the testis, *Taf7l *and *Phtf1*. TAF7L is a TATA box binding protein involved in differentiation of spermatogonia to spermatocytes [64] and PHTF1 is a putative homeodomain transcription factor with male germ cell specific expression that binds feminizing factor FEM1B [65,66]. Another two, *Tnp2 *and *Hils1*, encode transition protein 2 and a spermatid-specific linker histone H1-like proteins, respectively, which are involved with chromatin remodeling during spermatogenesis [67,68]. The remaining 8 genes (*Clgn *[*Calmegin*], *Tekt1*, *Fscn3*, *Dnahc8*, *Ldhc*, *Adam3 *[*Cyritestin*], *Oaz3*, *Akap3*) have well characterized functions in spermatogenesis [69-76]. Thus, FIGLA appears to play a role in preventing expression of male germ cell associated genes during oogenesis. If true, additional co-factors must interact with FIGLA in determining its affect on downstream gene targets.

## Conclusion

Taken together, these data indicate that FIGLA, an oocyte-specific, basic helix-loop-helix transcription factor, plays a pivotal role in modulating multiple genetic hierarchies involved in folliculogenesis, fertilization and pre-implantation development. Although transcripts accumulate earlier in embryogenesis, FIGLA protein is first detected ~E19 and affects female perinatal gonadogenesis. While some of the effects on expression may be as direct regulator of downstream target genes, others may be indirect through the activation (or suppression) of other transcription regulator(s). In addition to involvement in the activation of gene expression, it seems likely that FIGLA will also down-regulate genes, the expression of which would be inappropriate during post-natal oogenesis. The further characterization of the genes that are differentially regulated by FIGLA should prove useful in defining developmental pathways that affect the postnatal female germ cell. These targets represent not only genes that affect folliculogenesis and fertilization, but also maternal effect genes required for successful completion of early mouse development.

## Methods

### Experimental animals and tissue collection

CF1 mice were obtained commercially and *Figla *homozygous null mice [9] were identified by genotyping tail DNA using three primers (see Additional file 9) in a PCR reaction (95°C 5 min, 94°C 30 sec, 60°C 30 sec, 72°C 30 sec for 28 cycles). RNA was extracted from gonads isolated at embryonic day 12.5 (E12.5), E13.5, E14.5, E15.5, E17.5, E19.5, newborn (NB), 2 days post-partum (2dpp) and 7dpp females; all other tissues were obtained from newborn mice. All experiments were conducted in compliance with the guidelines of the Animal Care and Use Committee of the National Institutes of Health under a Division of Intramural Research, NIDDK approved animal study protocol.

### RNA extraction and labeling

RNA was extracted from tissue using Absolutely RNA RT PCR Miniprep Kit (Stratagene, La Jolla, CA) and its integrity was determined with RNA 6000 Nano Lab-on-Chip (Agilent Technologies, Waldbronn, Germany). For hybridization to microarrays, 50 μg of total RNA (20 ovaries) was linearly amplified with the Aminoallyl RNA Amplification and Labeling System (NuGen Technologies, San Carlos, CA) and labeled with ester-linked Cy3 and Cy5 dyes (Amersham Biosciences, Piscataway, NJ).

### Microarray hybridization

Labeled cDNA probes were hybridized to NIA cDNA microarrays on glass slides containing 20,996 features [12,13] according to Vanderbilt Microarray Shared Resources protocols [77]. In brief, arrays were washed (0.2% SDS) at RT and incubated in a pre-hybridization solution (5 × SSC, 0.1% SDS, 1% BSA) at 55°C for 45 min [78]. After 5 rinses in Milli Q water and 1 in iso-propanol, arrays were air dried and overlaid with coverslips. Utilizing a Maui Hyb Station (BioMicro Systems, Salt Lake City, UT), the arrays were hybridized (25% formamide, 5 × SSC, 0.1% SDS, 1 μg poly(A)^+ ^RNA) at 42°C for 16 h and then washed (5 min, 55°C) sequentially with 2 × SSC, 1 × SSC and 0.1 × SSC, each with 0.1% SDS After drying by centrifugation (20 × g), the arrays were scanned using the Axon 4000 B (Axon Instruments, Union City, CA).

### Microarray data analysis

Hybridizations were performed in triplicate (E12.5, E14.5, E17.5) or quadruplicate (newborn) with dye-swapped pairs of cDNA. The raw data were analyzed with GeneSpring GX 7.2 (Agilent Technologies, Waldbronn, Germany) to remove data that did not meet the following criteria: 1) Cy5 signal to background intensity ratio less than 1.5; 2) Cy3 signal to background intensity ratio less than 1.5; 3) Cy5 signal less than 200; and 4) Cy3 signal less than 200. Data were then normalized by the LOWESS sub-grid method and features with a coefficient of variance of less than 30% (across replicate arrays) and ≥ 2-fold changes between normal and *Figla *null gonads were identified. Analysis of Variance (ANOVA) was performed on the set of genes identified in newborn ovaries. Hierarchical clustering of transcripts during development was determined by GeneSpring GX 7.2.

Alternatively, all eight replicates of newborn ovaries were used in a one-sample Student's t-test to determine whether the mean normalized expression value for each element was statistically different from 1. False Discovery Rate [37] at a threshold of 5% was used as a multiple testing correction to decrease the number of false positives.

### SAGE library construction

Total RNA (~50 μg) was obtained from newborn normal and *Figla *null ovaries with an RNeasy mini kit (Qiagen, Valencia, CA). The presence of MSY2 and the absence of ZP2 transcripts was assayed by one-step qRT-PCR according to the manufacturer's protocol (Qiagen, Valencia, CA) using oligonucleotide primers (see Additional file 3) to confirm the identity of *Figla *null ovaries. Poly(A)^+ ^RNA was isolated with oligo d(T)-conjugated magnetic beads (Dynal-Invitrogen, Carlsbad, CA) and 5 μg was used to construct libraries using a modification [79] of the original SAGE protocol [80]. The final concatenated ditags were cloned into Bluescript pKS (Stratagene, La Jolla, CA) using the BamHI site for blue-white screening. Plating, picking, DNA preparation and sequencing were performed at the NIH Intramural Sequencing Center (NISC). Low quality sequence data were eliminated by Phred analysis.

eSAGE [81] was used to extract tags and remove duplicate ditags from the raw sequencing data. 79,095 tags from the normal were compared to 77,851 tags from the *Figla *null libraries to determine statistically significant differences either by the Audic and Claverie test [20] using eSAGE [82] or Fisher's exact test [83] using IDEG6 [21]. Tags with ρ ≤ 0.05 were extracted from both lists and further analysis was performed on tags present at least 10 times in normal and absent from *Figla *null ovaries. These lists of tags were then mapped to Unigene clusters using reliable mapping from NCBI Unigne build #138. A group of 13 genes from the microarray data and 8 from the SAGE analysis that were up-regulated in normal newborn ovaries were selected for more detailed analysis based on their expression profile in the SOURCE data base at the Genetics Department at Stanford University [23].

### Analysis of gene expression

Transcript abundance was determined by quantitative real-time polymerase chain reaction (qRT-PCR) on an ABI 7700 HT Sequence Detection System (Applied Biosystems, Foster City, CA) using QuantiTect SyBR green PCR kit (Qiagen, Valencia, CA) and primers (see Additional file 3) designed by Primer Express 2.0 (Applied Biosystems, Foster City, CA). Independently obtained biological samples (2–6), analyzed in triplicate were normalized to HPRT (averaged for tissue or developmental time point) and expressed as an average (± s.e.m). PCR products from the SAGE analysis were separated by agarose gel electrophoresis and stained with ethidium bromide (0.1 ug/ml). Tissue-specific expression for the in situ hybridization was performed on ovarian sections obtained from normal, 6–8 week old mice. Ovaries were fixed (4% paraformaldehyde, PBS, pH 7.4), and processed for paraffin sections (5 μm) which were then re-hydrated stepwise through alcohol washes (100%, 70%, 50%, 30%) and distilled water prior to PBS. Sections were hybridized with sense or antisense probes (200 ng/ml) (see Additional file 4) labeled with digoxigenin (DIG) according to the manufacturer's instructions (GenPoint, Dako, Carpenteria, CA) except for the use of rabbit anti-DIG-HRP antibodies (1:200, Dako, Carpenteria, CA). Sections were then counter-stained with hematoxylin and de-hydrated stepwise in ethanol (30%, 50%, 70%, 100%) prior to permount mounting (Fisher Scientific, Fair Lawn, NJ). Images were acquired with an Axioplan microscope equipped with a CCD camera using AxioVision software (Carl Zeiss, Gottingen, Germany).

## Authors' contributions

SJ isolated RNA from developmentally staged gonads to probe microarrays, participated in evaluation of the microarray experiments, provided analysis of expression patterns of selected genes and wrote the initial draft of the manuscript. HD constructed SAGE libraries, compared the transcriptomes of normal and *Figla *null newborn ovaries, analyzed expression patterns of selected genes and co-wrote the manuscript. LPS performed the microarray analysis and provided statistical interpretation of the results. SEL participated in the initial design of the microarray analysis and subsequent interpretation of the results. JD conceived the project, provided analysis and interpretation of the data and co-wrote the manuscript. All authors read and approved the final manuscript.

## Supplementary Material

Additional file 1 NIA microarray: genes potentially up-regulated by FIGLA Click here for file

Additional file 2 NIA microarray: genes potentially down-regulated by FIGLA Click here for file

Additional file 3 List of primers used for qRT-PCR Click here for file

Additional file 4 Oligonucleotides used for in situ hybridization (5'-3') Click here for file

Additional file 5 False discovery rate analysis of newborn microarray: genes potentially up-regulated by FIGLA Click here for file

Additional file 6 False discovery rate analysis of newborn microarray: genes potentially down-regulated by FIGLA Click here for file

Additional file 7 SAGE libraries: genes potentially up-regulated by FIGLA Click here for file

Additional file 8 SAGE libraries: genes potentially down-regulated by FIGLA Click here for file

Additional file 9 Primers used for genotyping *Figla *null mice (5'-3') Click here for file
