# Supplementary material for: Ovarian gene expression in the absence of FIGLA, an oocyte-specific transcription factor
Source: BMC Dev Biol. 2007 Jun 13;7:67. doi: 10.1186/1471-213X-7-67 (PMC1906760; doi:10.1186/1471-213X-7-67)
Supplement: Additional file 1 — NIA microarray: genes potentially up-regulated by FIGLA [file 1471-213X-7-67-S1.pdf]

Additional file 1 - NIA microarray: genes potentially up-regulated by FIGLA

	NIA	ρ ≤	Common	Genbank	Unigene	Name
	H3104H01	0.0000	C86187	AA692270	Mm.22314	Transcribed locus
	H3142B02	0.0000	Ndufa7	BQ951360	Mm.29513	NADH dehydrogenase (ubiquinone) 1 alpha subcomplex, 7 (B14.5a) (Ndufa7), mRNA
	H3151D02	0.0000	data not found	data not found	data not found	data not found
	H3151G09	0.0000	Pcdhga12	AF464160	Mm.247203	Protocadherin gamma subfamily A, 10, mRNA (cDNA clone MGC:40648 IMAGE:5400956)
	H3087F07	0.0000	data not found	data not found	data not found	data not found
●	H3136A01	0.0000	Kit	BC026713	Mm.247073	Kit oncogene (Kit), mRNA
	H3055B02	0.0000	data not found	data not found	data not found	data not found
►	H3080G03	0.0000	C330026N02Rik	NM_175290	Mm.315815	NALP-kappa (Nalp-kappa)
	H3130A02	0.0000	data not found	data not found	data not found	data not found
	H3134D03	0.0000	Nmnat3	BC005737	Mm.294082	Nicotinamide nucleotide adenylyltransferase 3 (Nmnat3), mRNA
	H3060D08	0.0000	data not found	data not found	data not found	data not found
	H3158G01	0.0000	data not found	data not found	data not found	data not found
	H3128E01	0.0000	data not found	data not found	data not found	data not found
	H3144A01	0.0000	4930562C15Rik	XM_489501	Mm.325551	PREDICTED: hypothetical protein LOC78809 [Mus musculus], mRNA sequence
	H3141C02	0.0000	AL022943	BU515331	Mm.262730	Transcribed locus
	H3130A01	0.0000	data not found	data not found	data not found	data not found
	H3133D07	0.0000	4930403J22Rik	AK033947	Mm.271988	RIKEN cDNA 4930403J22 gene, mRNA (cDNA clone MGC:32342 IMAGE:5029461)
	553026	0.0000	Top3b	AB045324	Mm.326089	Topoisomerase (DNA) III beta, mRNA (cDNA clone MGC:30218 IMAGE:5003002)
	H3067A09	0.0000	Zdhhc13	BC046599	Mm.279116	Zinc finger, DHHC domain containing 13, mRNA (cDNA clone MGC:54871 IMAGE:6311721)
	H3061F01	0.0000	4930562C15Rik	XM_489501	Mm.325551	PREDICTED: hypothetical protein LOC78809 [Mus musculus], mRNA sequence
	H3051D02	0.0000	Exo1	AK028728	Mm.283046	Exonuclease 1, mRNA (cDNA clone MGC:5686 IMAGE:3499406)
	H3135G08	0.0000	data not found	BC084590	Mm.328014	1 cell embryo 1 cell cDNA, RIKEN full-length enriched library, clone:I0C0022N05
	H3128G07	0.0000	data not found	data not found	data not found	data not found
	H3151B06	0.0000	data not found	data not found	data not found	data not found
	H3103D02	0.0000	BC040823	BC040823	Mm.21577	CDNA sequence BC040823, mRNA (cDNA clone MGC:117552 IMAGE:30933544)
●	H3157G03	0.0000	Og2x	AY061761	Mm.17715	OG2 homeobox gene (Og2x), mRNA
	H3129H12	0.0000	data not found	data not found	data not found	data not found
	H3129D01	0.0000	data not found	data not found	data not found	data not found
	H3051D10	0.0000	data not found	data not found	data not found	data not found
	720076	0.0000	data not found	AI464807	Mm.361796	Adult male cecum cDNA, RIKEN full-length enriched library,

	NIA	ρ ≤	Common	Genbank	Unigene	Name
						clone:9130020F13
	H3055G11	0.0000	data not found	data not found	data not found	data not found
	H3082H03	0.0000	Helic1	XM_125617	Mm.222497	Activating signal cointegrator 1 complex subunit 3, mRNA (cDNA clone IMAGE:5321369)
►	H3078C06	0.0000	Arhgap20	NM_175535	Mm.26150	Rho GTPase activating protein 20, mRNA (cDNA clone IMAGE:5363167)
	H3129H11	0.0000	data not found	data not found	data not found	data not found
	H3158B05	0.0000	Tes3-ps	BC024630	Mm.260975	Testis derived transcript 3, mRNA (cDNA clone MGC:28191 IMAGE:3988680)
	H3159F10	0.0000	Dhx40	AK010512	Mm.260627	DEAH (Asp-Glu-Ala-His) box polypeptide 40 (Dhx40), mRNA
●	H3138G05	0.0000	Zp2	M34148	Mm.6510	Zona pellucida glycoprotein 2 (Zp2), mRNA
●	H3102C08	0.0000	Zp3	M20026	Mm.1381	Zona pellucida glycoprotein 3 (Zp3), mRNA
►	H3028H01	0.0000	Pou5f1	BC068268	Mm.17031	POU domain, class 5, transcription factor 1 (Pou5f1), mRNA
	H3151F06	0.0000	Xdh	NM_011723	Mm.11223	Similar to hypothetical protein MGC37588, mRNA (cDNA clone MGC:28125 IMAGE:3980327)
	H3142A06	0.0000	Mphosph6	BC069961	Mm.181836	M phase phosphoprotein 6 (Mphosph6), mRNA
	H3086A06	0.0000	data not found	data not found	data not found	data not found
	H3145A06	0.0000	Slc30a3	BC066199	Mm.1396|Mm.381478	13 days embryo brain cDNA, RIKEN full-length enriched library, clone:G630099F21
	H3073G11	0.0000	data not found	AA822730	data not found	data not found
	H3080F08	0.0000	data not found	data not found	data not found	data not found
	H3070B01	0.0000	Zbed3	BC085478	Mm.3774	Zinc finger, BED domain containing 3 (Zbed3), mRNA
	H3085E05	0.0000	4930487N19Rik	XM_283206	Mm.275688	RIKEN cDNA 4930487N19 gene, mRNA (cDNA clone MGC:63131 IMAGE:3470278)
	H3010B02	0.0000	data not found	data not found	data not found	data not found
	551688	0.0000	Prep	BC050830	Mm.37294	Prolyl endopeptidase, mRNA (cDNA clone MGC:11413 IMAGE:3967219)
	H3149H09	0.0000	1700006H03Rik	CN834482	Mm.325325	PREDICTED: hypothetical protein LOC74174 [Mus musculus], mRNA sequence
	H3061A04	0.0001	6720468P15Rik	AK078497	Mm.12411	0 day neonate thymus cDNA, RIKEN full-length enriched library, clone:A430057M21
	585251	0.0001	Cpeb1	Y08260	Mm.273122	Cytoplasmic polyadenylation element binding protein 1 (Cpeb1), mRNA
	H3056D08	0.0001	5830484A20Rik	BF098849	Mm.351825|Mm.379676	PREDICTED: RIKEN cDNA 4930565N07 [Mus musculus],
	H3060B10	0.0001	data not found	data not found	data not found	data not found
	H3025C02	0.0001	data not found	Multiple clusters	Mm.311913	RIKEN cDNA 7420416P09 gene (7420416P09Rik), mRNA
	H3156G03	0.0001	4931419K03Rik	AK036610	Mm.235443	RIKEN cDNA 4931419K03 gene, mRNA (cDNA clone MGC:67711 IMAGE:4035998)
	H3051G02	0.0001	Cpeb3	AB093274	Mm.360051	Cytoplasmic polyadenylation element binding protein 3 (Cpeb3), mRNA
	H3134E07	0.0001	data not found	AK078310	Mm.300962	PREDICTED: Mus musculus hypothetical protein LOC (LOC544755),

	NIA	ρ ≤	Common	Genbank	Unigene	Name
						mRNA
●	H3099F09	0.0001	Dppa5	Multiple clusters	Mm.139314|Mm.359437	Developmental pluripotency associated 5 (Dppa5), mRNA | Transcribed locus
	H3088A09	0.0001	1700057H21Rik	BG070503	Mm.250471	Transcribed locus
►	H3102E07	0.0001	Nalp5	CO804754	Mm.11741|Mm.333653	MATER (Maternal Antigen That Embryos Require), NALP5
	521770	0.0001	Ctrc	AI386066	Mm.308195	Chymotrypsin C (caldecrin) (Ctrc), mRNA
	H3134A10	0.0001	4930562C15Rik	XM_489501	Mm.325551	PREDICTED: hypothetical protein LOC78809 [Mus musculus], mRNA sequence
	H3046D12	0.0001	data not found	data not found	data not found	data not found
	H3035E10	0.0001	data not found	data not found	data not found	data not found
	551381	0.0001	Osbpl8	NM_175489	Mm.220204	Oxysterol binding protein-like 8 (Osbpl8), transcript variant 2, mRNA
	H3051E04	0.0001	data not found	Multiple clusters	data not found	data not found
	H3152B03	0.0001	Grid2	AK046841	data not found	data not found
	H3019H10	0.0001	Zbtb10	AK047899	Mm.103262	PREDICTED: zinc finger and BTB domain containing 10 [Mus musculus], mRNA sequence
	H3108C05	0.0001	data not found	data not found	data not found	data not found
►	H3046B08	0.0001	C330003B14Rik	AK049114	Mm.201536	RIKEN cDNA C330003B14 gene (C330003B14Rik), mRNA
	634968	0.0001	Slc27a2	BC013442	Mm.290044	Solute carrier family 27 (fatty acid transporter), member 2, mRNA (cDNA clone MGC:29967 IMAGE:5123650)
	552702	0.0001	data not found	BB505010	Mm.23581	Transcribed locus
	H3064C07	0.0001	AI427122	XM_110660	Mm.11869	Expressed sequence AI427122, mRNA (cDNA clone IMAGE:4216549)
	H3061A05	0.0001	Zbtb10	AK047899	Mm.103262	PREDICTED: zinc finger and BTB domain containing 10 [Mus musculus], mRNA sequence
	H3050H08	0.0001	BC031748	BC031748	Mm.260106	CDNA sequence BC031748 (BC031748), mRNA
	H3101A03	0.0002	Rnf35	BC064738	Mm.28010	Tripartite motif-containing 61 (Trim61), mRNA
	H3114B04	0.0002	Rdx	BC034438	Mm.245746	Radixin (Rdx), mRNA
	583647	0.0002	Kif14	XM_355234	Mm.246505	PREDICTED: Mus musculus kinesin family member 14 (Kif14), mRNA
►	H3075E03	0.0002	E330017A01Rik	CO814438	Mm.26145	RIKEN cDNA E330017A01 gene, mRNA (cDNA clone MGC:117520 IMAGE:30936622)
	H3107D12	0.0002	data not found	data not found	data not found	data not found
	H3158G04	0.0002	Stat3	AK079406	Mm.249934	Signal transducer and activator of transcription 3, mRNA (cDNA clone IMAGE:3665873)
	H3083D07	0.0002	data not found	data not found	data not found	data not found
	H3065G12	0.0003	Epb4.1l5	AK173198	Mm.253156	Erythrocyte protein band 4.1-like 5 (Epb4.1l5), mRNA
	H3092H04	0.0003	data not found	data not found	data not found	data not found
	H3060B07	0.0003	Ndg1	AY238603	Mm.26006	Nur77 downstream gene 1 (Ndg1), mRNA
	H3051H08	0.0004	Btg4	AB050983	Mm.104932	B-cell translocation gene 4 (Btg4), mRNA
	551387	0.0004	Tnfaip8	AK079518	Mm.27740	Tumor necrosis factor, alpha-induced protein 8 (Tnfaip8), mRNA
	H3058G06	0.0004	Trpm7	AY032951	Mm.244705	LTRPC7 (Ltrpc7)

	NIA	ρ ≤	Common	Genbank	Unigene	Name
	H3064G10	0.0004	Zbed3	BC085478	Mm.3774	Zinc finger, BED domain containing 3 (Zbed3), mRNA
	636958	0.0004	data not found	CK030712	Mm.337716	6 days neonate spleen cDNA, RIKEN full-length enriched library, clone:F430003O07
	H3135D05	0.0004	Col9a3	AK030350	Mm.141312	Procollagen, type IX, alpha 3, mRNA (cDNA clone MGC:32160 IMAGE:5003028)
	H3040F10	0.0005	Lmo7	BC082553	Mm.218981	PREDICTED: LIM domain only 7 [Mus musculus], mRNA sequence
	H3089D03	0.0005	data not found	data not found	data not found	data not found
	598513	0.0005	data not found	Multiple clusters	Mm.326609|Mm.327001	MRNA expressed in islet cells (clone 43)| Transcribed locus
	H3055E10	0.0005	data not found	Multiple clusters	Mm.362671|Mm.335592	PREDICTED: Mus musculus similar to RIKEN cDNA 5830484A20 (LOC545341), mRNA| Transcribed Locus
	H3060D03	0.0006	D7Ertd445e	BC054740	Mm.223639	Phospholipase A2, group IVC (cytosolic, calcium-independent) (Pla2g4c), mRNA
	H3069B11	0.0006	data not found	AU023774	Mm.354103	Transcribed locus
	H3078D03	0.0006	Ndrg4	AK090374	Mm.29846	N-myc downstream regulated gene 4 (Ndrg4), mRNA
	H3135G06	0.0006	Aof1	NM_172262	Mm.31259	Amine oxidase, flavin containing 1, mRNA (cDNA clone MGC:38211 IMAGE:5323227)
	H3081C03	0.0006	Zbtb10	AK047899	Mm.103262	PREDICTED: zinc finger and BTB domain containing 10 [Mus musculus], mRNA sequence
	H3152H12	0.0008	Pard3	AK053576	Mm.299254	Par-3 (partitioning defective 3) homolog (C. elegans) (Pard3), transcript variant 3, mRNA
	H3078G11	0.0008	Drd3	X67274	Mm.327835	Dopamine receptor 3 (Drd3), mRNA
	H3041C12	0.0008	data not found	data not found	data not found	data not found
	H3069B10	0.0009	data not found	data not found	data not found	data not found
	H3135F10	0.0010	data not found	XM_356199	Mm.384700|Mm.17857	10 days embryo whole body cDNA, RIKEN full-length enriched library
	338143	0.0010	2610020H15Rik	BC016541	Mm.281887	Glycerophosphodiester phosphodiesterase domain containing 1 (Gdpd1), mRNA
	H3156B04	0.0011	Chn1	AK049943	Mm.257073	Chimerin (chimaerin) 1, mRNA (cDNA clone MGC:39019 IMAGE:5364554)
	H3101A12	0.0012	Ccrn4l	U70139	Mm.86541	NOCTURNIN (Nocturnin)
	H3072H12	0.0012	E2f5	AK028856	Mm.153415	E2F transcription factor 5, mRNA (cDNA clone MGC:6043 IMAGE:3482306)
	H3068H04	0.0013	LOC235779	Multiple clusters	Mm.296705|Mm.37128	Hypothetical LOC235779 (LOC235779), mRNA
	H3043D08	0.0016	data not found	BG066474	Mm.318680	Transcribed locus
	H3110B11	0.0016	2900002G04Rik	XM_136135	Mm.160079	PREDICTED: hypothetical protein LOC78283 [Mus musculus], mRNA sequence
	H3058B06	0.0016	data not found	data not found	data not found	data not found
	315658	0.0016	Thedc1	AK087571	Mm.13808	Thioesterase domain containing 1 (Thedc1), mRNA
	H3058B02	0.0017	Tpd52	AK032111	Mm.371590	Tumor protein D52 (Tpd52), transcript variant 1, mRNA
	H3092D04	0.0018	data not found	data not found	data not found	data not found
	597152	0.0018	4930487N19Rik	XM_283206	Mm.275688	RIKEN cDNA 4930487N19 gene, mRNA (cDNA clone MGC:63131

	NIA	ρ ≤	Common	Genbank	Unigene	Name
						IMAGE:3470278)
	H3071F03	0.0018	Nexn	AK028732	Mm.200188	Nexilin (Nexn), mRNA
	H3058H01	0.0018	Oas1c	AK028986	Mm.43230	2'-5'olygoadenylate synthetase 1c (Oas1c) mRNA, Oas1c-RV allele
	H3071A11	0.0019	E430034L04Rik	BC048176	Mm.290530	RNA-binding protein isoform G3BP-2a (G3BP2)
	555534	0.0022	Fbxo21	AK049723	Mm.21912	F-box only protein 21 (Fbxo21), mRNA
	552704	0.0024	Tor3a	BC052851	Mm.206737	Torsin family 3, member A (Tor3a), mRNA
►	H3154E08	0.0024	Pdzk1	AK029764	Mm.28015	Hydrophilic CFTR-binding protein CAP70 (Cap70)
►	H3087F10	0.0024	Elavl2	AK083865	Mm.318042	ELAV (embryonic lethal, abnormal vision, Drosophila)-like 2 (Hu antigen B) (Elavl2), transcript variant 2, mRNA
►	H3070F03	0.0024	AU022726	BC066795	Mm.95244	NALP-gamma (Nalp-gamma)
	H3158A11	0.0025	Kif16b	Multiple clusters	Mm.251934	MKIAA1590 protein
	H3137D12	0.0025	Vkorc1	CF585248	Mm.29703	Vitamin K epoxide reductase complex, subunit 1, mRNA (cDNA clone MGC:25747 IMAGE:3991412)
	H3089D11	0.0026	data not found	data not found	data not found	data not found
	H3111B10	0.0026	Ap3b2; Naptb; [b]-NAP; MGC36656; beta-NAP	NM_021492	Mm.322894	Adaptor-related protein complex 3, beta 2 subunit, mRNA (cDNA clone IMAGE:5364848)
	571878	0.0026	4930403J22Rik	AK033947	Mm.271988	RIKEN cDNA 4930403J22 gene, mRNA (cDNA clone MGC:32342 IMAGE:5029461)
	H3056E12	0.0028	data not found	data not found	data not found	data not found
	H3100G04	0.0031	data not found	data not found	data not found	data not found
	H3103G12	0.0033	data not found	data not found	data not found	data not found
	H3093F04	0.0034	Ddx20	NM_017397	Mm.272826	DEAD (Asp-Glu-Ala-Asp) box polypeptide 20 (Ddx20), mRNA
	H3059H12	0.0036	Gm99	CO810251	Mm.26881	Gene model 99, (NCBI), mRNA (cDNA clone MGC:117750 IMAGE:30931566)
	H3101B05	0.0038	9930116P15Rik	XM_127961	Mm.297862	PREDICTED: RIKEN cDNA 4930461P20 [Mus musculus], mRNA sequence
	H3134D06	0.0039	Grpel2	BC068232	Mm.269657|Mm.322342	GrpE-like 2, mitochondrial (Grpel2), mRNA
	H3070F06	0.0056	Ipmk	BC052463	Mm.245867	Inositol polyphosphate multikinase (Ipmk), mRNA
	H3093C05	0.0056	data not found	data not found	data not found	data not found
	H3076H06	0.0059	Ak4	BC086663	Mm.42040	Adenylate kinase 3 alpha-like 1 (Ak3l1), mRNA
	H3011D10	0.0061	Lcp1	NM_008879	Mm.153911	Lymphocyte cytosolic protein 1, mRNA (cDNA clone MGC:30234 IMAGE:3486993)
	H3038H06	0.0062	Rex2	BC050018	Mm.379641	PREDICTED: similar to RIKEN cDNA 6330416L07 gene [Mus musculus], mRNA sequence
	H3027E04	0.0063	Bcat1	AK036309	Mm.4606	Branched chain aminotransferase 1, cytosolic (Bcat1), transcript variant 2, mRNA
	H3058B10	0.0063	data not found	Multiple clusters	Mm.25493|Mm.344708	Centrin 4 (Cetn4), mRNA

	NIA	ρ ≤	Common	Genbank	Unigene	Name
	651495	0.0067	4930429O20Rik	AK015245	Mm.34611	RIKEN cDNA 4930429O20 gene (4930429O20Rik), mRNA
►	H3064D03	0.0068	E330034G19Rik	XM_138939	Mm.2077	PREDICTED: polymerase (RNA) III (DNA directed) polypeptide A, 155kDa [Mus musculus], mRNA sequence
	636116	0.0074	Aass	AK044323	Mm.18651	Aminoadipate-semialdehyde synthase, mRNA (cDNA clone MGC:5717 IMAGE:3600654)
	571270	0.0080	Cpeb3	AB093274	Mm.360051	Cytoplasmic polyadenylation element binding protein 3 (Cpeb3), mRNA
	597752	0.0082	Herc3	AK122192	Mm.386815	Hect domain and RLD 3 (Herc3), mRNA
	H3120D12	0.0091	Zfp313	BC085146	Mm.22225	Zinc-finger protein ZFP313 (Zfp313)
	598406	0.0099	Rock1	U58512	Mm.6710	Rho-associated coiled-coil forming kinase 1 (Rock1), mRNA
	H3044A12	0.0099	data not found	data not found	data not found	data not found
	H3093G05	0.0117	6230405M12Rik	AK031754	Mm.173186	PREDICTED: Mus musculus RIKEN cDNA C130092O11 gene (C130092O11Rik), mRNA
	H3058E02	0.0117	data not found	data not found	data not found	data not found
	H3159C05	0.0176	Rab3d	AF263365	Mm.260157	RAB3D, member RAS oncogene family, mRNA (cDNA clone MGC:6707 IMAGE:3584902)
	H3096F11	0.0219	Chst10	BC056956	Mm.260054	Carbohydrate sulfotransferase 10 (Chst10), mRNA
►	H3084F06	0.0219	E330009P21Rik	XM_356191	Mm.235303	PREDICTED: Mus musculus similar to RIKEN cDNA E330009P21 gene (LOC382106), mRNA
	H3051H06	0.0303	data not found	Multiple clusters	Mm.161448	In vitro fertilized eggs cDNA, RIKEN full-length enriched library, clone:7420404A13
	H3108C04	0.0305	data not found	data not found	data not found	data not found
	H3082C04	0.0355	9030611O19Rik	BC085288	Mm.248938	RIKEN cDNA 9030611O19 gene (9030611O19Rik), mRNA
	H3001C08	0.0355	data not found	data not found	data not found	data not found
	H3060H03	0.0385	Tcl1	BC052336	Mm.18154	T-cell lymphoma breakpoint 1 (Tcl1), mRNA
►	H3033B11	0.0438	Serpinb6c	BC062169	Mm.272188	Serine (or cysteine) peptidase inhibitor, clade B, member 6c (Serpinb6c), mRNA
►	Genes highlighted in paper					
●	Previously reported ovary-specific genes					
