# Supplementary material for: Ovarian gene expression in the absence of FIGLA, an oocyte-specific transcription factor
Source: BMC Dev Biol. 2007 Jun 13;7:67. doi: 10.1186/1471-213X-7-67 (PMC1906760; doi:10.1186/1471-213X-7-67)
Supplement: Additional file 2 — NIA microarray: genes potentially down-regulated by FIGLA [file 1471-213X-7-67-S2.pdf]

Additional file 2 - NIA microarrays: genes potentially down-regulated by FIGLA

	NIA	ρ ≤	Symbol	Genbank	UniGene	Name
■	H3154D11	0.0002	Taf7l	AK017109	Mm.103259	TAF7-like RNA polymerase II, TATA box binding protein (TBP)-associated factor (Taf7l), mRNA
	H3121A01	0.0006	Ncam2	AF001286	Mm.258759	Neural cell adhesion molecule 2 (Ncam2), mRNA
	H3123F10	0.0007	data not found	data not found	data not found	M.musculus DNA for alpha globin gene and flanking regions
	H3124F12	0.0017	data not found	data not found	data not found	data not found
	427063	0.0025	2810417M05Rik	AK014196	Mm.357108	RIKEN cDNA 2810417M05 gene (2810417M05Rik), mRNA
■	H3083F09	0.0025	Phtf1	BC057340	Mm.378981	Putative homeodomain transcription factor 1 (Phtf1), mRNA
	H3045A12	0.0028	Hba-a1	BF144167	Mm.196110	Hemoglobin alpha, adult chain 1 (Hba-a1), mRNA
■	H3091F07	0.0032	Gm1564	XM_196054	Mm.297760	PREDICTED: similar to FLJ35848 protein [Mus musculus], mRNA sequence
	H3113B02	0.0040	5730411O18Rik	AK077457	Mm.260866	RIKEN cDNA 5730411O18 gene (5730411O18Rik), mRNA
	334924	0.0070	Ilvbl	BC052054	Mm.2644	IlvB (bacterial acetolactate synthase)-like, mRNA (cDNA clone MGC:67151 IMAGE:5718157)
	H3118D11	0.0095	data not found	data not found	data not found	data not found
	604356	0.0108	data not found	Multiple clusters	Mm.8766|Mm.253	ESTs, Highly similar to AF157318_1 AD-017 protein [H.sapiens]
	662847	0.0135	1110031M08Rik	XM_355145	Mm.347805	PREDICTED: hypothetical protein LOC68693 [Mus musculus], mRNA sequence
	614363	0.0137	2810441O16Rik	Multiple clusters	Mm.316928	RNA-binding region (RNP1, RRM) containing 3 (Rnpc3), mRNA
	H3146G12	0.0137	C330046E03	BC025885	Mm.326569	Transcribed locus
	H3118E11	0.0150	data not found	data not found	data not found	Mouse beta-globin major gene
	H3114C08	0.0155	Eraf	AW107911	Mm.218857	Erythroid associated factor (Eraf), mRNA
	H3121D10	0.0176	Mrg1	AK040611	Mm.247566	Myeloid ecotropic viral integration site-related gene 1, mRNA (cDNA clone MGC:13943 IMAGE:4191098)
■	H3120F02	0.0186	Tia1	BC027826	Mm.30849|Mm.33 3219	Human polyadenylate binding protein (TIA-1) mRNA, complete cds
	H3087C05	0.0201	data not found	data not found	data not found	data not found
	H3073B09	0.0201	data not found	data not found	data not found	data not found
	H3126B09	0.0201	data not found	data not found	data not found	data not found
	313981	0.0201	data not found	Multiple clusters	Mm.288567 Mm.2444 Mm.387214	hemoglobin, beta adult major chain
	H3057H09	0.0201	data not found	data not found	data not found	data not found
	H3134E01	0.0201	Hbb-b1	BQ044124	Mm.288567	Hemoglobin, beta adult major chain, mRNA (cDNA clone MGC:40691 IMAGE:3988455)
	H3157D01	0.0201	Got2	AK098166	Mm.230169|Mm.3 83179	Glutamate oxaloacetate transaminase 2, mitochondrial (Got2), mRNA
	H3114C04	0.0201	Trim44	BC039979	Mm.315002	Tripartite motif-containing 44 (Trim44), mRNA
	H3118G01	0.0214	data not found	data not found	data not found	Mouse gene for beta-1-globin

	NIA	ρ ≤	Symbol	Genbank	UniGene	Name
	H3073B08	0.0216	data not found	data not found	data not found	data not found
	H3073D08	0.0302	data not found	data not found	data not found	data not found
	H3061F03	0.0302	data not found	data not found	data not found	Homo sapiens jagged 1 (Alagille syndrome) (JAG1), mRNA
	H3065F02	0.0302	A830008O07	AK081241	Mm.126450	Hypothetical protein A830008O07 (A830008O07), mRNA
	615776	0.0318	Ppp2r5e	NM_012024	Mm.259626	MKIAA4006 protein
	H3065A01	0.0318	data not found	data not found	data not found	Mus musculus exportin 4 (Xpo4-pending), mRNA
	H3070D08	0.0334	data not found	data not found	data not found	data not found
	H3056F05	0.0334	data not found	data not found	data not found	data not found
	H3073C09	0.0391	data not found	data not found	data not found	data not found
	H3013H08	0.0391	data not found	data not found	data not found	data not found
■	Testis-specific genes					
