# Supplementary material for: Ovarian gene expression in the absence of FIGLA, an oocyte-specific transcription factor
Source: BMC Dev Biol. 2007 Jun 13;7:67. doi: 10.1186/1471-213X-7-67 (PMC1906760; doi:10.1186/1471-213X-7-67)
Supplement: Additional file 3 — List of primers used for qRT-PCR [file 1471-213X-7-67-S3.pdf]

Additional file 3 - List of primers used for qRT-PCR

**Name of gene**	**Forward primer (5`-3`)**	**Reverse primer (5`-3`)**
BC052883	CTTTGGACCCCAGTGAAGAATAAG	TGTGCTACTTTGGCCTATGGATT
C330003B14Rik	TGGAGCCACTGAGAAGGTCAA	AGCCTCCAGGGCCTCAGTAG
E330034G19Rik	GGGAAGCAGACCCAGAAGAAG	GTGAATAGGCCCTGATTTTATGGA
E330017A01Rik	TGAATCAGCCAGGGTTTCAAA	CATGACTGGCAAGAAGGAGACTT
E33000P21Rik	CCATGTAAATCACTGAAAGCTGTCA	AGATATCGCCTGCCAAGACAAT
Nalp4a (ita)	AAGACCAGCTGCAGGGCTTAT	CACAAACGTGTCAGTCCACATG
Nalp4f (kappa)	GCCCATGGTTCTTGCATCA	ATCAGAGGCAGTGTGGAAGCA
Nalp4b	GAGTGTCTGAAACTGTAGTGCGAAA	TTTTTGCCACATTCAGATGAGTTTT
Nalp 5 (Mater)	GCTTCCACAGGCCAATTATCC	GCTGTGCTCTGCGTTCCAA
Nalp14 (iota)	CCAGCAGCCACACTGCAAT	ACAAGCCTTACTCGTGAGAACACA
Oas1d	TTGGAAGCTAAGGCAGACGAA	CCTGCCTGCCCATGGA
Serpineb6C	TCCACCAGGGCTTCCAGTT	GCGAGTACTGTGTGCCAGTCTT
Arhgap20	GCCCGATTCCCTTGCAA	CCCACCGAATACATATACACCAAA
Elavl2	CATGGCCGCCTCATCATAG	TTTAACACCAATAAGTGCAAAGGTTT
Pdzk1	TGCGAGGCTGTGCTGAGA	TCTGCGGAGTCCGAGCAT
Figla	CCAAAGAGCGTGAACGGATAA	TCTTCCAGAACACAGCCGAGT
Pou5f1	CCAATCAGCTTGGGCTAGAG	CCTGGGAAAGGTGTCCTGTA
Dppa3	CTTTGTTGTCGGTGCTGAAA	TCCCGTTCAAACTCATTTCC
Oas1h	GTCCAGAACAGACGTGAACTCC	TCGATTGTAGGAACCACCCA
Padi6	TGTTTCTCACCGGCATCGA	ATTCACAAGCAGGATGGCTCC
AK087784	CCAGAGCGATTGACATGAAA	ATTTCTCACTAGCGGGAGCA
2410146L05Rik	AAACGAACTCCAAAGAGCTGTG	AAAGCCAGCCAGTTTTAGCC
BG074389	CAGGGAGCTCAGTGCTAAGG	CGAAAGAGACGAGACCCAAA
AK139812	ATTCCCTCTGAAGGTGCCAGA	AGATGGCACACAGAGAGGTGGT
Hprt	CAGGCCAGACTTTGTTTGGAT	TTGCGCTCATCTTAGGCTTT
Zp2	GGGAAAACCCACCCTCCA	GCCACAGCACCCAGTGTT
Msy	GCACCATTGGAGGGTGATCAACAGC	GATCCCTTCCTTCAACCCATGCTAG

Conditions: 95°C 10 min, 95°C 30 sec, 55°C 30 sec, 72°C 30 sec for 40 cycles
Dissociation step: 95°C 30 sec, 60°C 30 sec, 95°C 30 sec for 1 cycle
