# Supplementary material for: Ovarian gene expression in the absence of FIGLA, an oocyte-specific transcription factor
Source: BMC Dev Biol. 2007 Jun 13;7:67. doi: 10.1186/1471-213X-7-67 (PMC1906760; doi:10.1186/1471-213X-7-67)
Supplement: Additional file 4 — Oligonucleotides used for in situ hybridization (5'-3') [file 1471-213X-7-67-S4.pdf]

Additional file 4 - Oligonucleotides used for in situ hybridization (5`- 3`)

BC052883	CTTCCACTTTAGGTTTGCTGATTGATGTCGCGTGGCCCAGGTCACCAA
E330034G19rik	TTCCTCCGTTCTTTCTGGATCCTGCTGCTGGGTCTGGGGGTCACT
C330003B14rik	TTCCAGCGGCCCCTTGATGCTCCACAGTGCCAATTGATTGCCTTCCGA
E330017A01rik	GGAAAAGTCTCCTTCTTGCCAGTCATGAAACTGTGGGCCATTGCAAAA
E330009P21rik	TCCATTTCGGGTTCTCATTGTAGTCTTGCAAAGAGAAACTCGCTACCA
