# Supplementary material for: Ovarian gene expression in the absence of FIGLA, an oocyte-specific transcription factor
Source: BMC Dev Biol. 2007 Jun 13;7:67. doi: 10.1186/1471-213X-7-67 (PMC1906760; doi:10.1186/1471-213X-7-67)
Supplement: Additional file 5 — False discovery rate analysis of newborn microarray: genes potentially up-regulated by FIGLA [file 1471-213X-7-67-S5.pdf]

Additional file 5 – False discovery rate analysis of newborn microarray: genes potentially up-regulated by FIGLA

	NIA	ρ ≤	Common	Unigene	Name
	H3141C02	0.0004	AL022943	Mm.262730	Expressed sequence AL022943
	H3136A01	0.0004	Kit	Mm.247073	Kit oncogene
	H3103D02	0.0004	Lsm14b	Mm.21577	LSM14 homolog B
	H3142B02	0.0004	Ndufa7	Mm.29513	NADH dehydrogenase (ubiquinone) 1 alpha subcomplex, 7 (B14.5a)
	H3150C01	0.0004	G3bp2	Mm.290530	GTPase activating protein (SH3 domain) binding protein 2
	H3158B05	0.0004	Ippk	Mm.390586	Inositol 1,3,4,5,6-pentakisphosphate 2-kinase
	H3151G09	0.0004	Pcdhgc3	Mm.247203	Protocadherin gamma subfamily C, 3
	H3101A03	0.0004	Trim61	Mm.28010	Tripartite motif-containing 61
	H3069B11	0.0005	data not found	Mm.354103	Transcribed locus
	H3067A09	0.0005	Zdhhc13	Mm.279116	Zinc finger, DHHC domain containing 13
	H3152B03	0.0005	Grid2	Mm.425327	Glutamate receptor, ionotropic, delta 2
	H3144A01	0.0005	4930562C15Rik	Mm.325551	RIKEN cDNA 4930562C15 gene
	H553026	0.0005	Top3b	Mm.326089	Topoisomerase (DNA) III beta
	H3070B01	0.0005	Zbed3	Mm.3774	Zinc finger, BED domain containing 3
	H3133D07	0.0005	Tmtc4	Mm.271988	Transmembrane and tetratricopeptide repeat containing 4
	H3135G08	0.0005	AU015836	Mm.328014	Expressed sequence AU015836
	H3080G03	0.0005	Nlrp4f	Mm.315815	NLR family, pyrin domain containing 4F
	H3082H03	0.0005	Ascc3	Mm.222497	Activating signal cointegrator 1 complex subunit 3
	H3104H01	0.0005	C86187	Mm.22314	Expressed sequence C86187
	H3127E07	0.0005	Ctnna3	Mm.244348	Catenin (cadherin associated protein), alpha 3
	H3058B02	0.0005	Tpd52	Mm.371590	Tumor protein D52
	H3134D03	0.0005	Nmnat3	Mm.294082	Nicotinamide nucleotide adenylyltransferase 3
	H551381	0.0005	Osbpl8	Mm.220204	Oxysterol binding protein-like 8
‡	H636958	0.0006	EG626058	Mm.31556	Predicted gene, EG626058
	H3159F10	0.0006	Dhx40	Mm.260627	DEAH (Asp-Glu-Ala-His) box polypeptide 40
	H551688	0.0006	Prep	Mm.37294	Prolyl endopeptidase
	H3129D01	0.0006	data not found	Mm.401652	Transcribed locus, moderately similar to XP_925585.1 hypothetical protein XP_920492
	H3051D02	0.0006	Exo1	Mm.283046	Exonuclease 1
	H3064C07	0.0006	AI427122	Mm.11869	Expressed sequence AI427122
	H3128G07	0.0006	data not found	Mm.410520	Transcribed locus
	H3156G03	0.0006	4931419K03Rik	Mm.235443	RIKEN cDNA 4931419K03 gene
	H3072G05	0.0006	Elavl2	Mm.318042	ELAV (embryonic lethal, abnormal vision, Drosophila)-like 2 (Hu antigen B)
	H3059F01	0.0006	Uchl1	Mm.29807	Ubiquitin carboxy-terminal hydrolase L1
	H555534	0.0006	Fbxo21	Mm.21912	F-box protein 21

	NIA	ρ ≤	Common	Unigene	Name
‡	H3134E07	0.0007	data not found	Mm.300962	Adult male olfactory brain cDNA, RIKEN full-length enriched library, clone:6430591E23
	H3131C01	0.0007	Dsn1	Mm.25410	DSN1, MIND kinetochore complex component, homolog
	H585251	0.0007	Cpeb1	Mm.273122	Cytoplasmic polyadenylation element binding protein 1
	H3049G03	0.0007	D8Ertd362e	Mm.394064	DNA segment, Chr 8, ERATO Doi 362, expressed
	H720076	0.0007	data not found	Mm.361796	Adult male cecum cDNA, RIKEN full-length enriched library, clone:9130020F13
	H3139B12	0.0007	Spt1	Mm.1280	Salivary protein 1
	H3153H08	0.0007	Hs6st2	Mm.252561	Heparan sulfate 6-O-sulfotransferase 2
	H3076E02	0.0007	C330027C09Rik	Mm.24491	RIKEN cDNA C330027C09 gene
	H3158G04	0.0007	Stat3	Mm.249934	Signal transducer and activator of transcription 3
	H3050H08	0.0007	BC031748	Mm.391313	CDNA sequence BC031748
	H3101B05	0.0007	4930461P20Rik	Mm.297862	RIKEN cDNA 4930461P20 gene
	H3055D02	0.0007	Aldob	Mm.218862	Aldolase 2, B isoform
	H620813	0.0007	Rdx	Mm.245746	Radixin
	H3078C06	0.0008	9230115E21Rik	Mm.393649	RIKEN cDNA 9230115E21 gene
	H3120H01	0.0008	Prnd	Mm.180750	Prion protein dublet
	H3066B07	0.0008	Rfpl4	Mm.28764	Ret finger protein-like 4
	H3022D07	0.0008	1810030O07Rik	Mm.15974	RIKEN cDNA 1810030O07 gene
	H3078A01	0.0008	data not found	Mm.393605	8 cells embryo 8 cells cDNA, RIKEN full-length enriched library, clone:E860019P17
	H3061A05	0.0008	Zbtb10	Mm.103262	Zinc finger and BTB domain containing 10
	H3055G11	0.0008	fbxo21	Mm.410455	Transcribed locus
	H3071A09	0.0008	Spbc25	Mm.272969	Spindle pole body component 25 homolog
	H3139C03	0.0008	Cd9	Mm.210676	CD9 antigen
	H3096A08	0.0008	Paqr5	Mm.273267	Progestin and adipoQ receptor family member V
	H3140H01	0.0008	data not found	Mm.103226	Transcribed locus, weakly similar to XP_573813.1 similar to envelope protein
	H3075E03	0.0008	E330017A01Rik	Mm.26145	RIKEN cDNA E330017A01 gene
	H583647	0.0008	Kif14	Mm.384849	Kinesin family member 14
	H3110B11	0.0008	2900002G04Rik	Mm.160079	RIKEN cDNA 2900002G04 gene
	H3040F10	0.0008	Lmo7	Mm.393651	LIM domain only 7
	H3151F06	0.0008	Xdh	Mm.11223	Xanthine dehydrogenase
	H3040G07	0.0008	Anxa7	Mm.280231	Annexin A7
	H3068F02	0.0009	Spg3a	Mm.386814	Spastic paraplegia 3A homolog
	H3062A08	0.0009	data not found	Mm.394733	Transcribed locus
	H3059B02	0.0009	Rgs12	Mm.196208	Regulator of G-protein signaling 12
	H575895	0.0009	2010301N04Rik	Mm.31770	RIKEN cDNA 2010301N04 gene
	H3060B08	0.0009	2700059D21Rik	Mm.389532	RIKEN cDNA 2700059D21 gene
	H3025C02	0.0009	7420416P09Rik	Mm.311913	RIKEN cDNA 7420416P09 gene

	NIA	ρ ≤	Common	Unigene	Name
	H3060F01	0.0009	Xlr3b	Mm.379042	X-linked lymphocyte-regulated 3B
	H3058E02	0.0010	data not found	Mm.390234	Transcribed locus
	H552702	0.0010	Acot3	Mm.202331	Acyl-CoA thioesterase 3
	H3071A04	0.0010	data not found	Mm.410475	Transcribed locus
‡	H3138G05	0.0010	Zp2	Mm.6510	Zona pellucida glycoprotein 2
	H3133E07	0.0011	Gatm	Mm.29975	Glycine amidinotransferase (L-arginine:glycine amidinotransferase)
	H3052B10	0.0011	2210009G21Rik	Mm.330612	RIKEN cDNA 2210009G21 gene
	H521770	0.0011	Ctrc	Mm.45316	Chymotrypsin C (caldecrin)
	H3056F01	0.0011	Gdf9	Mm.9714	Growth differentiation factor 9
	H3105B01	0.0011	Sms	Mm.18652	Spermine synthase
	H3061A04	0.0011	6720468P15Rik	Mm.12411	RIKEN cDNA 6720468P15 gene
	H3047C05	0.0011	Ccdc123	Mm.133263	Coiled-coil domain containing 123
	H3137H03	0.0011	Mcm2	Mm.16711	Minichromosome maintenance deficient 2 mitotin+E129
	H3152D10	0.0011	Mat2b	Mm.293771	Methionine adenosyltransferase II, beta
	H3085C10	0.0012	Zfp238	Mm.330700	Zinc finger protein 238
	H3051E04	0.0012	data not found	Mm.410451	Transcribed locus
	H3149H09	0.0012	1700006H03Rik	Mm.325325	RIKEN cDNA 1700006H03 gene
	H3123E01	0.0012	Syce2	Mm.229128	Synaptonemal complex central element protein 2
	H3130G08	0.0012	Prdm4	Mm.25307	PR domain containing 4
	H3135D05	0.0013	Col9a3	Mm.141312	Procollagen, type IX, alpha 3
	H3142A06	0.0013	Mphosph6	Mm.181836	M phase phosphoprotein 6
	H3060B07	0.0013	Ndg1	Mm.26006	Nur77 downstream gene 1
	H3001D07	0.0013	B230333C21Rik	Mm.116704	RIKEN cDNA B230333C21 gene
	H3099F09	0.0013	Dppa5	Mm.139314	Developmental pluripotency associated 5
	H3079D05	0.0014	Suv420h2	Mm.247844	Suppressor of variegation 4-20 homolog 2
	H3033C02	0.0014	Plk4	Mm.3794	Polo-like kinase 4
	H3051G02	0.0014	Cpeb3	Mm.391176	Cytoplasmic polyadenylation element binding protein 3
	H3121G07	0.0014	Rif1	Mm.254530	Rap1 interacting factor 1 homolog
	H634968	0.0014	Slc27a2	Mm.290044	Solute carrier family 27 (fatty acid transporter), member 2
	H3074F02	0.0014	Atp11b	Mm.239416	ATPase, Class VI, type 11B
	H3063H01	0.0014	Tbc1d15	Mm.432177	TBC1 domain family, member 15
	H3058G08	0.0015	Nexn	Mm.200188	Nexilin
	H3102C08	0.0015	Zp3	Mm.1381	Zona pellucida glycoprotein 3
	H596497	0.0015	data not found	Mm.260106	Transcribed locus
	H636897	0.0015	D030056L22Rik	Mm.28101	RIKEN cDNA D030056L22 gene
	H3148A01	0.0016	Sdc2	Mm.234266	Syndecan 2

	NIA	ρ ≤	Common	Unigene	Name
	H3110C01	0.0016	Epb4.1l4b	Mm.28217	Erythrocyte protein band 4.1-like 4b
	H582411	0.0016	Pigf	Mm.219685	Phosphatidylinositol glycan anchor biosynthesis, class F
	H598406	0.0016	Rock1	Mm.6710	Rho-associated coiled-coil containing protein kinase 1
	H3059C11	0.0016	data not found	Mm.366840	Transcribed locus, weakly similar to XP_577160.1 similar to LRRGT00088
	H3010B02	0.0016	Mertk	Mm.239655	C-mer proto-oncogene tyrosine kinase
	H3103G12	0.0016	Tle4	Mm.103638	Transducin-like enhancer of split 4
	H3068E02	0.0016	Zcrb1	Mm.293181	Zinc finger CCHC-type and RNA binding motif 1
	H3072H12	0.0016	E2f5	Mm.153415	E2F transcription factor 5
	H3084A08	0.0016	4921517N04Rik	Mm.276415	RIKEN cDNA 4921517N04 gene
	H3043D08	0.0016	C79562	Mm.318680	Expressed sequence C79562
	H3063H02	0.0016	Wwc2	Mm.235074	WW, C2 and coiled-coil domain containing 2
	H636540	0.0016	Jag1	Mm.22398	Jagged 1
	H3083D07	0.0017	D5Ertd615e	Mm.394707	DNA segment, Chr 5, ERATO Doi 615, expressed
	H3159E02	0.0017	Marveld2	Mm.25300	MARVEL (membrane-associating) domain containing 2
	H3078D03	0.0017	Ndrg4	Mm.29846	N-myc downstream regulated gene 4
	H3136E03	0.0017	Wdr36	Mm.38816	WD repeat domain 36
	H3159D01	0.0017	Wee1	Mm.287173	Wee 1 homolog (S. pombe)
	H3027E04	0.0017	Bcat1	Mm.4606	Branched chain aminotransferase 1, cytosolic
	H3029B11	0.0018	Hmga1	Mm.4438	High mobility group AT-hook 1
	H3068B03	0.0018	AU023525	Mm.394898	Expressed sequence AU023525
	H619810	0.0018	Lancl2	Mm.274904	LanC (bacterial lantibiotic synthetase component C)-like 2
	H3107H03	0.0018	C330008K14Rik	Mm.30649	RIKEN cDNA C330008K14 gene
	H3055H09	0.0018	D15Ertd466e	Mm.387118	DNA segment, Chr 15, ERATO Doi 466, expressed
	H3093G05	0.0018	C130092O11Rik	Mm.376946	RIKEN cDNA C130092O11 gene
	H3158A11	0.0018	Kif16b	Mm.251934	Kinesin family member 16B
	H315658	0.0019	Olah	Mm.13808	Oleoyl-ACP hydrolase
	H3060A01	0.0019	Fam18b	Mm.9806	Family with sequence similarity 18, member B
	H3091C02	0.0019	Rex2	Mm.327224	Reduced expression 2
	H3113G05	0.0019	Ypel5	Mm.277533	Yippee-like 5
	H3052A09	0.0019	E330034G19Rik	Mm.2077	RIKEN cDNA E330034G19 gene
	H3035G08	0.0019	Hira	Mm.15694	Histone cell cycle regulation defective homolog A+E183
	H3046B08	0.0019	Anxa11	Mm.294083	Annexin A11
	H3071C04	0.0020	Camta1	Mm.318846	Calmodulin binding transcription activator 1
	H3083G08	0.0020	Rbm35a	Mm.387719	RNA binding motif protein 35A
	H3086C04	0.0021	4933426K21Rik	Mm.277939	RIKEN cDNA 4933426K21 gene
	H3033B11	0.0021	Serpinb6c	Mm.272188	Serine (or cysteine) peptidase inhibitor, clade B, member 6c

	NIA	ρ ≤	Common	Unigene	Name
	H3119G10	0.0021	6330577E15Rik	Mm.181610	RIKEN cDNA 6330577E15 gene
	H3024G07	0.0021	Mycl1	Mm.1055	V-myc myelocytomatosis viral oncogene homolog 1, lung carcinoma derived+E364
	H651495	0.0021	Tmem81	Mm.34611	Transmembrane protein 81
	H3139B09	0.0021	E130308A19Rik	Mm.45688	RIKEN cDNA E130308A19 gene
	H3144G07	0.0021	Ovol1	Mm.280225	OVO homolog-like 1
	H3135G03	0.0021	Serpinb6a	Mm.252210	Serine (or cysteine) peptidase inhibitor, clade B, member 6a
	H3054G01	0.0022	data not found	Mm.394349	Transcribed locus
	H3084E01	0.0023	Itga2b	Mm.311110	Integrin alpha 2b
	H3100F07	0.0023	Tacc3	Mm.379024	Transforming, acidic coiled-coil containing protein 3
	H3065B08	0.0024	Rnf141	Mm.96867	Ring finger protein 141
	H3025A04	0.0024	1110007M04Rik	Mm.204831	RIKEN cDNA 1110007M04 gene
	H3079H08	0.0024	Gpd1l	Mm.38198	Glycerol-3-phosphate dehydrogenase 1-like
	H3147G09	0.0024	data not found	Mm.315593	Nonagouti
	H367569	0.0025	Otud5	Mm.142827	OTU domain containing 5
	H3065C08	0.0025	Ppp3cb	Mm.274432	Protein phosphatase 3, catalytic subunit, beta isoform
	H3117C02	0.0025	Nusap1	Mm.290015	Nucleolar and spindle associated protein 1
	H3011D10	0.0026	Lcp1	Mm.153911	Lymphocyte cytosolic protein 1
	H3090E01	0.0026	Wdr5	Mm.28265	WD repeat domain 5
	H597152	0.0026	Nat12	Mm.275688	N-acetyltransferase 12
	H3110E03	0.0026	Pgm2l1	Mm.57225	Phosphoglucomutase 2-like 1
	H3057D08	0.0026	Shcbp1	Mm.37801	Shc SH2-domain binding protein 1
	H3016B05	0.0027	Adss	Mm.338021	Adenylosuccinate synthetase, non muscle
	H477236	0.0027	Wasl	Mm.1574	Wiskott-Aldrich syndrome-like
	H3086H01	0.0027	Necap1	Mm.288114	NECAP endocytosis associated 1
	H3011B11	0.0028	Tex2	Mm.102407	Testis expressed gene 2
	H3154E01	0.0028	Rad50	Mm.4888	RAD50 homolog
	H597752	0.0028	Herc3	Mm.191892	Hect domain and RLD 3
	H3146F03	0.0029	5530401J07Rik	Mm.392614	RIKEN cDNA 5530401J07 gene
	H3146A01	0.0029	Rnf7	Mm.28235	Ring finger protein 7
	H3143G02	0.0030	Mrpl24	Mm.196635	Mitochondrial ribosomal protein L24
	H3046F10	0.0031	Rnf8	Mm.305994	Ring finger protein 8
	H3088C10	0.0031	Cks2	Mm.222228	CDC28 protein kinase regulatory subunit 2
	H3149F01	0.0031	Wdr47	Mm.285968	WD repeat domain 47
	H3078F01	0.0032	Trim75	Mm.334557	Tripartite motif-containing 75
	H3068B07	0.0032	Zc3h6	Mm.26377	Zinc finger CCCH type containing 6
	H3093C05	0.0032	Glra4	Mm.215168	Glycine receptor, alpha 4 subunit

	NIA	ρ ≤	Common	Unigene	Name
	H3100D02	0.0032	Tram1	Mm.28765	Translocating chain-associating membrane protein 1
	H3092H10	0.0034	Gyg	Mm.6375	Glycogenin
	H3146G01	0.0034	Tfdp1	Mm.925	Transcription factor Dp 1
	H3101D01	0.0034	Cep70	Mm.288739	Centrosomal protein 70
	H617607	0.0034	E330009J07Rik	Mm.333006	RIKEN cDNA E330009J07 gene
	H3058B06	0.0035	C87487	Mm.394376	Expressed sequence C87487
	H3083E11	0.0035	1700022F17Rik	Mm.310284	RIKEN cDNA 1700022F17 gene
	H3053E01	0.0035	Sart2	Mm.34557	Squamous cell carcinoma antigen recognized by T cells 2
	H3145B08	0.0035	Ung2	Mm.25457	Uracil DNA glycosylase 2
	H518698	0.0035	Dcun1d1	Mm.379305	DCUN1D1 DCN1, defective in cullin neddylation 1, domain containing 1
	H3085H07	0.0035	Vps26a	Mm.260703	Vacuolar protein sorting 26 homolog A
	H3081G02	0.0035	Tmem41b	Mm.43212	Transmembrane protein 41B
	H3025B04	0.0035	Actr10	Mm.29317	ARP10 actin-related protein 10 homolog
	H3145A06	0.0035	Slc30a3	Mm.1396	Solute carrier family 30 (zinc transporter), member 3
	H3100C02	0.0036	Klhl8	Mm.179871	Kelch-like 8
	H3033E08	0.0036	Nedd4l	Mm.98668	Neural precursor cell expressed, developmentally down-regulated gene 4-like
	H3070F07	0.0037	Lpp	Mm.391933	LIM domain containing preferred translocation partner in lipoma
	H3102E07	0.0037	Nlrp5	Mm.333653	NLR family, pyrin domain containing 5
	H3092H04	0.0038	Nhej1	Mm.335937	Nonhomologous end-joining factor 1
	H3087E08	0.0038	A030007L17Rik	Mm.294708	RIKEN cDNA A030007L17 gene
	H3028H03	0.0038	Psip1	Mm.271985	PC4 and SFRS1 interacting protein 1
	H3083B01	0.0038	Ube4b	Mm.405832	Ubiquitination factor E4B, UFD2 homolog+E156
	H3090G01	0.0038	1110067D22Rik	Mm.76694	RIKEN cDNA 1110067D22 gene
	H3116H07	0.0038	A530047J11Rik	Mm.34175	RIKEN cDNA A530047J11 gene
	H3121H04	0.0038	Pes1	Mm.28659	Pescadillo homolog 1, containing BRCT domain
	H3061E11	0.0039	Pfdn4	Mm.28808	Prefoldin 4
	H402976	0.0039	Tbc1d1	Mm.286353	TBC1 domain family, member 1
	H3109A01	0.0039	Phf8	Mm.17156	PHD finger protein 8
	H3085C12	0.0039	1810015C04Rik	Mm.25311	RIKEN cDNA 1810015C04 gene
	H3152H12	0.0039	Pard3	Mm.299254	Par-3 (partitioning defective 3) homolog
	H3085E01	0.0039	Mier3	Mm.31012	Mesoderm induction early response 1, family member 3
	H3054H08	0.0040	Hddc3	Mm.21171	HD domain containing 3
	H3154E08	0.0040	Pdzk1	Mm.28015	PDZ domain containing 1
	H3083A03	0.0042	1700029F09Rik	Mm.348017	RIKEN cDNA 1700029F09 gene
	H3137D12	0.0042	Vkorc1	Mm.29703	Vitamin K epoxide reductase complex, subunit 1
	H3084G04	0.0042	Smg6	Mm.288460	Smg-6 homolog, nonsense mediated mRNA decay factor

	NIA	ρ ≤	Common	Unigene	Name
	H3132C07	0.0042	Chfr	Mm.30264	Checkpoint with forkhead and ring finger domains
	H3136C07	0.0042	Bnip3	Mm.378890	BCL2/adenovirus E1B interacting protein 1, NIP3
	H3123C03	0.0042	Clock	Mm.3552	Circadian locomoter output cycles kaput
	H3112D07	0.0043	Dusp14	Mm.240885	Dual specificity phosphatase 14
	H3060D03	0.0044	Pla2g4c	Mm.223639	Phospholipase A2, group IVC (cytosolic, calcium-independent)
	H3083A09	0.0044	Rbm26	Mm.291542	RNA binding motif protein 26
	H3135G06	0.0045	Aof1	Mm.31259	Amine oxidase, flavin containing 1
	H3033D02	0.0045	Phf12	Mm.274329	PHD finger protein 12
	H3024A07	0.0045	Ube2c	Mm.89830	Ubiquitin-conjugating enzyme E2C
	H3113F01	0.0045	Nat13	Mm.278726	N-acetyltransferase 13
	H3080A01	0.0046	Ccdc112	Mm.329416	Coiled-coil domain containing 112
	H400526	0.0046	1110018M03Rik	Mm.291809	RIKEN cDNA 1110018M03 gene
	H3039H01	0.0046	Cnn2	Mm.157770	Calponin 2
	H3107A05	0.0047	Baiap2l1	Mm.18814	BAI1-associated protein 2-like 1
	H3066G07	0.0047	Thrap6	Mm.195562	Thyroid hormone receptor associated protein 6
	H598317	0.0048	Itpr1	Mm.227912	Inositol 1,4,5-triphosphate receptor 1
‡	H3075A04	0.0048	Padi6	Mm.271661	Peptidyl arginine deiminase, type VI
	H3146E01	0.0048	Rpa2	Mm.2870	Replication protein A2
	H3032G01	0.0048	data not found	Mm.392817	Transcribed locus, weakly similar to NP_983802.1
	H3090G02	0.0049	Csde1	Mm.277713	Cold shock domain containing E1, RNA binding
	H3058H01	0.0050	Oas1c	Mm.43230	2'-5' oligoadenylate synthetase 1C
	H3148D05	0.0050	Dcn	Mm.56769	Decorin
	H3094A03	0.0050	Epn2	Mm.139695	Epsin 2
	H3049F01	0.0050	Fancm	Mm.374847	Fanconi anemia, complementation group M
	H3016G07	0.0051	Mllt4	Mm.59167	Myeloid/lymphoid or mixed lineage-leukemia translocation to 4 homolog
	H3058G06	0.0052	Trpm7	Mm.244705	Transient receptor potential cation channel, subfamily M, member 7
	H3053H07	0.0052	Eef2k	Mm.25997	Eukaryotic elongation factor-2 kinase
	H3082G08	0.0052	Myo10	Mm.60590	Myosin X
	H3116D03	0.0052	Rad51	Mm.434303	RAD51 homolog+E199
	H3004B11	0.0052	Sh3bgrl2	Mm.100125	SH3 domain binding glutamic acid-rich protein like 2
	H3004E10	0.0052	Suclg2	Mm.371585	Succinate-Coenzyme A ligase, GDP-forming, beta subunit
	H3008G08	0.0052	Ttc33	Mm.254979	Tetratricopeptide repeat domain 33
	H3101C02	0.0052	Ube2t	Mm.284587	Ubiquitin-conjugating enzyme E2T (putative)
‡	H3028H01	0.0052	Pou5f1	Mm.17031	POU domain, class 5, transcription factor 1
	H3137A01	0.0053	Tlk2	Mm.126976	Tousled-like kinase 2 (Arabidopsis)
	H3114E05	0.0055	2410127L17Rik	Mm.29592	RIKEN cDNA 2410127L17 gene

	NIA	ρ ≤	Common	Unigene	Name
	H3108E02	0.0055	Mapk6	Mm.18856	Mitogen-activated protein kinase 6
	H3159C02	0.0055	Tle6	Mm.206764	Transducin-like enhancer of split 6
	H3151C02	0.0056	Phc1	Mm.6822	Polyhomeotic-like 1
	H3051H08	0.0058	Btg4	Mm.431317	B-cell translocation gene 4
	H552704	0.0058	Tor3a	Mm.206737	Torsin family 3, member A
	H3092B11	0.0058	Ralbp1	Mm.17009	RalA binding protein 1
	H602659	0.0058	1200011O22Rik	Mm.425292	RIKEN cDNA 1200011O22 gene
	H3074B09	0.0059	Dcp1a	Mm.28733	Decapping enzyme
	H3120D12	0.0059	Zfp313	Mm.22225	Zinc finger protein 313
	H3056E12	0.0060	data not found	Mm.400434	Transcribed locus
	H3014D08	0.0061	Igf2bp2	Mm.294740	Insulin-like growth factor 2 mRNA binding protein 2
	H3113E01	0.0061	Cdc23	Mm.196638	CDC23
	H367250	0.0061	Sohlh2	Mm.25514	Spermatogenesis and oogenesis specific basic helix-loop-helix 2
	H403917	0.0061	LOC329427	Mm.434022	Hypothetical protein LOC329427
	H3079C07	0.0061	Rhpn2	Mm.286600	Rhophilin, Rho GTPase binding protein 2
	H583397	0.0061	Rpa3	Mm.29073	Replication protein A3
	H3076H06	0.0063	Ak3l1	Mm.42040	Adenylate kinase 3 alpha-like 1
	H3027C03	0.0063	Cops3	Mm.40	COP9 (constitutive photomorphogenic) homolog, subunit 3
	H3089E03	0.0064	Rrm2b	Mm.24738	Ribonucleotide reductase M2 B (TP53 inducible)
	H3083F03	0.0064	Tmem87b	Mm.259688	Transmembrane protein 87B
	H3126D02	0.0064	Tmem57	Mm.99793	Transmembrane protein 57
	H3074G01	0.0065	Dnaja3	Mm.325524	DnaJ (Hsp40) homolog, subfamily A, member 3
	H3152H02	0.0065	Tbc1d20	Mm.288733	TBC1 domain family, member 20
	H551410	0.0067	Mcpt4	Mm.266575	Mast cell protease 4
	H3055A01	0.0067	data not found	Mm.432968	Transcribed locus
	H3031D01	0.0068	Orc1l	Mm.294154	Origin recognition complex, subunit 1-like
	H3042B08	0.0069	Chek1	Mm.16753	Checkpoint kinase 1 homolog
	H3133C04	0.0070	Ptk2	Mm.254494	PTK2 protein tyrosine kinase 2
	H480618	0.0070	Crsp2	Mm.17616	Cofactor required for Sp1 transcriptional activation, subunit 2
	H3099D10	0.0071	BC049349	Mm.334607	CDNA sequence BC049349
	H3113G01	0.0071	D12Ertd551e	Mm.273755	DNA segment, Chr 12, ERATO Doi 551, expressed
	H3103B07	0.0071	Bnip3l	Mm.29820	BCL2/adenovirus E1B interacting protein 3-like
	H3035F04	0.0071	2610019F03Rik	Mm.5727	RIKEN cDNA 2610019F03 gene
	H598513	0.0071	Ero1lb	Mm.358706	ERO1-like beta
	H466415	0.0072	Mid1ip1	Mm.29429	Mid1 interacting protein 1
	H338143	0.0072	Gdpd1	Mm.281887	Glycerophosphodiester phosphodiesterase domain containing 1

	NIA	ρ ≤	Common	Unigene	Name
	H3131G01	0.0072	0610007P06Rik	Mm.28679	RIKEN cDNA 0610007P06 gene
	H3096F11	0.0072	Chst10	Mm.260054	Carbohydrate sulfotransferase 10
	H3003C02	0.0072	Cldnd1	Mm.29482	Claudin domain containing 1
	H3109D09	0.0074	Ssx2ip	Mm.200783	Synovial sarcoma, X breakpoint 2 interacting protein
	H3093A02	0.0074	Tmem16e	Mm.254431	Transmembrane protein 16E
	H3009B03	0.0074	Dynll1	Mm.256858	Dynein light chain LC8-type 1
	H3101E01	0.0074	Ube2q2	Mm.207894	Ubiquitin-conjugating enzyme E2Q (putative) 2
	H3159G02	0.0074	Rac1	Mm.292510	RAS-related C3 botulinum substrate 1
	H3015C11	0.0075	Armc1	Mm.434309	Armadillo repeat containing 1
	H3053D09	0.0078	Ccdc117	Mm.210403	Coiled-coil domain containing 117
	H3088B08	0.0078	Fbxo8	Mm.251174	F-box protein 8
	H3045D07	0.0078	Naalad2	Mm.7060	N-acetylated alpha-linked acidic dipeptidase 2
	H4920352	0.0078	Parp1	Mm.277779	Poly (ADP-ribose) polymerase family, member 1
	H3085A11	0.0078	Pbef1	Mm.202727	Pre-B-cell colony-enhancing factor 1
	H551137	0.0078	Vrk3	Mm.28816	Vaccinia related kinase 3
	H3131C08	0.0079	D030074E01Rik	Mm.273769	RIKEN cDNA D030074E01 gene
	H3085D05	0.0079	Mtap7	Mm.20928	Microtubule-associated protein 7
	H3006A01	0.0080	Arfgef1	Mm.229141	ADP-ribosylation factor guanine nucleotide-exchange factor 1(brefeldin A-inhibited)
	H3051B07	0.0080	Spin	Mm.188432	Spindlin
	H3024D07	0.0080	Stip1	Mm.258633	Stress-induced phosphoprotein 1
	H3146D02	0.0080	Tmem64	Mm.394111	Transmembrane protein 64
	H3073H11	0.0081	Fdft1	Mm.434391	Farnesyl diphosphate farnesyl transferase 1
	H3134G01	0.0081	Fundc1	Mm.41558	FUN14 domain containing 1
	H3131E12	0.0081	Tdrd5	Mm.252800	Tudor domain containing 5
	H3026E12	0.0081	Uhrf1	Mm.42196	Ubiquitin-like, containing PHD and RING finger domains, 1
	H3069B07	0.0082	Aebp2	Mm.86453	AE binding protein 2
	H3074E07	0.0082	Atpbd1c	Mm.266328	ATP binding domain 1 family, member C
	H3158B08	0.0083	Prkab2	Mm.31175	Protein kinase, AMP-activated, beta 2 non-catalytic subunit
	H3005D01	0.0083	Dpp4	Mm.1151	Dipeptidylpeptidase 4
	H3060G09	0.0085	data not found	Mm.175989	Membrane-associated ring finger (C3HC4) 5
	H3043F01	0.0085	Fhod3	Mm.329322	Formin homology 2 domain containing 3
	H3013G10	0.0085	Birc5	Mm.8552	Baculoviral IAP repeat-containing 5
	H3060H03	0.0086	Tcl1	Mm.18154	T-cell lymphoma breakpoint 1
	H3080B07	0.0086	Lix1	Mm.268018	Limb expression 1 homolog+E276
	H3081B07	0.0087	Bzw1	Mm.21848	Basic leucine zipper and W2 domains 1
	H3027G07	0.0087	Cdv3	Mm.261025	Carnitine deficiency-associated gene expressed in ventricle 3

	NIA	ρ ≤	Common	Unigene	Name
	H3119A01	0.0087	Drr1	Mm.218676	Developmentally regulated repeat element-containing transcript 1
	H3149G05	0.0087	Ccni	Mm.250419	Cyclin I
	H670423	0.0088	Klhl21	Mm.22306	Kelch-like 21
	H3138D03	0.0089	Tbc1d8	Mm.387293	TBC1 domain family, member 8
	H404508	0.0091	Zfp57	Mm.305561	Zinc finger protein 57
	H3138D04	0.0091	Cenpq	Mm.9870	Centromere protein Q
	H3068B08	0.0093	Tmem30b	Mm.251196	Transmembrane protein 30B
	H3147C08	0.0095	data not found	Mm.401682	Transcribed locus
	H3071C06	0.0095	Gbas	Mm.12468	Glioblastoma amplified sequence
	H3042E07	0.0097	Spata13	Mm.149776	Spermatogenesis associated 13
	H3023G07	0.0098	Ctps	Mm.1815	Cytidine 5'-triphosphate synthase
	H3126C01	0.0099	Rab5a	Mm.300065	RAB5A, member RAS oncogene family
	H3135F02	0.0100	Exoc4	Mm.265512	Exocyst complex component 4
	H3128G04	0.0100	Ldhb	Mm.9745	Lactate dehydrogenase B
	H3053G09	0.0100	Cep55	Mm.9916	Centrosomal protein 55
	H3062G11	0.0102	Dbf4	Mm.292470	DBF4 homolog
	H3127D01	0.0102	Snrpb	Mm.88216	Small nuclear ribonucleoprotein B
	H3015G01	0.0102	Ube2a	Mm.395649	Ubiquitin-conjugating enzyme E2A, RAD6 homolog
	H3025H01	0.0103	H2-Ke2	Mm.2948	H2-K region expressed gene 2
	H3109H03	0.0104	Ell2	Mm.21288	Elongation factor RNA polymerase II 2
	H3042D05	0.0104	Skap2	Mm.221479	Src family associated phosphoprotein 2
	H3120B01	0.0105	Coro1c	Mm.260158	Coronin, actin binding protein 1C
	H442438	0.0105	Gvb1	Mm.293266	GA repeat binding protein, beta 1
	H4976288	0.0105	Il1r1	Mm.896	Interleukin 1 receptor, type I
	H3159C05	0.0107	Rab3d	Mm.260157	RAB3D, member RAS oncogene family
	H3013E01	0.0108	Clic4	Mm.257765	Chloride intracellular channel 4 (mitochondrial)
	H3134G04	0.0108	Ddx4	Mm.12818	DEAD (Asp-Glu-Ala-Asp) box polypeptide 4
	H3134G02	0.0108	Rnf12	Mm.435574	Ring finger protein 12
	H3109E02	0.0110	Anks1	Mm.32556	Ankyrin repeat and SAM domain containing 1
	H3055G02	0.0110	Ctsc	Mm.322945	Cathepsin C
	H3029G02	0.0110	Nfxl1	Mm.187453	Nuclear transcription factor, X-box binding-like 1
	H3093F04	0.0111	Ddx20	Mm.272826	DEAD (Asp-Glu-Ala-Asp) box polypeptide 20
	H3006C01	0.0111	Nasp	Mm.257181	Nuclear autoantigenic sperm protein (histone-binding)
	H615180	0.0112	Gpr108	Mm.28468	G protein-coupled receptor 108
	H3149D07	0.0112	Ncoa5	Mm.233080	Nuclear receptor coactivator 5
	H3022E02	0.0112	Nucks1	Mm.246869	Nuclear casein kinase and cyclin-dependent kinase substrate 1

	NIA	ρ ≤	Common	Unigene	Name
	H3020E01	0.0113	Hsp110	Mm.270681	Heat shock protein 110
	H3332261	0.0113	Jak1	Mm.289657	Janus kinase 1
	H354417	0.0114	AL033314	Mm.391917	Expressed sequence AL033314
	H3031E03	0.0114	Ddx10	Mm.133560	DEAD (Asp-Glu-Ala-Asp) box polypeptide 10
	H3134D06	0.0114	Grpel2	Mm.269657	GrpE-like 2, mitochondrial
	H3043H10	0.0114	Vprbp	Mm.422783	Vpr (HIV-1) binding protein
	H475662	0.0115	Pdcd7	Mm.29193	Programmed cell death protein 7
	H3079D04	0.0116	Celsr1	Mm.22680	Cadherin EGF LAG seven-pass G-type receptor 1
	H3022G05	0.0116	Chmp5	Mm.212763	Chromatin modifying protein 5
	H3043B01	0.0117	Atad2b	Mm.210966	ATPase family, AAA domain containing 2B
	H3043A09	0.0119	Kpna2	Mm.12508	Karyopherin (importin) alpha 2
	H3128G06	0.0120	Alkbh5	Mm.262056	AlkB, alkylation repair homolog 5
	H539100	0.0121	Ipo8	Mm.260105	Importin 8
	H597483	0.0122	data not found	Mm.270259	Septin 7
	H3070H08	0.0122	data not found	Mm.351228	Transcribed locus
	H3151D07	0.0125	Abhd10	Mm.247453	Abhydrolase domain containing 10
	H3059A05	0.0125	Mad2l1	Mm.290830	MAD2 (mitotic arrest deficient, homolog)-like 1
	H3029F02	0.0125	Nsf	Mm.260117	N-ethylmaleimide sensitive fusion protein
	H329436	0.0125	Thbd	Mm.24096	Thrombomodulin
	H3101C03	0.0125	Ube2g1	Mm.340315	Ubiquitin-conjugating enzyme E2G 1
	H3146D11	0.0126	Cd8b1	Mm.153963	CD8 antigen, beta chain 1
	H3021F09	0.0126	Smc2	Mm.2999	Structural maintenance of chromosomes 2
	H3052G01	0.0128	Mdm4	Mm.426531	Transformed mouse 3T3 cell double minute 4
	H3106D07	0.0129	Cdc42	Mm.1022	Cell division cycle 42 homolog
	H3034B07	0.0130	Adamts1	Mm.1421	A disintegrin-like and metallopeptidase (reprolysin type) with thrombospondin type 1 motif, 1
	H3111B10	0.0130	Ap3b2	Mm.322894	Adaptor-related protein complex 3, beta 2 subunit
	H3144F10	0.0134	8030402F09Rik	Mm.258969	RIKEN cDNA 8030402F09 gene
‡	H582767	0.0134	Vbp1	Mm.8294	Von Hippel-Lindau binding protein 1
	H3011H07	0.0135	Arcn1	Mm.371682	Archain 1
	H3142C11	0.0135	Bpgm	Mm.282863	2,3-bisphosphoglycerate mutase
	H3107H01	0.0135	Jam3	Mm.28770	Junction adhesion molecule 3
	H3085G07	0.0135	Tgoln1	Mm.246563	Trans-golgi network protein
	H3081D01	0.0136	data not found	Mm.26250	Transcribed locus
	H641403	0.0136	Elp4	Mm.33870	Elongation protein 4 homolog
	H421434	0.0136	Whsc2	Mm.332320	Wolf-Hirschhorn syndrome candidate 2
	H3038C09	0.0138	Prpsap2	Mm.27703	Phosphoribosyl pyrophosphate synthetase-associated protein 2

	NIA	ρ ≤	Common	Unigene	Name
	H3023E01	0.0138	Skp1a	Mm.42944	S-phase kinase-associated protein 1A
	H386370	0.0139	Hnrpa1	Mm.237064	Heterogeneous nuclear ribonucleoprotein A1
	H3152C01	0.0139	Rqcd1	Mm.291708	Rcd1 (required for cell differentiation) homolog 1
‡	H519629	0.0140	Rbpms2	Mm.29148	RNA binding protein with multiple splicing 2
	H3056A07	0.0141	Nubp1	Mm.29037	Nucleotide binding protein 1
	H3151C11	0.0141	Sec22a	Mm.259164	SEC22 vesicle trafficking protein-like A+E446
	H3137D07	0.0142	Abce1	Mm.5831	ATP-binding cassette, sub-family E (OABP), member 1
	H3081G04	0.0142	Rfk	Mm.7013	Riboflavin kinase
	H3135F10	0.0142	Trim71	Mm.17857	Tripartite motif-containing 71
	H3149C09	0.0143	Gprasp1	Mm.271980	G protein-coupled receptor associated sorting protein 1
	H3070F06	0.0143	Ipmk	Mm.245867	Inositol polyphosphate multikinase
	H3037G09	0.0144	Dnajc11	Mm.21353	DnaJ (Hsp40) homolog, subfamily C, member 11
	H3108C01	0.0145	4930444A02Rik	Mm.17631	RIKEN cDNA 4930444A02 gene
	H3142H09	0.0145	Epb4.1l2	Mm.306026	Erythrocyte protein band 4.1-like 2
	H3102B01	0.0145	Ube3a	Mm.9002	Ubiquitin protein ligase E3A
	H3059D06	0.0145	Zcchc8	Mm.279427	Zinc finger, CCHC domain containing 8
	H3131A02	0.0146	Sfpq	Mm.257276	Splicing factor proline/glutamine rich (polypyrimidine tract binding protein associated)
	H3140D08	0.0147	Fkbp1a	Mm.278458	FK506 binding protein 1a
	H3071A02	0.0147	Srpk1	Mm.15252	Serine/arginine-rich protein specific kinase 1
	H3063D04	0.0148	2010107G23Rik	Mm.294126	RIKEN cDNA 2010107G23 gene
	H3153D08	0.0149	Zfhx1a	Mm.3929	Zinc finger homeobox 1a
	H3007E09	0.0150	2610101N10Rik	Mm.292742	RIKEN cDNA 2610101N10 gene
	H3133G01	0.0150	Oxr1	Mm.254267	Oxidation resistance 1
	H3038E03	0.0150	Pawr	Mm.391419	PRKC, apoptosis, WT1, regulator
	H3070B02	0.0150	Vdp	Mm.15868	Vesicle docking protein
	H3151H01	0.0150	Vps41	Mm.27389	Vacuolar protein sorting 41+E589
	H617672	0.0151	1110005A23Rik	Mm.351579	RIKEN cDNA 1110005A23 gene
	H3061G08	0.0151	AU041783	Mm.226284	Expressed sequence AU041783
	H3071B07	0.0151	Mtus1	Mm.149438	Mitochondrial tumor suppressor 1
	H3051A08	0.0152	Nploc4	Mm.309520	Nuclear protein localization 4 homolog+E380
	H617676	0.0152	Tlr1	Mm.273024	Toll-like receptor 1
	H3096D07	0.0154	Nr2f1	Mm.14297	Nuclear receptor subfamily 2, group F, member 1
	H3024D01	0.0154	Tubb2c	Mm.227260	Tubulin, beta 2c
	H3094E02	0.0155	Ptch1	Mm.228798	Patched homolog 1
	H3095B01	0.0158	D430039N05Rik	Mm.330351	RIKEN cDNA D430039N05 gene
	H634745	0.0158	Rbm12	Mm.27660	RNA binding motif protein 12

	NIA	ρ ≤	Common	Unigene	Name
	H3098E02	0.0158	Vps54	Mm.170103	Vacuolar protein sorting 54 (yeast)
	H3031H01	0.0160	Agpat5	Mm.24117	1-acylglycerol-3-phosphate O-acyltransferase 5 (lysophosphatidic acid acyltransferase, epsilon)
	H3058H08	0.0160	Pros1	Mm.127156	Protein S (alpha)
	H3113E08	0.0161	Cops6	Mm.3981	COP9 (constitutive photomorphogenic) homolog, subunit 6
	H3151G08	0.0161	Ppig	Mm.11815	Peptidyl-prolyl isomerase G (cyclophilin G)
	H3113E07	0.0161	Rras2	Mm.276572	Related RAS viral (r-ras) oncogene homolog 2
	H3026B01	0.0161	Tbp	Mm.244820	TATA box binding protein
	H3069G10	0.0163	Dpf3	Mm.151308	D4, zinc and double PHD fingers, family 3
	H3073E01	0.0164	AU024076	Mm.326336	Expressed sequence AU024076
	H3097F11	0.0164	Mbl2	Mm.30045	Mannose binding lectin (C)
	H3159B07	0.0165	2900057K09Rik	Mm.323087	RIKEN cDNA 2900057K09 gene
	H400490	0.0166	Ube2n	Mm.434498	Ubiquitin-conjugating enzyme E2N
	H3087H01	0.0167	data not found	Mm.380982	Transcribed locus
	H3085B01	0.0168	Csnk1a1	Mm.26908	Casein kinase 1, alpha 1
	H618102	0.0168	Gimap4	Mm.333048	GTPase, IMAP family member 4
	H3029C08	0.0168	Paxip1	Mm.277190	PAX interacting (with transcription-activation domain) protein 1
	H621092	0.0169	D11Wsu47e	Mm.239566	DNA segment, Chr 11, Wayne State University 47, expressed
	H3013D03	0.0169	Rab10	Mm.378993	RAB10, member RAS oncogene family
	H3058D08	0.0170	Myh10	Mm.218233	Myosin, heavy polypeptide 10, non-muscle
	H3088C02	0.0171	Cd55	Mm.101591	CD55 antigen
	H3063D10	0.0172	Gbe1	Mm.396102	Glucan (1,4-alpha-), branching enzyme 1
	H3016D02	0.0172	Stt3a	Mm.2863	STT3, subunit of the oligosaccharyltransferase complex, homolog A
	H3052E08	0.0173	9130213B05Rik	Mm.5002	RIKEN cDNA 9130213B05 gene
	H634823	0.0173	Cetn2	Mm.24643	Centrin 2
	H3059E07	0.0173	Il17f	Mm.222807	Interleukin 17F
	H3155H02	0.0173	Prc1	Mm.227274	Protein regulator of cytokinesis 1
	H3066G05	0.0173	Wdr42a	Mm.159915	WD repeat domain 42A
	H3054D06	0.0176	Ovgp1	Mm.431947	Oviductal glycoprotein 1
	H3066E03	0.0177	BC025546	Mm.275341	CDNA sequence BC025546
	H3018F02	0.0177	Kifap3	Mm.4651	Kinesin-associated protein 3
	H3035F07	0.0177	LOC665306	Mm.358758	Hypothetical protein LOC665306
	H3104A09	0.0177	Lpgat1	Mm.277958	Lysophosphatidylglycerol acyltransferase 1
	H3030A08	0.0177	Rpa1	Mm.180734	Replication protein A1
	H553017	0.0177	Zbtb3	Mm.23423	Zinc finger and BTB domain containing 3
	H3104A03	0.0179	Espl1	Mm.288324	Extra spindle poles-like 1
	H3057E10	0.0180	D10Wsu102e	Mm.196210	DNA segment, Chr 10, Wayne State University 102, expressed

	NIA	ρ ≤	Common	Unigene	Name
	H3155F07	0.0182	2810008M24Rik	Mm.29464	RIKEN cDNA 2810008M24 gene
	H582040	0.0182	Hook3	Mm.334464	Hook homolog 3
	H3134C03	0.0183	Cnot2	Mm.351553	CCR4-NOT transcription complex, subunit 2
	H598316	0.0183	Rab1	Mm.271944	RAB1, member RAS oncogene family
	H3030G01	0.0184	Rpe	Mm.240912	Ribulose-5-phosphate-3-epimerase
	H3084C01	0.0184	Siah1a	Mm.324553	Seven in absentia 1A
	H557977	0.0185	Gimap3	Mm.333050	GTPase, IMAP family member 3
	H3120E08	0.0185	Pkp4	Mm.260938	Plakophilin 4
	H3090B04	0.0185	Usp1	Mm.371692	Ubiquitin specific peptdiase 1
	H3063A07	0.0189	Atp9b	Mm.247138	ATPas, class II, type 9B
	H538160	0.0189	Chd7	Mm.138792	Chromodomain helicase DNA binding protein 7
	H3048H01	0.0189	Hnrpc	Mm.427321	Heterogeneous nuclear ribonucleoprotein C
	H3070F03	0.0189	Nlrp4b	Mm.95244	NLR family, pyrin domain containing 4B
	H3078F08	0.0189	Spata2	Mm.34342	Spermatogenesis associated 2
	H3135D07	0.0191	Dhcr24	Mm.133370	24-dehydrocholesterol reductase
	H617913	0.0191	Nuak1	Mm.25874	NUAK family, SNF1-like kinase, 1
	H575740	0.0192	Ear2	Mm.327386	Eosinophil-associated, ribonuclease A family, member 2
	H3096C07	0.0192	Rbbp5	Mm.132868	Retinoblastoma binding protein 5
	H336029	0.0193	D10Bwg1379e	Mm.40491	DNA segment, Chr 10, Brigham & Women's Genetics 1379 expressed
	H657430	0.0193	Ranbp10	Mm.4206	RAN binding protein 10
	H637279	0.0194	Dscr1l2	Mm.331970	Down syndrome critical region gene 1-like 2
	H3094H01	0.0197	Otud1	Mm.83981	OTU domain containing 1
	H3018H09	0.0198	Csnk2a1	Mm.298893	Casein kinase 2, alpha 1 polypeptide
	H3103H02	0.0200	D11Ertd636e	Mm.229207	DNA segment, Chr 11, ERATO Doi 636, expressed
	H3013B01	0.0200	Taf3	Mm.86343	TAF3 RNA polymerase II, TATA box binding protein (TBP)-associated factor
	H3045D09	0.0201	Vps24	Mm.181278	Vacuolar protein sorting 24
	H3086F02	0.0204	Rtn4ip1	Mm.390253	Reticulon 4 interacting protein 1
	H3146G10	0.0204	Zzz3	Mm.425142	Zinc finger, ZZ domain containing 3
	H3027H08	0.0206	2900073G15Rik	Mm.261329	RIKEN cDNA 2900073G15 gene
	H3101A05	0.0206	Tmem16a	Mm.26700	Transmembrane protein 16A
	H637211	0.0207	Gda	Mm.45054	Guanine deaminase
	H3123E04	0.0207	Pols	Mm.315959	Polymerase (DNA directed) sigma
	H3126H06	0.0207	Zfp87	Mm.315879	Zinc finger protein 87
	H3025E01	0.0209	C330019G07Rik	Mm.335866	RIKEN cDNA C330019G07 gene
	H3042H08	0.0209	Spred2	Mm.266627	Sprouty-related, EVH1 domain containing 2
	H3090A04	0.0209	Strn	Mm.311915	Striatin, calmodulin binding protein

	NIA	ρ ≤	Common	Unigene	Name
	H3058D07	0.0211	Mmp23	Mm.29373	Matrix metallopeptidase 23
	H3030D01	0.0212	Abcb7	Mm.426128	ATP-binding cassette, sub-family B (MDR/TAP), member 7
	H3006F07	0.0212	Nmd3	Mm.21062	NMD3 homolog
	H3014E07	0.0212	Sec11c	Mm.27800	SEC11 homolog C
	H3039E11	0.0213	Foxn2	Mm.393597	Forkhead box N2
	H3023C12	0.0213	Pcqap	Mm.208970	Positive cofactor 2, multiprotein complex, glutamine/Q-rich-associated protein
	H636120	0.0215	Dolpp1	Mm.285167	Dolichyl pyrophosphate phosphatase 1
‡	H3089E09	0.0215	Ivns1abp	Mm.33764	Influenza virus NS1A binding protein
	H3020G01	0.0215	Pak1ip1	Mm.24789	PAK1 interacting protein 1
	H3043B08	0.0216	Trappc6b	Mm.290070	Trafficking protein particle complex 6B
	H3154F01	0.0217	data not found	Mm.427081	Transcribed locus, weakly similar to NP_001017510.1 protein LOC498750
	H3493036	0.0217	Nap1l1	Mm.432116	Nucleosome assembly protein 1-like 1
	H3030C07	0.0218	Large	Mm.324371	Like-glycosyltransferase
	H3051H06	0.0221	AU017455	Mm.435553	Expressed sequence AU017455
	H3016E01	0.0221	Prps1	Mm.268180	Phosphoribosyl pyrophosphate synthetase 1
	H3006E05	0.0224	Seh1l	Mm.307315	SEH1-like (S. cerevisiae
	H420319	0.0225	Psat1	Mm.289936	Phosphoserine aminotransferase 1
	H3066A03	0.0227	Usp42	Mm.259620	Ubiquitin specific peptidase 42
	H3124A04	0.0229	Msh6	Mm.18210	MutS homolog 6 (E. coli)
	H582683	0.0230	Herpud2	Mm.142843	HERPUD family member 2
	H330603	0.0232	2700081L22Rik	Mm.391869	RIKEN cDNA 2700081L22 gene
	H3017C02	0.0232	4930548G07Rik	Mm.152466	RIKEN cDNA 4930548G07 gene
	H3074G07	0.0232	C330018D20Rik	Mm.256874	RIKEN cDNA C330018D20 gene
	H3081F02	0.0232	Eif4enif1	Mm.255649	Eukaryotic translation initiation factor 4E nuclear import factor 1
	H3024C08	0.0232	Hax1	Mm.256035	HCLS1 associated X-1
	H3101D04	0.0232	Hrb	Mm.380683	HIV-1 Rev binding protein
	H3072C08	0.0232	Lrrfip1	Mm.45039	Leucine rich repeat (in FLII) interacting protein 1
	H613955	0.0232	Mcoln1	Mm.8356	Mucolipin 1
	H3101A02	0.0232	R3hdm2	Mm.29342	R3H domain containing 2
	H3070F02	0.0234	B3gnt2	Mm.258094	UDP-GlcNAc:betaGal beta-1,3-N-acetylglucosaminyltransferase 2
	H3115C07	0.0237	Opa1	Mm.274285	Optic atrophy 1 homolog
	H3074H01	0.0238	Syde2	Mm.268854	Synapse defective 1, Rho GTPase, homolog 2
	H3065D04	0.0242	Nlrp14	Mm.35644	NLR family, pyrin domain containing 14
	H3076A08	0.0243	Polr2h	Mm.288730	Polymerase (RNA) II (DNA directed) polypeptide H
	H3030B03	0.0243	Zfp655	Mm.206555	Zinc finger protein 655
	H3050E02	0.0245	Ccpg1	Mm.268475	Cell cycle progression 1

	NIA	ρ ≤	Common	Unigene	Name
	H3156C05	0.0245	Dusp7	Mm.275584	Dual specificity phosphatase 7
	H3078A07	0.0245	E030046B03Rik	Mm.11567	RIKEN cDNA E030046B03 gene
	H3149C01	0.0245	Mphosph10	Mm.26973	M-phase phosphoprotein 10 (U3 small nucleolar ribonucleoprotein)
	H3004F07	0.0245	Ptp4a2	Mm.193688	Protein tyrosine phosphatase 4a2
	H3101E10	0.0248	Lypla1	Mm.299955	Lysophospholipase 1
	H3080D03	0.0248	Tubb2b	Mm.379227	Tubulin, beta 2b
	H3067A07	0.0249	Kcmf1	Mm.29194	Potassium channel modulatory factor 1
	H3148D11	0.0249	Psma5	Mm.208883	Proteasome (prosome, macropain) subunit, alpha type 5
	H3056A02	0.0251	Klf6	Mm.275036	Kruppel-like factor 6
	H3112G08	0.0252	Lats2	Mm.347899	Large tumor suppressor 2
	H3106A07	0.0252	Nolc1	Mm.402190	Nucleolar and coiled-body phosphoprotein 1
	H3150E07	0.0254	5730593F17Rik	Mm.262113	RIKEN cDNA 5730593F17 gene
	H3127F07	0.0254	Cdc73	Mm.389191	Vcell division cycle 73, Paf1/RNA polymerase II complex component, homolog
	H3005H07	0.0256	Smap1l	Mm.271819	Stromal membrane-associated protein 1-like
	H3034D08	0.0256	Zc3h11a	Mm.245357	Zinc finger CCCH type containing 11A
	H3087G08	0.0256	Zfp654	Mm.25960	Zinc finger protein 654
	H3052B05	0.0257	Cul5	Mm.434362	Cullin 5
	H402552	0.0257	Kcnk1	Mm.10800	Potassium channel, subfamily K, member 1
	H3101C04	0.0257	Tbl1xr1	Mm.202966	Transducin (beta)-like 1X-linked receptor 1
	H3016H02	0.0258	Pold3	Mm.37562	Polymerase (DNA-directed), delta 3, accessory subunit
	H3030F07	0.0259	Copb2	Mm.400464	Coatomer protein complex, subunit beta 2 (beta prime)
	H3111F10	0.0259	Trim37	Mm.17436	Tripartite motif protein 37
	H644818	0.0261	Ccdc126	Mm.32416	Coiled-coil domain containing 126
	H3008B05	0.0261	Dag1	Mm.7524	Dystroglycan 1
	H597078	0.0261	Ikbkb	Mm.277886	Inhibitor of kappaB kinase beta
	H3032E09	0.0261	Synj2bp	Mm.279603	Synaptojanin 2 binding protein
	H3075E09	0.0261	Zfp535	Mm.340052	Zinc finger protein 535
	H3123D08	0.0262	Vps29	Mm.216528	Vacuolar protein sorting 29
	H3102E02	0.0263	Ppp2r5c	Mm.240396	Protein phosphatase 2, regulatory subunit B (B56), gamma isoform
	H3097F07	0.0264	AU040829	Mm.134338	Expressed sequence AU040829
	H3029F07	0.0265	Leo1	Mm.41508	Leo1, Paf1/RNA polymerase II complex component, homolog
	H3027F07	0.0266	Map2k6	Mm.14487	Mitogen activated protein kinase kinase 6
	H3059H12	0.0267	Plac1l	Mm.26881	Placenta-specific 1-like
	H3068A06	0.0268	Aytl2	Mm.284649	Acyltransferase like 2
	H3085E08	0.0268	LOC635617	Mm.435560	Hypothetical LOC635617
	H3073E02	0.0268	Pttg1	Mm.6856	Pituitary tumor-transforming 1

	NIA	ρ ≤	Common	Unigene	Name
	H3016D07	0.0272	Atp8b1	Mm.270043	ATPase, class I, type 8B, member 1
	H3026G07	0.0273	Bbx	Mm.28940	Bobby sox homolog
	H3015D01	0.0278	Tubb5	Mm.434402	Tubulin, beta 5
	H3708478	0.0279	Hnrpf	Mm.422979	Heterogeneous nuclear ribonucleoprotein F
	H3021H03	0.0279	Rsu1	Mm.905	Ras suppressor protein 1
	H3108C09	0.0279	Wfdc2	Mm.27289	WAP four-disulfide core domain 2
	H3158C11	0.0283	Gja4	Mm.24615	Gap junction membrane channel protein alpha 4
	H3131A10	0.0286	Sypl	Mm.246304	Synaptophysin-like protein
	H3013H07	0.0289	Hbld2	Mm.7884	HESB like domain containing 2
	H3010F07	0.0291	Pdcd6ip	Mm.29816	Programmed cell death 6 interacting protein
	H3158G03	0.0292	Kif9	Mm.42170	Kinesin family member 9
	H3103A05	0.0292	Pla2g4a	Mm.4186	Phospholipase A2, group IVA (cytosolic, calcium-dependent)
	H3080D09	0.0293	B230220E17Rik	Mm.241965	RIKEN cDNA B230220E17 gene
	H3036H07	0.0295	9430057O19Rik	Mm.21450	RIKEN cDNA 9430057O19 gene
	H3091F11	0.0295	Shoc2	Mm.228669	Soc-2 (suppressor of clear) homolog
	H3130G03	0.0296	BC017133	Mm.404420	CDNA sequence BC017133
	H3110C08	0.0297	Rnasen	Mm.293142	Ribonuclease III, nuclear
	H3151D08	0.0298	Dad1	Mm.319038	Defender against cell death 1
	H3063A08	0.0299	Lgmn	Mm.17185	Legumain
	H3075B01	0.0302	Baz2a	Mm.252213	Bromodomain adjacent to zinc finger domain, 2A
	H3012C02	0.0302	Canx	Mm.248827	Calnexin
	H3095A05	0.0302	Esr1	Mm.9213	Estrogen receptor 1 (alpha)
	H3070C01	0.0303	5730557L09Rik	Mm.392007	RIKEN cDNA 5730557L09 gene
	H3042B07	0.0303	Ccdc43	Mm.277638	Coiled-coil domain containing 43
	H3089F02	0.0303	Edem1	Mm.21596	ER degradation enhancer, mannosidase alpha-like 1
	H3102F02	0.0305	Ehd4	Mm.132226	EH-domain containing 4
	H3085A07	0.0307	data not found	Mm.410573	Transcribed locus
	H3091D08	0.0307	Gna14	Mm.313181	Guanine nucleotide binding protein, alpha 14
	H1477166	0.0307	Hnrpa2b1	Mm.155896	Heterogeneous nuclear ribonucleoprotein A2/B1
	H3008A08	0.0307	Melk	Mm.268668	Maternal embryonic leucine zipper kinase
	H3037C07	0.0307	Trim13	Mm.23959	Tripartite motif protein 13
	H3028H06	0.0308	Pdk1	Mm.34411	Pyruvate dehydrogenase kinase, isoenzyme 1
	H3132G09	0.0309	Atp6v1h	Mm.27082	ATPase, H+ transporting, lysosomal V1 subunit H
	H313744	0.0309	D14Ertd436e	Mm.287279	DNA segment, Chr 14, ERATO Doi 436, expressed
	H3038A11	0.0309	data not found	Mm.350239	Transcribed locus
	H3051C10	0.0309	Nrp1	Mm.271745	Neuropilin 1

	NIA	ρ ≤	Common	Unigene	Name
	H3022B07	0.0309	Pigs	Mm.295908	Phosphatidylinositol glycan anchor biosynthesis, class S
	H3034B01	0.0309	Ube2l3	Mm.3074	Ubiquitin-conjugating enzyme E2L 3
	H537177	0.0310	Chordc1	Mm.103534	Cysteine and histidine-rich domain (CHORD)-containing, zinc-binding protein 1
	H3149G02	0.0312	Smad3	Mm.7320	MAD homolog 3+E679
	H3101A12	0.0313	Ccrn4l	Mm.86541	CCR4 carbon catabolite repression 4-like
	H3076H09	0.0313	Unc13c	Mm.41035	Unc-13 homolog C
	H3013E02	0.0314	Bxdc2	Mm.28057	Brix domain containing 2
	H634707	0.0318	Ccnb1	Mm.260114	Cyclin B1
	H577160	0.0318	Crsp6	Mm.44151	Cofactor required for Sp1 transcriptional activation, subunit 6
	H3038B10	0.0318	Csnk2a2	Mm.51136	Casein kinase 2, alpha prime polypeptide
	H3089C02	0.0318	D2Ertd391e	Mm.28492	DNA segment, Chr 2, ERATO Doi 391, expressed
	H599008	0.0319	Cxcl12	Mm.303231	Chemokine (C-X-C motif) ligand 12
	H3057G07	0.0319	Dock7	Mm.260623	Dedicator of cytokinesis 7
	H3129E02	0.0319	Gtpbp8	Mm.34520	GTP-binding protein 8 (putative)
	H3122A02	0.0319	Hoxb3	Mm.342481	Homeo box B3
	H639132	0.0319	Slc14a1	Mm.33832	Solute carrier family 14 (urea transporter), member 1
	H3075E01	0.0321	Snx6	Mm.28240	Sorting nexin 6
	H582141	0.0324	Cfl2	Mm.276826	Cofilin 2, muscle
	H3060D01	0.0324	Cnot7	Mm.272551	CCR4-NOT transcription complex, subunit 7
	H3101E07	0.0324	Tjp2	Mm.104744	Tight junction protein 2
	H719317	0.0326	Lsm11	Mm.249827	U7 snRNP-specific Sm-like protein LSM11
	H3011F08	0.0329	Errfi1	Mm.318841	ERBB receptor feedback inhibitor 1
	H3087B01	0.0335	AU022751	Mm.199793	Expressed sequence AU022751
	H3113F04	0.0335	Nkiras1	Mm.25648	NFKB inhibitor interacting Ras-like protein 1
	H3113A01	0.0335	Slbp	Mm.4172	Stem-loop binding protein
	H524113	0.0335	Yipf6	Mm.212290	Yip1 domain family, member 6
	H3049D01	0.0338	Senp6	Mm.28232	SUMO/sentrin specific peptidase 6
	H3078G10	0.0341	Drd3	Mm.327835	Dopamine receptor 3
	H723423	0.0342	Cugbp2	Mm.431429	CUG triplet repeat, RNA binding protein 2
	H3101G07	0.0345	5730420B22Rik	Mm.28129	RIKEN cDNA 5730420B22 gene
	H3126A12	0.0345	Pole4	Mm.195753	Polymerase (DNA-directed), epsilon 4 (p12 subunit)
	H406754	0.0345	Thumpd1	Mm.26392	THUMP domain containing 1
	H3023F01	0.0345	Zwilch	Mm.335237	Zwilch, kinetochore associated, homolog
	H3151F04	0.0346	Odf2	Mm.330116	Outer dense fiber of sperm tails 2
	H585381	0.0347	D15Wsu169e	Mm.322931	DNA segment, Chr 15, Wayne State University 169, expressed
	H3082A03	0.0347	Glcci1	Mm.210787	Glucocorticoid induced transcript 1

	NIA	ρ ≤	Common	Unigene	Name
	H524155	0.0353	Ubqln1	Mm.182053	Ubiquilin 1
	H3026E11	0.0354	Rbm4b	Mm.426068	RNA binding motif protein 4B
	H3048E04	0.0357	Etnk1	Mm.272548	Ethanolamine kinase 1
	H3150C10	0.0359	0610010D24Rik	Mm.156217	RIKEN cDNA 0610010D24 gene
	H3157C07	0.0359	Sgol2	Mm.339711	Shugoshin-like 2
	H3078D09	0.0359	Trim28	Mm.15701	Tripartite motif protein 28
	H3003F10	0.0362	Id1	Mm.444	Inhibitor of DNA binding 1
	H3139A07	0.0363	Stt3b	Mm.296158	STT3, subunit of the oligosaccharyltransferase complex, homolog B
	H3055C08	0.0364	Rragc	Mm.220922	Ras-related GTP binding C
	H3023D07	0.0364	Ttll5	Mm.132172	Tubulin tyrosine ligase-like family, member 5
	H719336	0.0367	Zxda	Mm.426145	Zinc finger, X-linked, duplicated A
	H3060A08	0.0368	4933428G09Rik	Mm.29409	RIKEN cDNA 4933428G09 gene
	H3084F06	0.0368	Aup1	Mm.235303	Ancient ubiquitous protein
	H3103H03	0.0370	C1galt1	Mm.393184	Core 1 UDP-galactose:N-acetylgalactosamine-alpha-R beta 1,3-galactosyltransferase
	H614501	0.0370	Cpsf6	Mm.288682	Cleavage and polyadenylation specific factor 6
	H3077H08	0.0370	Rdh10	Mm.274376	Retinol dehydrogenase 10 (all-trans)
	H3115B01	0.0372	2610002J23Rik	Mm.391799	RIKEN cDNA 2610002J23 gene
	H3025E07	0.0372	Adprh	Mm.20047	ADP-ribosylarginine hydrolase
	H3094D02	0.0373	Gnb1	Mm.2344	Guanine nucleotide binding protein, beta 1
	H3081G08	0.0377	Arl4a	Mm.12723	ADP-ribosylation factor-like 4A
	H572811	0.0381	Kns2	Mm.325746	Kinesin 2
	H3067E07	0.0382	Arhgap20	Mm.26150	Rho GTPase activating protein 20
	H329599	0.0382	Prdm15	Mm.328741	PR domain containing 15
	H3023B05	0.0382	Sumo3	Mm.24433	SMT3 suppressor of mif two 3 homolog 3
	H3118E08	0.0391	Ibtk	Mm.22315	Inhibitor of Bruton agammaglobulinemia tyrosine kinase
	H3107B07	0.0391	Myst2	Mm.205400	MYST histone acetyltransferase 2
	H3087B02	0.0392	Pogz	Mm.274787	Pogo transposable element with ZNF domain
	H3130B02	0.0393	Eif4e	Mm.3941	Eukaryotic translation initiation factor 4E
	H3108H07	0.0394	Cttn	Mm.205601	Cortactin
	H330584	0.0394	Moxd1	Mm.285934	Monooxygenase, DBH-like 1
	H3132F10	0.0394	Tulp4	Mm.28251	Tubby like protein 4
	H636116	0.0396	Aass	Mm.18651	Aminoadipate-semialdehyde synthase
	H3109D06	0.0398	F8a	Mm.165807	Factor 8-associated gene A
	H3040D02	0.0399	Egln1	Mm.140619	EGL nine homolog 1
	H3062A04	0.0399	Rab32	Mm.31486	RAB32, member RAS oncogene family
	H3118C07	0.0401	Hspd1	Mm.378944	Heat shock protein 1 (chaperonin)

	NIA	ρ ≤	Common	Unigene	Name
	H3032H03	0.0401	Satb1	Mm.311655	Special AT-rich sequence binding protein 1
	H3089E12	0.0401	Sema3c	Mm.5071	Sema domain, immunoglobulin domain (Ig), short basic domain, secreted, (semaphorin) 3C
	H3036H01	0.0402	Edg2	Mm.4772	Endothelial differentiation, lysophosphatidic acid G-protein-coupled receptor, 2
	H580666	0.0402	Etfa	Mm.290853	Electron transferring flavoprotein, alpha polypeptide
	H524610	0.0407	Anln	Mm.282751	Anillin, actin binding protein+E769
	H3148C06	0.0407	Eea1	Mm.210035	Early endosome antigen 1
	H3122A05	0.0407	Il4	Mm.276360	Interleukin 4
	H3057C07	0.0408	data not found	Mm.410567	Transcribed locus
	H3001A07	0.0409	BC002163	Mm.375824	CDNA sequence BC002163
	H575105	0.0409	Nfkb1	Mm.256765	Nuclear factor of kappa light chain gene enhancer in B-cells 1, p105
	H3113H08	0.0410	Cul3	Mm.12665	Cullin 3
	H3067C12	0.0411	Jak2	Mm.275839	Janus kinase 2
	H570257	0.0412	Kctd10	Mm.238285	Potassium channel tetramerisation domain containing 10
	H3121F07	0.0412	Socs6	Mm.91920	Suppressor of cytokine signaling 6
	H3071G09	0.0413	C030046E11Rik	Mm.353838	RIKEN cDNA C030046E11 gene
	H3027G01	0.0413	Lsm2	Mm.165735	LSM2 homolog, U6 small nuclear RNA associated
	H3146C08	0.0413	Mcm6	Mm.4933	Minichromosome maintenance deficient 6 (MIS5 homolog
	H3103D12	0.0415	Dcamkl2	Mm.44490	Doublecortin and CaM kinase-like 2
	H3097G01	0.0415	Mlx	Mm.628	MAX-like protein X
	H3050D10	0.0415	Tardbp	Mm.22453	TAR DNA binding protein
	H3115D07	0.0416	Cops4	Mm.957	COP9 (constitutive photomorphogenic) homolog, subunit 4
	H3149A01	0.0417	Ccnd3	Mm.246520	Cyclin D3
	H3101C11	0.0419	Ddhd1	Mm.121918	DDHD domain containing 1
	H3061G10	0.0420	Ranbp17	Mm.159019	RAN binding protein 17
	H3060E07	0.0421	Exod1	Mm.274160	Exonuclease domain containing 1
	H3020H08	0.0421	Fndc3b	Mm.38832	Fibronectin type III domain containing 3B
	H3097C04	0.0422	Xbp1	Mm.434371	X-box binding protein 1
	H3025C03	0.0427	4833439L19Rik	Mm.24593	RIKEN cDNA 4833439L19 gene
	H3012C01	0.0428	Deb1	Mm.24848	Differentially expressed in B16F10 1
	H3074H02	0.0431	Acbd3	Mm.272981	Acyl-Coenzyme A binding domain containing 3
	H331981	0.0431	Tmcc3	Mm.23047	Transmembrane and coiled coil domains 3
	H3009C02	0.0432	Sdad1	Mm.86564	SDA1 domain containing 1
	H3017B07	0.0432	Supt16h	Mm.286066	Suppressor of Ty 16 homolog
	H635131	0.0436	Bmp2k	Mm.392853	BMP2 inducible kinase
	H3153B08	0.0436	Gabra3	Mm.102286	Gamma-aminobutyric acid (GABA-A) receptor, subunit alpha 3
	H3087F01	0.0437	Immt	Mm.235123	Inner membrane protein, mitochondrial

	NIA	ρ ≤	Common	Unigene	Name
	H577934	0.0437	Tsg101	Mm.241334	Tumor susceptibility gene 101
	H3052F01	0.0442	Dyrk2	Mm.45565	Dual-specificity tyrosine-(Y)-phosphorylation regulated kinase 2
	H3042C03	0.0444	Ap3m1	Mm.386781	Adaptor-related protein complex 3, mu 1 subunit
	H3003F07	0.0444	Calm1	Mm.285993	Calmodulin 1
	H3113G07	0.0445	Plcg2	Mm.192699	Phospholipase C, gamma 2
	H3121E10	0.0446	Eif1ay	Mm.294623	Eukaryotic translation initiation factor 1A, Y-linked
	H3017H04	0.0446	Gatad2a	Mm.270044	GATA zinc finger domain containing 2A
	H3066A10	0.0446	Ndfip2	Mm.290669	Nedd4 family interacting protein 2
	H516176	0.0453	Pole3	Mm.27444	Polymerase (DNA directed), epsilon 3 (p17 subunit)
	H3006A07	0.0455	Rcn2	Mm.1782	Reticulocalbin 2
	H3069G07	0.0455	Unc50	Mm.27404	Unc-50 homolog
	H3150E08	0.0462	Map4k5	Mm.341948	Mitogen-activated protein kinase kinase kinase kinase 5
	H3082C04	0.0463	9030611O19Rik	Mm.248938	RIKEN cDNA 9030611O19 gene
	H3146C11	0.0463	Plekhb2	Mm.292751	Pleckstrin homology domain containing, family B (evectins) member 2
	H596983	0.0463	Tspan5	Mm.31927	Tetraspanin 5
	H3002F11	0.0466	Lig3	Mm.277136	Ligase III, DNA, ATP-dependent
	H3013G07	0.0466	Tsn	Mm.426637	Translin
	H3087G09	0.0469	Selk	Mm.104491	Selenoprotein K
	H3130E07	0.0470	Snx8	Mm.27098	Sorting nexin 8
	H3144F03	0.0472	Arpc5l	Mm.38155	Actin related protein 2/3 complex, subunit 5-like
	H3092G09	0.0473	data not found	Mm.410501	Transcribed locus, moderately similar to NP_997348.1 protein LOC400754
	H3047E10	0.0473	Isg20l2	Mm.45744	Interferon stimulated exonuclease gene 20-like 2
	H3090B12	0.0473	Prune	Mm.14155	Prune homolog
	H3058A05	0.0474	Prkg1	Mm.381172	Protein kinase, cGMP-dependent, type I
	H3041D08	0.0474	Zfp161	Mm.29434	Zinc finger protein 161
	H3028E09	0.0475	Grb10	Mm.273117	Growth factor receptor bound protein 10
	H3055B08	0.0476	9030416H16Rik	Mm.278974	RIKEN cDNA 9030416H16 gene
	H643832	0.0478	Ccnt2	Mm.268672	Cyclin T2
	H3125E10	0.0478	Hmga2	Mm.157190	High mobility group AT-hook 2
	H3010H07	0.0478	Peli1	Mm.28957	Pellino 1
	H3092B08	0.0478	Rpia	Mm.17905	Ribose 5-phosphate isomerase A
	H537175	0.0478	Tbc1d19	Mm.28823	TBC1 domain family, member 19
	H3144D04	0.0479	Ccdc98	Mm.221269	Coiled-coil domain containing 98
	H3148D08	0.0480	Dhdds	Mm.100290	Dehydrodolichyl diphosphate synthase
	H3045A11	0.0480	Gspt1	Mm.325827	G1 to S phase transition 1
	H3031C07	0.0481	8430406I07Rik	Mm.386817	RIKEN cDNA 8430406I07 gene

	NIA	ρ ≤	Common	Unigene	Name
	H3056E01	0.0483	Arpc5	Mm.288974	Actin related protein 2/3 complex, subunit 5
	H580158	0.0484	Qdpr	Mm.30204	Quinoid dihydropteridine reductase
	H3057H02	0.0487	Prss35	Mm.257629	Protease, serine, 35
	H3085B03	0.0487	Rbm4	Mm.426069	RNA binding motif protein 4
	H3087H05	0.0490	Acat1	Mm.293233	Acetyl-Coenzyme A acetyltransferase 1
	H3089H02	0.0490	Efna5	Mm.435564	Ephrin A5
	H3044D09	0.0490	Scd1	Mm.193096	Stearoyl-Coenzyme A desaturase 1
	H651602	0.0490	Tm4sf1	Mm.856	Transmembrane 4 superfamily member 1
	H3142C09	0.0493	Sh3gl2	Mm.143603	SH3-domain GRB2-like 2
	H3092A02	0.0495	2500002B13Rik	Mm.426240	RIKEN cDNA 2500002B13 gene
	H3021C02	0.0495	Arpc4	Mm.289306	Actin related protein 2/3 complex, subunit 4
	H3114C07	0.0495	Bmi1	Mm.289584	B lymphoma Mo-MLV insertion region 1
	H3077H01	0.0497	AU019823	Mm.335149	Expressed sequence AU019823
	H3130C07	0.0497	Srfbp1	Mm.100617	Serum response factor binding protein 1
‡	Detected in SAGE analysis				
