# Supplementary material for: Ovarian gene expression in the absence of FIGLA, an oocyte-specific transcription factor
Source: BMC Dev Biol. 2007 Jun 13;7:67. doi: 10.1186/1471-213X-7-67 (PMC1906760; doi:10.1186/1471-213X-7-67)
Supplement: Additional file 6 — False discovery rate analysis of newborn microarray: genes potentially down-regulated by FIGLA [file 1471-213X-7-67-S6.pdf]

Additional file 6 – False discovery rate analysis of newborn microarray: genes potentially down-regulated by FIGLA

	NIA	ρ ≤	Common	Unigene	Name
	H427063	0.0000	Tmem178	Mm.357108	Transmembrane protein 178
	H3154D11	0.0000	Taf7l	Mm.103259	TAF7-like RNA polymerase II, TATA box binding protein (TBP)-associated factor
	H520363	0.0000	data not found	Mm.390888	Transcribed locus, weakly similar to XP_001075088.1 similar to glyceraldehyde-3-phosphate dehydrogenase
	H636923	0.0000	data not found	Mm.393093	Transcribed locus
	H3083F09	0.0000	Phtf1	Mm.385178	Putative homeodomain transcription factor 1
	H335579	0.0000	Slc20a1	Mm.272675	Solute carrier family 20, member 1
	H3091F07	0.0000	Gm1564	Mm.426692	Gene model 1564, (NCBI)
	H3039D12	0.0000	D6Ertd160e	Mm.24985	DNA segment, Chr 6, ERATO Doi 160, expressed
	H3046A06	0.0000	Auts2	Mm.217323	Autism susceptibility candidate 2
	H643709	0.0000	Tal1	Mm.239869	T-cell acute lymphocytic leukemia 1
	H651959	0.0000	B630005N14Rik	Mm.354863	RIKEN cDNA B630005N14 gene
	H581004	0.0000	Elovl2	Mm.2567	Elongation of very long chain fatty acids+E58
	H3154E03	0.0000	Daam2	Mm.211275	Dishevelled associated activator of morphogenesis 2
	H3089B04	0.0000	LOC433801	Mm.276881	Similar to RIKEN cDNA 6330416L07 gene
	H3076G04	0.0000	Cacnb2	Mm.313930	Calcium channel, voltage-dependent, beta 2 subunit
	H3154A06	0.0000	Gng13	Mm.171123	Guanine nucleotide binding protein 13, gamma
	H3033B06	0.0000	Birc1c	Mm.410435	Baculoviral IAP repeat-containing 1c
	H3098F09	0.0001	Vamp8	Mm.1838	Vesicle-associated membrane protein 8
	H3037D10	0.0001	Creg1	Mm.294885	Cellular repressor of E1A-stimulated genes 1
	H3156F03	0.0001	Prcp	Mm.389969	Prolylcarboxypeptidase (angiotensinase C)
	H334924	0.0001	Ilvbl	Mm.2644	IlvB (bacterial acetolactate synthase)-like
	H3121A01	0.0001	Ncam2	Mm.364726	Neural cell adhesion molecule 2
	H316656	0.0001	Bex2	Mm.94160	Brain expressed X-linked 2
	H3088F02	0.0001	D1Pas1	Mm.108054	DNA segment, Chr 1, Pasteur Institute 1
	H3083G04	0.0001	Jmjd1a	Mm.260479	Jumonji domain containing 1A
	H3147B03	0.0001	Myl9	Mm.271770	Myosin, light polypeptide 9, regulatory
	H3113B02	0.0001	Rhbdd3	Mm.260866	Rhomboid domain containing 3
	H3064E12	0.0001	Tsga10	Mm.332756	Testis specific 10
	H482270	0.0001	Pam	Mm.5121	Peptidylglycine alpha-amidating monooxygenase
	H3138E11	0.0001	Sema4g	Mm.34404	Sema domain, immunoglobulin domain (Ig), transmembrane domain (TM) and short cytoplasmic domain, (semaphorin) 4G
	H3128D05	0.0001	Six6os1	Mm.242892	Six6 opposite strand transcript 1
	H3155C04	0.0001	Ihpk1	Mm.276155	Inositol hexaphosphate kinase 1
	H493561	0.0001	Cma1	Mm.1252	Chymase 1, mast cell
	H3122H12	0.0001	Hba-a1	Mm.196110	Hemoglobin alpha, adult chain 1
	H643386	0.0001	2810008D09Rik	Mm.393072	RIKEN cDNA 2810008D09 gene
	H3042D02	0.0002	Bag3	Mm.84073	Bcl2-associated athanogene 3

	NIA	ρ ≤	Common	Unigene	Name
	H3149C02	0.0002	Stag3	Mm.285609	Stromal antigen 3
	H3102A08	0.0002	Cst12	Mm.280779	Cystatin 12
	H3104H05	0.0002	Rex2	Mm.388922	Reduced expression 2
	H476319	0.0002	Tsc22d3	Mm.22216	TSC22 domain family 3
	H3013D11	0.0002	Mt2	Mm.147226	Metallothionein 2
	H643249	0.0002	Wdr33	Mm.277705	WD repeat domain 33
	H3152H08	0.0002	Plp1	Mm.1268	Proteolipid protein (myelin) 1
	H3154E09	0.0002	Atp13a3	Mm.8924	ATPase type 13A3
	H3078E11	0.0002	Acsbg1	Mm.20592	Acyl-CoA synthetase bubblegum family member 1
	H3141G03	0.0002	Hmgcs2	Mm.289131	3-hydroxy-3-methylglutaryl-Coenzyme A synthase 2
	H3035D04	0.0003	Gm2a	Mm.287807	GM2 ganglioside activator protein
	H3150H11	0.0003	AU020206	Mm.200422	Expressed sequence AU020206
	H614363	0.0003	Rnpc3	Mm.316928	RNA-binding region (RNP1, RRM) containing 3
	H4019758	0.0003	Nek11	Mm.347509	NIMA (never in mitosis gene a)-related expressed kinase 11
	H3108A06	0.0003	Tnfrsf19	Mm.281356	Tumor necrosis factor receptor superfamily, member 19
	H3140A03	0.0003	Zfp503	Mm.292401	Zinc finger protein 503
	H3135E05	0.0004	A230106D06Rik	Mm.379388	RIKEN cDNA A230106D06 gene
	H641739	0.0004	Lef1	Mm.255219	Lymphoid enhancer binding factor 1
	H3059B12	0.0004	D330050I23Rik	Mm.230853	RIKEN cDNA D330050I23 gene
	H3118G02	0.0004	Ptpn13	Mm.3414	Protein tyrosine phosphatase, non-receptor type 13
	H3034D10	0.0004	Zfp248	Mm.212572	Zinc finger protein 248
	H3025F11	0.0004	EG433273	Mm.304088	Predicted gene, EG433273
	H313136	0.0004	4933417E01Rik	Mm.333647	RIKEN cDNA 4933417E01 gene
	H3152D03	0.0004	C330002I19Rik	Mm.250641	RIKEN cDNA C330002I19 gene
	H3108A04	0.0004	Clu	Mm.200608	Clusterin
	H3036C11	0.0004	Luc7l2	Mm.276133	LUC7-like 2+E108
	H483686	0.0004	Zfp740	Mm.352945	Zinc finger protein 740
	H3031D03	0.0005	Aldoa	Mm.275831	Aldolase 1, A isoform
	H482191	0.0005	P4ha1	Mm.2212	Procollagen-proline, 2-oxoglutarate 4-dioxygenase (proline 4-hydroxylase), alpha 1 polypeptide
	H3012G09	0.0005	Fancc	Mm.126106	Fanconi anemia, complementation group C
	H3098D09	0.0006	Adamts16	Mm.115970	A disintegrin-like and metallopetidase (reprolysin type) with thrombospondin type 1 motif, 16
	H3126F11	0.0006	Mfge8	Mm.1451	Milk fat globule-EGF factor 8 protein
	H3003C11	0.0006	1110019N10Rik	Mm.279	RIKEN cDNA 1110019N10 gene
	H3114C08	0.0006	Eraf	Mm.423023	Erythroid associated factor
	H3152D12	0.0006	Col3a1	Mm.249555	Procollagen, type III, alpha 1
	H614586	0.0006	Grem2	Mm.25760	Gremlin 2 homolog, cysteine knot superfamily
	H3109D03	0.0006	Lamp2	Mm.486	Lysosomal membrane glycoprotein 2
	H616738	0.0006	Tlr3	Mm.33874	Toll-like receptor 3
	H3108G05	0.0006	Gtf2h1	Mm.22700	General transcription factor II H, polypeptide 1
	H3147G12	0.0006	Aff3	Mm.336679	AF4/FMR2 family, member 3

	NIA	ρ ≤	Common	Unigene	Name
	H3101C10	0.0006	EG382450	Mm.422788	Predicted gene, EG382450
	H658174	0.0006	Smg6	Mm.288460	Smg-6 homolog, nonsense mediated mRNA decay factor
	H3112G09	0.0007	Atp6v0d1	Mm.17708	ATPase, H+ transporting, lysosomal V0 subunit D1
	H3090D05	0.0007	data not found	Mm.410578	Transcribed locus
	H618819	0.0007	data not found	Mm.426407	16 days embryo head cDNA, RIKEN full-length enriched library, clone:C130083E18
	H3045B04	0.0007	Nlk	Mm.9001	Nemo like kinase
	H371729	0.0007	Tnrc15	Mm.23065	Trinucleotide repeat containing 15
	H3079C06	0.0007	Zdhhc23	Mm.327852	Zinc finger, DHHC domain containing 23
	H3115F09	0.0007	Sgce	Mm.8739	Sarcoglycan, epsilon
	H334928	0.0007	Lrp5	Mm.274581	Low density lipoprotein receptor-related protein 5
	H3083C10	0.0007	data not found	Mm.426532	Transcribed locus, moderately similar to XP_575695
	H313341	0.0008	Bace2	Mm.97885	Beta-site APP-cleaving enzyme 2
	H3019G02	0.0008	Mbnl3	Mm.295324	Muscleblind-like 3+E135
	H3099E06	0.0008	3110007F17Rik	Mm.292979	RIKEN cDNA 3110007F17 gene
	H3145H01	0.0008	Nxf2	Mm.103155	Nuclear RNA export factor 2
	H3011C03	0.0008	Pcyox1	Mm.30849	Prenylcysteine oxidase 1
	H3128F03	0.0009	Traf6	Mm.292729	Tnf receptor-associated factor 6
	H3151D11	0.0010	Psap	Mm.277498	Prosaposin
	H3121G05	0.0010	Cyp51	Mm.46044	Cytochrome P450, family 51
	H3119C11	0.0010	Cdc42bpb	Mm.27397	Cdc42 binding protein kinase beta
	H574685	0.0010	Glt8d3	Mm.22819	Glycosyltransferase 8 domain containing 3
	H3046B11	0.0010	Gata6	Mm.329287	GATA binding protein 6
	H3135H09	0.0011	Dip3b	Mm.282985	Dip3 beta
	H3128B04	0.0011	Lsr	Mm.4067	Lipolysis stimulated lipoprotein receptor
	H3043B09	0.0011	Cul4b	Mm.327675	Cullin 4B
	H3005D11	0.0011	Trim8	Mm.392177	Tripartite motif protein 8
	H3149A03	0.0011	Speg	Mm.275397	SPEG complex locus
	H596455	0.0012	Adcy7	Mm.288206	Adenylate cyclase 7
	H3125A02	0.0012	EG666017	Mm.425641	Predicted gene, EG666017
	H3047H09	0.0012	data not found	Mm.410559	Transcribed locus, moderately similar to NP_001041426.1 protein LOC500712
	H3142E09	0.0012	Tssc4	Mm.139675	Tumor-suppressing subchromosomal transferable fragment 4
	H3087F05	0.0013	Tsr2	Mm.8142	TSR2, 20S rRNA accumulation, homolog
	H3138C09	0.0013	Rnf128	Mm.27764	Ring finger protein 128
	H352778	0.0013	St8sia4	Mm.306228	ST8 alpha-N-acetyl-neuraminide alpha-2,8-sialyltransferase 4
	H3057F05	0.0013	Rabepk	Mm.158705	Rab9 effector protein with kelch motifs
	H3142F09	0.0013	Stx17	Mm.171334	Syntaxin 17
	H3099D04	0.0013	Cox5b	Mm.288128	Cytochrome c oxidase, subunit Vb
	H3127F11	0.0014	Ugcgl2	Mm.213406	UDP-glucose ceramide glucosyltransferase-like 2
	H3152B05	0.0014	Hipk1	Mm.20827	Homeodomain interacting protein kinase 1
	H722834	0.0014	Dock2	Mm.380679	Dedicator of cyto-kinesis 2

	NIA	ρ ≤	Common	Unigene	Name
	H3137E11	0.0015	Il11ra1	Mm.193451	Interleukin 11 receptor, alpha chain 1
	H598561	0.0015	Eif2ak4	Mm.217616	Eukaryotic translation initiation factor 2 alpha kinase 4
	H3115H09	0.0015	Emx2	Mm.245394	Empty spiracles homolog 2+E165
	H3061B11	0.0016	Pcdh9	Mm.37126	Protocadherin 9
	H636322	0.0016	Serpinb9	Mm.272569	Serine (or cysteine) peptidase inhibitor, clade B, member 9
	H3109A04	0.0016	Malat1	Mm.298256	Metastasis associated lung adenocarcinoma transcript 1 (non-coding RNA)
	H3118H04	0.0016	data not found	Mm.388432	Transcribed locus
	H3148F08	0.0018	Speer4a	Mm.389607	Spermatogenesis associated glutamate (E)-rich protein 4a
	H581667	0.0018	2700007P21Rik	Mm.381231	RIKEN cDNA 2700007P21 gene
	H575359	0.0018	data not found	Mm.426368	12 days embryo embryonic body+E226, RIKEN full-length enriched library, clone:9430093C18
	H580807	0.0019	D0HXS9928E	Mm.4370	DNA segment, human DXS9928E
	H3032F06	0.0019	Hbb-b1	Mm.288567	Hemoglobin, beta adult major chain
	H478560	0.0019	Hspb8	Mm.21549	Heat shock protein 8
	H3019C05	0.0019	AU022875	Mm.426334	Expressed sequence AU022875
	H3117D06	0.0019	Sumf1	Mm.379094	Sulfatase modifying factor 1
	H3131F05	0.0020	Tex101	Mm.23385	Testis expressed gene 101
	H3095F05	0.0020	Tmem176a	Mm.27061	Transmembrane protein 176A
	H3124B09	0.0021	App	Mm.277585	Amyloid beta (A4) precursor protein
	H596846	0.0021	Morc3	Mm.287329	Microrchidia 3
	H3130C01	0.0021	1200016E24Rik	Mm.332931	RIKEN cDNA 1200016E24 gene
	H3140A09	0.0021	Cpt1a	Mm.18522	Carnitine palmitoyltransferase 1a, liver
	H3139H12	0.0021	Hnrpa2b1	Mm.155896	Heterogeneous nuclear ribonucleoprotein A2/B1
	H3023H12	0.0021	Ldha	Mm.29324	Lactate dehydrogenase A
	H316867	0.0021	Dnm3os	Mm.383186	Dynamin 3, opposite strand
	H3117G06	0.0023	Pygb	Mm.222584	Brain glycogen phosphorylase
	H3127G12	0.0023	Amy2	Mm.331313	Amylase 2, pancreatic
	H2648673	0.0024	Phyhd1	Mm.297949	Phytanoyl-CoA dioxygenase domain containing 1
	H519148	0.0024	Tfrc	Mm.28683	Transferrin receptor
	H3109G05	0.0024	Pdgfd	Mm.390122	Platelet-derived growth factor, D polypeptide
	H573287	0.0024	EG666451	Mm.390434	Predicted gene, EG666451
	H720681	0.0024	Nfat5	Mm.390057	Nuclear factor of activated T-cells 5
	H636169	0.0024	Klhl5	Mm.10281	Kelch-like 5+E194
	H622781	0.0025	Samd9l	Mm.196013	Sterile alpha motif domain containing 9-like
	H3056B11	0.0025	Dnaja3	Mm.325524	DnaJ (Hsp40) homolog, subfamily A, member 3
	H620197	0.0026	3110043O21Rik	Mm.331544	RIKEN cDNA 3110043O21 gene
	H3109H12	0.0026	Tmem176b	Mm.28385	Transmembrane protein 176B
	H3070E05	0.0027	Nol4	Mm.209896	Nucleolar protein 4
	H3134D04	0.0028	Hk1	Mm.196605	Hexokinase 1
	H3145D10	0.0028	Kcnd2	Mm.320691	Potassium voltage-gated channel, Shal-related family, member 2
	H3157B02	0.0028	Cd200	Mm.245851	Cd200 antigen

	NIA	ρ ≤	Common	Unigene	Name
	H3669923	0.0028	data not found	Mm.30806	Transcribed locus, moderately similar to XP_362236.1 protein MG04681.4 [Magnaporthe grisea 70-15]
	H596904	0.0029	Smpdl3a	Mm.2379	Sphingomyelin phosphodiesterase, acid-like 3A
	H3069D09	0.0030	Ankra2	Mm.209642	Ankyrin repeat, family A (RFXANK-like), 2
	H3122E12	0.0030	Atp5b	Mm.238973	ATP synthase, H+ transporting mitochondrial F1 complex, beta subunit
	H3138F12	0.0031	Sdc4	Mm.3815	Syndecan 4
	H3097B10	0.0032	Mll5	Mm.205190	Myeloid/lymphoid or mixed-lineage leukemia 5
	H3024D11	0.0032	Kif7	Mm.101633	Kinesin family member 7
	H3068H08	0.0032	Zfp53	Mm.42140	Zinc finger protein 53
	H3064B09	0.0032	Heatr1	Mm.342328	HEAT repeat containing 1
	H3010B04	0.0033	Wdr75	Mm.270376	WD repeat domain 75
	H3066C06	0.0033	Sfpq	Mm.257276	Splicing factor proline/glutamine rich (polypyrimidine tract binding protein associated)
	H3080D05	0.0034	Stk4	Mm.234472	Serine/threonine kinase 4
	H3148D04	0.0034	Maged1	Mm.27578	Melanoma antigen, family D, 1
	H568119	0.0034	Prnd	Mm.180750	Prion protein dublet
	H3115G09	0.0034	Hba-x	Mm.141758	Hemoglobin X, alpha-like embryonic chain in Hba complex
	H514319	0.0034	Ndufa5	Mm.275780	NADH dehydrogenase (ubiquinone) 1 alpha subcomplex, 5
	H3145H05	0.0035	Ankrd47	Mm.196330	Ankyrin repeat domain 47
	H662847	0.0035	Hnrpul2	Mm.347805	Heterogeneous nuclear ribonucleoprotein U-like 2
	H3113A12	0.0035	Gas5	Mm.270065	Growth arrest specific 5
	H3090A03	0.0035	Dmt2	Mm.11171	Dorso-medial telencephalon gene 2
	H3130B05	0.0035	Gemin4	Mm.188413	Gem (nuclear organelle) associated protein 4
	H3118D03	0.0035	Arbp	Mm.5286	Acidic ribosomal phosphoprotein P0
	H599017	0.0035	Prdx3	Mm.29821	Peroxiredoxin 3
	H636312	0.0036	data not found	Mm.269151	Transcribed locus
	H596896	0.0036	Pex12	Mm.102205	Peroxisomal biogenesis factor 12
	H3123D05	0.0036	Pygl	Mm.256926	Liver glycogen phosphorylase
	H355433	0.0037	Trim46	Mm.331156	Tripartite motif protein 46
	H3156D09	0.0037	Epb4.1	Mm.30038	Erythrocyte protein band 4.1
	H3111A09	0.0037	4921511K06Rik	Mm.251303	RIKEN cDNA 4921511K06 gene
	H3031D04	0.0038	Ptp4a1	Mm.374437	Protein tyrosine phosphatase 4a1
	H403610	0.0038	Ykt6	Mm.294821	YKT6 homolog+E233
	H3156E12	0.0038	Smc1b	Mm.182737	Structural maintenace of chromosomes 1B
	H386829	0.0038	Stk38	Mm.435098	Serine/threonine kinase 38
	H317273	0.0039	2310036O22Rik	Mm.196005	RIKEN cDNA 2310036O22 gene
	H3101H03	0.0039	Chmp2b	Mm.432944	Chromatin modifying protein 2B
	H620242	0.0039	B230325K18Rik	Mm.32145	RIKEN cDNA B230325K18 gene
	H3084H03	0.0040	1600020E01Rik	Mm.349174	RIKEN cDNA 1600020E01 gene
	H3157D01	0.0040	Got2	Mm.230169	Glutamate oxaloacetate transaminase 2, mitochondrial
	H641939	0.0040	Lrrfip1	Mm.45039	Leucine rich repeat (in FLII) interacting protein 1
	H598351	0.0040	Mocs2	Mm.19027	Molybdenum cofactor synthesis 2

	NIA	ρ ≤	Common	Unigene	Name
	H3121C06	0.0041	Rag1ap1	Mm.17958	Recombination activating gene 1 activating protein 1
	H3136G06	0.0041	Rpl13a	Mm.180458	Ribosomal protein L13a
	H3112B07	0.0042	Acaa2	Mm.245724	Acetyl-Coenzyme A acyltransferase 2 (mitochondrial 3-oxoacyl-Coenzyme A thiolase)
	H3017B11	0.0042	Pgrmc1	Mm.9052	Progesterone receptor membrane component 1
	H3144A04	0.0042	Sacs	Mm.103675	Sacsin
	H3132E04	0.0043	Ndfip1	Mm.102496	Nedd4 family interacting protein 1
	H3079D12	0.0043	Pld3	Mm.6483	Phospholipase D family, member 3
	H3025H11	0.0044	Pcmtd2	Mm.271986	Protein-L-isoaspartate (D-aspartate) O-methyltransferase domain containing 2
	H619921	0.0044	*C030011O14Rik*	Mm.135018	RIKEN cDNA C030011O14 gene
	H3035E12	0.0044	data not found	Mm.410553	Transcribed locus
	H578171	0.0044	Il7r	Mm.389	Interleukin 7 receptor
	H635976	0.0045	Synj2bp	Mm.279603	Synaptojanin 2 binding protein
	H3005H12	0.0045	Son	Mm.46401	Son cell proliferation protein
	H3117A05	0.0046	Dync1h1	Mm.181430	Dynein cytoplasmic 1 heavy chain 1
	H3034C03	0.0046	data not found	Mm.410552	Transcribed locus, moderately similar to XP_575144.1 similar to LRRGT00150
	H482535	0.0046	Atp1a1	Mm.280103	ATPase, Na+/K+ transporting, alpha 1 polypeptide
	H3047F12	0.0046	Atf6	Mm.377046	Activating transcription factor 6
	H722408	0.0046	Mllt6	Mm.23685	Myeloid/lymphoid or mixed lineage-leukemia translocation to 6 homolog
	H614970	0.0049	3321401G04Rik	Mm.24652	RIKEN cDNA 3321401G04 gene
	H651726	0.0050	Tanc2	Mm.22501	Tetratricopeptide repeat, ankyrin repeat and coiled-coil containing 2
	H3126B08	0.0051	Pla2g7	Mm.9277	Phospholipase A2, group VII (platelet-activating factor acetylhydrolase, plasma)
	H334851	0.0051	Fbxo17	Mm.358659	F-box protein 17
	H3125H05	0.0051	Rps3a	Mm.399829	Ribosomal protein S3a
	H3109F10	0.0051	Mal2	Mm.434394	Mal, T-cell differentiation protein 2
	H603970	0.0052	Lmtk3	Mm.44928	Lemur tyrosine kinase 3
	H3029D06	0.0052	Tubb5	Mm.432391	Tubulin, beta 5
	H515230	0.0053	4930550L11Rik	Mm.391968	RIKEN cDNA 4930550L11 gene
	H3077D05	0.0054	Npc2	Mm.282556	Niemann Pick type C2
	H3072B09	0.0054	3110068G20Rik	Mm.392589	RIKEN cDNA 3110068G20 gene
	H3711711	0.0054	Stat5b	Mm.34064	Signal transducer and activator of transcription 5B
	H3079G08	0.0055	Ufc1	Mm.2815	Ubiquitin-fold modifier conjugating enzyme 1
	H3058C05	0.0055	Cyp11a1	Mm.302865	Cytochrome P450, family 11, subfamily a, polypeptide 1
	H3146F11	0.0055	Nrbp2	Mm.101946	Nuclear receptor binding protein 2
	H3140F04	0.0055	Anxa6	Mm.265347	Annexin A6
	H3042C02	0.0056	Pqlc1	Mm.29247	PQ loop repeat containing 1
	H3135H12	0.0056	Ccdc64	Mm.233914	Coiled-coil domain containing 64
	H596025	0.0056	D830007B15Rik	Mm.434319	RIKEN cDNA D830007B15 gene
	H3115A11	0.0056	Nod1	Mm.28498	Nucleotide-binding oligomerization domain containing 1
	H599930	0.0056	data not found	Mm.383435	Transcribed locus
	H3123B06	0.0056	Edd1	Mm.275426	E3 ubiquitin protein ligase, HECT domain containing, 1

	NIA	ρ ≤	Common	Unigene	Name
	H3143B05	0.0057	Rbm39	Mm.392436	RNA binding motif protein 39
	H3023A11	0.0058	Esf1	Mm.21228	ESF1, nucleolar pre-rRNA processing protein, homolog
	H3144D09	0.0059	Slc35d2	Mm.133731	Solute carrier family 35, member D2
	H3128E04	0.0059	Opn1sw	Mm.56987	Opsin 1 (cone pigments), short-wave-sensitive (color blindness, tritan)
	H3151F05	0.0060	Pkp2	Mm.2252	Plakophilin 2
	H3147B06	0.0061	Gnaq	Mm.391165	Guanine nucleotide binding protein, alpha q polypeptide
	H596885	0.0061	data not found	Mm.364924	Transcribed locus
	H3134E06	0.0061	3930401B19Rik	Mm.391849	RIKEN cDNA 3930401B19 gene
	H3111H06	0.0061	Kcnb2	Mm.382301	Potassium voltage gated channel, Shab-related subfamily, member 2
	H3086G06	0.0062	C330024D21Rik	Mm.387187	RIKEN cDNA C330024D21 gene
	H3024B02	0.0062	Sec61a1	Mm.28375	Sec61 alpha 1 subunit+E316
	H635962	0.0062	Tfdp2	Mm.390208	Transcription factor Dp 2
	H3136H05	0.0063	Atp5g1	Mm.371547	ATP synthase, H+ transporting, mitochondrial F0 complex, subunit c (subunit 9), isoform 1
	H614369	0.0063	Rab31	Mm.29274	RAB31, member RAS oncogene family
	H575476	0.0063	Hmgcl	Mm.297251	3-hydroxy-3-methylglutaryl-Coenzyme A lyase
	H3041B12	0.0064	Tug1	Mm.390314	Taurine upregulated gene 1
	H3110F06	0.0065	Pkia	Mm.3193	Protein kinase inhibitor, alpha
	H573951	0.0065	Klhl26	Mm.187090	Kelch-like 26+E496
	H576753	0.0065	Rbm4b	Mm.426068	RNA binding motif protein 4B
	H637845	0.0066	data not found	Mm.4913	Transcribed locus
	H3152B08	0.0066	Dnajc15	Mm.248046	DnaJ (Hsp40) homolog, subfamily C, member 15
	H3112D03	0.0066	Pex19	Mm.247764	Peroxisome biogenesis factor 19
	H347933	0.0066	Ddah2	Mm.1457	Dimethylarginine dimethylaminohydrolase 2
	H570043	0.0066	Gipc1	Mm.20945	GIPC PDZ domain containing family, member 1
	H621840	0.0067	Srgap3	Mm.236401	SLIT-ROBO Rho GTPase activating protein 3
	H3063G06	0.0067	data not found	Mm.173536	Transcribed locus
	H3116H04	0.0067	Pggt1b	Mm.393044	Protein geranylgeranyltransferase type I, beta subunit
	H642365	0.0067	Centg2	Mm.291135	Centaurin, gamma 2
	H3127B06	0.0069	Kitl	Mm.45124	Kit ligand
	H355186	0.0070	Tmem86a	Mm.27338	Transmembrane protein 86A
	H3130F05	0.0071	Crsp2	Mm.17616	Cofactor required for Sp1 transcriptional activation, subunit 2
	H481530	0.0071	Arf3	Mm.221298	ADP-ribosylation factor 3
	H3010C07	0.0071	Irx3	Mm.238044	Iroquois related homeobox 3
	H3005F06	0.0072	Irak1	Mm.38241	Interleukin-1 receptor-associated kinase 1
	H3129C03	0.0072	Slc4a1	Mm.7248	Solute carrier family 4 (anion exchanger), member 1
	H3027E09	0.0072	Eno1	Mm.70666	Enolase 1, alpha non-neuron
	H419490	0.0072	Iqce	Mm.179704	IQ motif containing E
	H3009B05	0.0072	Rpl27a	Mm.305750	Ribosomal protein L27a
	H3073D08	0.0073	data not found	Mm.423302	RNA binding site for Dazl protein, clone kc7
	H3058E09	0.0074	Dgke	Mm.153695	Diacylglycerol kinase, epsilon

	NIA	ρ ≤	Common	Unigene	Name
	H3099F04	0.0074	Fnbp4	Mm.314887	Formin binding protein 4
	H404427	0.0074	Pdgfa	Mm.2675	Platelet derived growth factor, alpha
	H3113H03	0.0074	Ddx26b	Mm.72753	DEAD/H (Asp-Glu-Ala-Asp/His) box polypeptide 26B
	H637208	0.0078	data not found	Mm.34484	Transcribed locus
	H3112D10	0.0078	Wdr89	Mm.341719	WD repeat domain 89
	H3044D11	0.0078	C4bp	Mm.306720	Complement component 4 binding protein
	H3120H05	0.0080	Sdha	Mm.158231	Succinate dehydrogenase complex, subunit A, flavoprotein (Fp)
	H3137H06	0.0081	Aes	Mm.180013	Amino-terminal enhancer of split
	H637814	0.0081	data not found	Mm.30630	Transcribed locus
	H331449	0.0081	Lemd2	Mm.29689	LEM domain containing 2
	H597897	0.0081	C330002G24Rik	Mm.361682	RIKEN cDNA C330002G24 gene
	H3107B09	0.0082	Tfb2m	Mm.293529	Transcription factor B2, mitochondrial
	H619261	0.0082	4930546H06Rik	Mm.227456	RIKEN cDNA 4930546H06 gene
	H3134F11	0.0083	Men1	Mm.12917	Multiple endocrine neoplasia 1
	H652719	0.0083	Zfp96	Mm.24124	Zinc finger protein 96
	H574155	0.0083	H2-Eb1	Mm.22564	Histocompatibility 2, class II antigen E beta
	H3158F11	0.0084	Nnp1	Mm.38344	Novel nuclear protein 1
	H3117D07	0.0084	Cd63	Mm.277857	Cd63 antigen
	H3137G08	0.0084	Gab2	Mm.42033	Growth factor receptor bound protein 2-associated protein 2
	H3030D10	0.0085	Pkm2	Mm.326167	Pyruvate kinase, muscle
	H3139F09	0.0085	Mccc1	Mm.425129	Methylcrotonoyl-Coenzyme A carboxylase 1 (alpha)
	H3056H09	0.0086	Fndc5	Mm.44075	Fibronectin type III domain containing 5
	H3125G06	0.0089	Rpl31	Mm.379302	Ribosomal protein L31
	H367410	0.0089	Chgb	Mm.255241	Chromogranin B
	H618848	0.0093	Ltbp1	Mm.269747	Latent transforming growth factor beta binding protein 1
	H3116F03	0.0093	data not found	Mm.275414	Transcribed locus
	H3103B10	0.0094	1700095A21Rik	Mm.393245	RIKEN cDNA 1700095A21 gene
	H427644	0.0094	Aplp1	Mm.2381	Amyloid beta (A4) precursor-like protein 1
	H3132D09	0.0095	Usp3	Mm.38976	Ubiquitin specific peptidase 3
	H3116G06	0.0096	Adamts6	Mm.208125	A disintegrin-like and metallopeptidase (reprolysin type) with thrombospondin type 1 motif, 6
	H3090A05	0.0096	data not found	Mm.26003	Transcribed locus
	H615776	0.0098	Ppp2r5e	Mm.259626	Protein phosphatase 2, regulatory subunit B (B56), epsilon isoform
	H618006	0.0100	data not found	Mm.384709	Transcribed locus
	H3098G02	0.0100	data not found	Mm.410503	Transcribed locus
	H3098H04	0.0101	Zbtb20	Mm.136238	Zinc finger and BTB domain containing 20
	H3069E09	0.0102	Atp1b2	Mm.235204	ATPase, Na+/K+ transporting, beta 2 polypeptide
	H3150C09	0.0102	Limk2	Mm.390323	LIM motif-containing protein kinase 2
	H3060A11	0.0102	Pbx3	Mm.239941	Pre B-cell leukemia transcription factor 3
	H3134C11	0.0103	Gars	Mm.250004	Glycyl-tRNA synthetase
	H3081B05	0.0104	Mier1	Mm.288721	Mesoderm induction early response 1 homolog

	NIA	ρ ≤	Common	Unigene	Name
	H3142G06	0.0105	2610005L07Rik	Mm.359054	RIKEN cDNA 2610005L07 gene
	H3068D02	0.0106	data not found	Mm.351390	Transcribed locus
	H3036E04	0.0107	Magi2	Mm.332231	Membrane associated guanylate kinase, WW and PDZ domain containing 2
	H331081	0.0110	Slc6a2	Mm.57040	Solute carrier family 6 (neurotransmitter transporter, noradrenalin), member 2
	H402902	0.0112	Arfip2	Mm.41637	ADP-ribosylation factor interacting protein 2
	H3034C05	0.0112	D230025D16Rik	Mm.334761	RIKEN cDNA D230025D16 gene
	H3068H12	0.0113	Dock4	Mm.341423	Dedicator of cytokinesis 4
	H3107H11	0.0114	4930451C15Rik	Mm.38634	RIKEN cDNA 4930451C15 gene
	H3023D12	0.0114	E4f1	Mm.163132	E4F transcription factor 1
	H3048D12	0.0114	Gnpnat1	Mm.312945	Glucosamine-phosphate N-acetyltransferase 1
	H539393	0.0115	2610002F03Rik	Mm.27103	RIKEN cDNA 2610002F03 gene
	H3045F08	0.0115	Tktl1	Mm.25057	Transketolase-like 1
	H3114C04	0.0116	Trim44	Mm.315002	Tripartite motif-containing 44
	H3127F04	0.0117	Chst11	Mm.360747	Carbohydrate sulfotransferase 11
	H3032F09	0.0117	Lnpep	Mm.328807	Leucyl/cystinyl aminopeptidase
	H3158F12	0.0118	1700020I14Rik	Mm.379181	RIKEN cDNA 1700020I14 gene
	H3111H03	0.0118	Nfia	Mm.31274	Nuclear factor I/A
	H3063E06	0.0120	4930422I07Rik	Mm.259988	RIKEN cDNA 4930422I07 gene
	H3116B06	0.0120	Snn	Mm.325800	Stannin
	H3149B12	0.0123	Pabpn1	Mm.7723	Poly(A) binding protein, nuclear 1
	H3023G10	0.0126	Nkiras2	Mm.274734	NFKB inhibitor interacting Ras-like protein 2
	H3088E04	0.0127	Csnk1a1	Mm.26908	Casein kinase 1, alpha 1
	H3153H05	0.0127	Peci	Mm.28883	Peroxisomal delta3, delta2-enoyl-Coenzyme A isomerase
	H3133D11	0.0129	Zfp623	Mm.273264	Zinc finger protein 623
	H3013F01	0.0130	Alad	Mm.6988	Aminolevulinate, delta-, dehydratase
	H3120H03	0.0130	Alcam	Mm.288282	Activated leukocyte cell adhesion molecule
	H354692	0.0130	Alkbh3	Mm.272498	AlkB, alkylation repair homolog 3 (E. coli)
	H3079H05	0.0131	Baz2b	Mm.228071	Bromodomain adjacent to zinc finger domain, 2B
	H3152F01	0.0131	Cryl1	Mm.25539	Crystallin, lambda 1
	H3067D12	0.0132	2310008H04Rik	Mm.244703	RIKEN cDNA 2310008H04 gene
	H3143H01	0.0132	LOC436089	Mm.426985	Similar to matrilin 1, cartilage matrix protein
	H3154B03	0.0133	Frap1	Mm.21158	FK506 binding protein 12-rapamycin associated protein 1
	H3084H11	0.0133	Stk40	Mm.41865	Serine/threonine kinase 40
	H3158A04	0.0134	E330013P04Rik	Mm.245813	RIKEN cDNA E330013P04 gene
	H520179	0.0134	Laptm4b	Mm.197518	Lysosomal-associated protein transmembrane 4B
	H3064C11	0.0136	A130040M12Rik	Mm.391766	RIKEN cDNA A130040M12 gene
	H3150F06	0.0136	Fnta	Mm.3496	Farnesyltransferase, CAAX box, alpha
	H3095E04	0.0140	Camk2b	Mm.4857	Calcium/calmodulin-dependent protein kinase II, beta
	H3093F09	0.0140	Gpr98	Mm.288694	G protein-coupled receptor 98
	H3139B10	0.0140	Slk	Mm.281011	STE20-like kinase+E402

	NIA	ρ ≤	Common	Unigene	Name
	H3060H05	0.0141	Cflar	Mm.11778	CASP8 and FADD-like apoptosis regulator
	H552564	0.0142	Xpo7	Mm.152987	Exportin 7
	H3107D10	0.0143	BC038167	Mm.347681	CDNA sequence BC038167
	H3027D05	0.0143	Ly6e	Mm.788	Lymphocyte antigen 6 complex, locus E
	H3065F02	0.0144	data not found	Mm.209805	NOD-derived CD11c +ve dendritic cells cDNA, RIKEN full-length enriched library, clone:F630046O13
	H3124E06	0.0145	Atf4	Mm.641	Activating transcription factor 4
	H572398	0.0146	AI987662	Mm.206911	Expressed sequence AI987662
	H3147B04	0.0146	Fgfr2	Mm.16340	Fibroblast growth factor receptor 2
	H3136E11	0.0146	Nr1d2	Mm.26587	Nuclear receptor subfamily 1, group D, member 2
	H539681	0.0146	Zfp606	Mm.98929	Zinc finger protein 606
	H3147H06	0.0149	Cacybp	Mm.10702	Calcyclin binding protein
	H3103D06	0.0150	Tlk2	Mm.126976	Tousled-like kinase 2
	H420689	0.0151	Butr1	Mm.22540	Butyrophilin related 1
	H3074F11	0.0153	Lsm7	Mm.379101	LSM7 homolog, U6 small nuclear RNA associated
	H3143E11	0.0153	Rab12	Mm.248313	RAB12, member RAS oncogene family
	H3104H04	0.0154	data not found	Mm.430772	Transcribed locus
	H3115F06	0.0155	Mrpl27	Mm.34951	Mitochondrial ribosomal protein L27
	H3006G06	0.0158	Ctsz	Mm.156919	Cathepsin Z
	H400572	0.0158	Pabpc1	Mm.321828	Poly A binding protein, cytoplasmic 1
	H3073E12	0.0158	Rif1	Mm.254530	Rap1 interacting factor 1 homolog
	H3082C12	0.0158	Spp1	Mm.288474	Secreted phosphoprotein 1
	H3108B03	0.0159	H13	Mm.277327	Histocompatibility 13
	H583388	0.0161	Cerk	Mm.222685	Ceramide kinase
	H3142H12	0.0161	data not found	Mm.365349	Transcribed locus
	H604175	0.0161	data not found	Mm.386480	Transcribed locus
	H3100A10	0.0161	Dmxl2	Mm.93636	Dmx-like 2
	H583180	0.0161	Stxbp3a	Mm.316894	Syntaxin binding protein 3A
	H3109F03	0.0162	Bcar1	Mm.3758	Breast cancer anti-estrogen resistance 1
	H3137H10	0.0162	E130310K16Rik	Mm.317473	RIKEN cDNA E130310K16 gene
	H3143G11	0.0162	Pde8b	Mm.100167	Phosphodiesterase 8B
	H3128F04	0.0163	5730406M06Rik	Mm.100117	RIKEN cDNA 5730406M06 gene
	H3150E03	0.0164	4930518F03Rik	Mm.70781	RIKEN cDNA 4930518F03 gene
	H3119D12	0.0164	Osbpl1a	Mm.259470	Oxysterol binding protein-like 1A
	H3141C05	0.0166	Lphn1	Mm.260733	Latrophilin 1
	H598250	0.0167	1700029I01Rik	Mm.392861	RIKEN cDNA 1700029I01 gene
	H3127D11	0.0167	Grid2	Mm.425327	Glutamate receptor, ionotropic, delta 2
	H3008F09	0.0167	Slc25a10	Mm.3991	Solute carrier family 25 (mitochondrial carrier, dicarboxylate transporter), member 10
	H3019A12	0.0168	Hmox1	Mm.276389	Heme oxygenase (decycling) 1
	H3129F07	0.0168	Ptprk	Mm.332303	Protein tyrosine phosphatase, receptor type, K
	H3110D12	0.0168	Ufd1l	Mm.237594	Ubiquitin fusion degradation 1 like

	NIA	ρ ≤	Common	Unigene	Name
	H3092F03	0.0169	Cd276	Mm.5356	CD276 antigen
	H3095H05	0.0170	Pscd3	Mm.281003	Pleckstrin homology, Sec7 and coiled-coil domains 3
	H596106	0.0171	Ahdc1	Mm.31816	AT hook, DNA binding motif, containing 1
	H3158C03	0.0171	Ccdc72	Mm.2395	Coiled-coil domain containing 72
	H643775	0.0171	data not found	Mm.364882	Transcribed locus
	H3121B12	0.0171	Kcnj12	Mm.4970	Potassium inwardly-rectifying channel, subfamily J, member 12
	H637856	0.0171	Rp2h	Mm.288141	Retinitis pigmentosa 2 homolog+E449
	H3027D09	0.0172	Marcksl1	Mm.424974	MARCKS-like 1
	H3081E12	0.0173	Wdr77	Mm.5110	WD repeat domain 77
	H3012F03	0.0175	Glo1	Mm.261984	Glyoxalase 1
	H3024A10	0.0176	Tpp1	Mm.20837	Tripeptidyl peptidase I
	H3026C06	0.0177	Abr	Mm.258939	Active BCR-related gene
	H596699	0.0177	Dhx29	Mm.35094	DEAH (Asp-Glu-Ala-His) box polypeptide 29
	H3120G11	0.0177	Kcnab3	Mm.232472	Potassium voltage-gated channel, shaker-related subfamily, beta member 3
	H3073G12	0.0178	Klhl15	Mm.238117	Kelch-like 15
	H523193	0.0178	Prrg3	Mm.25626	Proline rich Gla (G-carboxyglutamic acid) 3 (transmembrane)
	H3080C05	0.0182	Adam8	Mm.15969	A disintegrin and metallopeptidase domain 8
	H3144F06	0.0182	Adfp	Mm.381	Adipose differentiation related protein
	H3085B11	0.0182	Bola2	Mm.358692	BolA-like 2
	H3157C09	0.0182	Casp6	Mm.281379	Caspase 6
	H3134E05	0.0182	Cntn4	Mm.321683	Contactin 4
	H573537	0.0182	Zdhhc16	Mm.20387	Zinc finger, DHHC domain containing 16
	H657439	0.0183	Mlc1	Mm.32780	Megalencephalic leukoencephalopathy with subcortical cysts 1 homolog
	H3138D11	0.0185	Adam10	Mm.431099	A disintegrin and metallopeptidase domain 10
	H3155B11	0.0185	Chrac1	Mm.23095	Chromatin accessibility complex 1
	H3039G11	0.0185	Exph5	Mm.390155	Exophilin 5
	H3074C02	0.0186	Abhd3	Mm.273108	Abhydrolase domain containing 3
	H3084H06	0.0186	Cdc14b	Mm.25335	CDC14 cell division cycle 14 homolog B
	H3080H03	0.0186	Crip3	Mm.25168	Cysteine-rich protein 3
	H3026F02	0.0186	Gpx1	Mm.1090	Glutathione peroxidase 1
	H3073F05	0.0186	Serbp1	Mm.240490	Serpine1 mRNA binding protein 1
	H598948	0.0188	Cav2	Mm.396075	Caveolin 2
	H617662	0.0188	data not found	Mm.384414	Transcribed locus
	H534171	0.0188	Pxmp3	Mm.132336	Peroxisomal membrane protein 3
	H3126D03	0.0189	Adrbk2	Mm.285619	Adrenergic receptor kinase, beta 2
	H3056D05	0.0189	data not found	Mm.392275	Transcribed locus, weakly similar to XP_921847.1 similar to Retrovirus-related Pol polyprotein (Endonuclease)
	H644827	0.0189	data not found	Mm.393161	Transcribed locus
	H3117G11	0.0189	Eid1	Mm.425101	EP300 interacting inhibitor of differentiation 1
	H3153D01	0.0190	2210010A19Rik	Mm.171484	RIKEN cDNA 2210010A19 gene
	H3096D02	0.0191	Fat4	Mm.316210	FAT tumor suppressor homolog 4+E482

	NIA	ρ ≤	Common	Unigene	Name
	H3111A06	0.0192	Rpl22	Mm.426317	Ribosomal protein L22
	H3107F05	0.0192	Uqcrb	Mm.379136	Ubiquinol-cytochrome c reductase binding protein
	H3044H12	0.0193	Lace1	Mm.89646	Lactation elevated 1
	H3132F05	0.0193	Nsbp1	Mm.298443	Nucleosome binding protein 1
	H3143F12	0.0194	Ctps2	Mm.2065	Cytidine 5'-triphosphate synthase 2
	H493659	0.0194	Srp19	Mm.29452	Signal recognition particle 19
	H3024B07	0.0196	Igf2	Mm.3862	Insulin-like growth factor 2
	H3047B12	0.0199	Akap7	Mm.179069	A kinase (PRKA) anchor protein 7
	H3107F11	0.0200	Dusp3	Mm.392115	Dual specificity phosphatase 3 (vaccinia virus phosphatase VH1-related)
	H331202	0.0200	Dync1i1	Mm.20893	Dynein cytoplasmic 1 intermediate chain 1
	H3115H06	0.0200	Ftl1	Mm.30357	Ferritin light chain 1
	H3134C05	0.0200	Mgp	Mm.243085	Matrix Gla protein
	H3093B05	0.0200	Rbm26	Mm.291542	RNA binding motif protein 26
	H658624	0.0203	1110038F14Rik	Mm.322968	RIKEN cDNA 1110038F14 gene
	H3158A10	0.0203	Abi2	Mm.212066	Abl-interactor 2
	H555877	0.0203	Adamts10	Mm.29304	A disintegrin-like and metallopeptidase (reprolysin type) with thrombospondin type 1 motif, 10
	H3141B11	0.0203	H2-K1	Mm.33263	Histocompatibility 2, K1, K region
	H3158G12	0.0203	Mtl5	Mm.209941	Metallothionein-like 5, testis-specific (tesmin)
	H3155C06	0.0204	Cd24a	Mm.29742	CD24a antigen
	H3120D06	0.0204	Wdr6	Mm.335454	WD repeat domain 6
	H3049H05	0.0206	Amot	Mm.100068	Angiomotin
	H481408	0.0206	Cox8a	Mm.14022	Cytochrome c oxidase, subunit VIIIa
	H3072B06	0.0207	data not found	Mm.383962	Transcribed locus
	H3118D04	0.0207	Slc29a1	Mm.29744	Solute carrier family 29 (nucleoside transporters), member 1
	H3136D02	0.0207	Sorbs2	Mm.211096	Sorbin and SH3 domain containing 2
	H3135H04	0.0209	Tst	Mm.15312	Thiosulfate sulfurtransferase, mitochondrial
	H3155G10	0.0210	Myo1b	Mm.3390	Myosin IB
	H3140D10	0.0211	Dgcr2	Mm.254515	DiGeorge syndrome critical region gene 2
	H3151E09	0.0213	2010109N14Rik	Mm.260791	RIKEN cDNA 2010109N14 gene
	H555816	0.0216	Tm6sf1	Mm.221412	Transmembrane 6 superfamily member 1
	H3152D06	0.0217	Alas2	Mm.302724	Aminolevulinic acid synthase 2, erythroid
	H3118D05	0.0217	Rps25	Mm.292027	Ribosomal protein S25
	H314426	0.0218	Cbr1	Mm.26940	Carbonyl reductase 1
	H3065E09	0.0219	Eftud1	Mm.238020	Elongation factor Tu GTP binding domain containing 1
	H3145D11	0.0219	Rps24	Mm.16775	Ribosomal protein S24
	H596829	0.0219	Tacc1	Mm.308452	Transforming, acidic coiled-coil containing protein 1
	H641371	0.0223	BC018507	Mm.139738	CDNA sequence BC018507
	H642164	0.0223	Gtf2f2	Mm.20415	General transcription factor IIF, polypeptide 2
	H3104F03	0.0223	Krt18	Mm.22479	Keratin 18
	H635173	0.0223	Slc3a2	Mm.4114	Solute carrier family 3 (activators of dibasic and neutral amino acid transport), member 2

	NIA	ρ ≤	Common	Unigene	Name
	H3124G03	0.0224	1810007M14Rik	Mm.347	RIKEN cDNA 1810007M14 gene
	H3142H05	0.0225	Tmem147	Mm.27499	Transmembrane protein 147
	H621131	0.0227	1700020O03Rik	Mm.252967	RIKEN cDNA 1700020O03 gene
	H477897	0.0228	Epb4.1l1	Mm.20852	Erythrocyte protein band 4.1-like 1
‡	H3083C05	0.0228	Sp3	Mm.124328	Trans-acting transcription factor 3
	H3057D12	0.0229	Rnf170	Mm.291906	Ring finger protein 170
	H3142E03	0.0232	Hdac3	Mm.20521	Histone deacetylase 3
	H3066F01	0.0232	Rgs2	Mm.28262	Regulator of G-protein signaling 2
	H3097B09	0.0233	Cd97	Mm.334648	CD97 antigen
	H3040F11	0.0233	Ifi30	Mm.30241	Interferon gamma inducible protein 30
	H351947	0.0233	Lrrn1	Mm.428543	Leucine rich repeat protein 1, neuronal
	H3123F05	0.0233	Spint2	Mm.295230	Serine protease inhibitor, Kunitz type 2
	H595923	0.0233	Yy1	Mm.3868	YY1 transcription factor
	H3114B11	0.0234	Rps6ka3	Mm.328476	Ribosomal protein S6 kinase polypeptide 3
	H316130	0.0235	Blmh	Mm.399785	Bleomycin hydrolase
	H3124H05	0.0235	Inadl	Mm.90218	InaD-like+E538
	H3113F06	0.0235	Rnh1	Mm.279485	Ribonuclease/angiogenin inhibitor 1
	H3109B08	0.0236	Slc15a4	Mm.28506	Solute carrier family 15, member 4
	H635726	0.0239	Itih2	Mm.182043	Inter-alpha trypsin inhibitor, heavy chain 2
	H3047D04	0.0243	Ccdc86	Mm.294908	Coiled-coil domain containing 86
	H482752	0.0243	Icosl	Mm.17819	Icos ligand
	H400664	0.0245	Gng11	Mm.25547	Guanine nucleotide binding protein (G protein), gamma 11
	H3075C08	0.0245	Klf4	Mm.4325	Kruppel-like factor 4 (gut)
	H3111F12	0.0248	Cnot6	Mm.247113	CCR4-NOT transcription complex, subunit 6
	H3038H11	0.0248	EG210583	Mm.327168	Predicted gene, EG210583
	H519072	0.0248	Xpr1	Mm.266215	Xenotropic and polytropic retrovirus receptor 1
	H597636	0.0250	data not found	Mm.386223	Transcribed locus
	H3031C12	0.0251	Ndrg1	Mm.30837	N-myc downstream regulated gene 1
	H3157B11	0.0252	Cbx5	Mm.262059	Chromobox homolog 5
	H3119D11	0.0252	Ifitm2	Mm.379266	Interferon induced transmembrane protein 2
	H3106B12	0.0252	LOC434459	Mm.359230	Similar to RIKEN cDNA 4930503E14
	H575724	0.0252	Nxf1	Mm.7271	Nuclear RNA export factor 1 homolog
‡	H3118B10	0.0252	Ogt	Mm.259191	O-linked N-acetylglucosamine (GlcNAc) transferase+E555
	H3101F06	0.0252	Pum1	Mm.34701	Pumilio 1
	H3120F05	0.0252	Rapgef1	Mm.298274	Rap guanine nucleotide exchange factor (GEF) 1
	H3008E04	0.0252	Rps20	Mm.21938	Ribosomal protein S20
	H3003A10	0.0252	Tpbpb	Mm.30144	Trophoblast specific protein beta
	H579505	0.0254	Chic1	Mm.42223	Cysteine-rich hydrophobic domain 1
	H3091E12	0.0254	data not found	Mm.432813	Adult male liver tumor cDNA, RIKEN full-length enriched library, clone:C730024P13+E561
	H3104A05	0.0254	Gprk5	Mm.279400	G protein-coupled receptor kinase 5

	NIA	ρ ≤	Common	Unigene	Name
	H3143C12	0.0254	Mov10	Mm.1597	Moloney leukemia virus 10
	H3019F11	0.0254	Ssu72	Mm.294770	Ssu72 RNA polymerase II CTD phosphatase homolog
	H722882	0.0254	Tgfbi	Mm.14455	Transforming growth factor, beta induced
	H620826	0.0256	2810423E13Rik	Mm.130610	RIKEN cDNA 2810423E13 gene
	H3116G03	0.0256	Lasp1	Mm.271967	LIM and SH3 protein 1
	H386989	0.0256	Spr	Mm.28393	Sepiapterin reductase
	H556151	0.0256	Zmym3	Mm.23458	Zinc finger, MYM-type 3
	H3117E06	0.0257	D330038O06Rik	Mm.41364	RIKEN cDNA D330038O06 gene
	H3071C09	0.0257	Mkln1	Mm.238038	Muskelin 1, intracellular mediator containing kelch motifs
	H3105F04	0.0258	data not found	Mm.395177	Transcribed locus
	H3109D02	0.0259	4930504E06Rik	Mm.279287	RIKEN cDNA 4930504E06 gene
	H3159B11	0.0261	Gng12	Mm.234342	Guanine nucleotide binding protein (G protein), gamma 12
	H3116H05	0.0261	Pias1	Mm.306663	Protein inhibitor of activated STAT 1
	H3132C05	0.0261	Rasa3	Mm.18517	RAS p21 protein activator 3
	H3147H05	0.0261	Slc6a6	Mm.395650	Solute carrier family 6 (neurotransmitter transporter, taurine), member 6
	H641049	0.0261	Thap2	Mm.32314	THAP domain containing, apoptosis associated protein 2
	H3133C12	0.0261	Xpnpep3	Mm.410525	X-prolyl aminopeptidase (aminopeptidase P) 3, putative
	H349037	0.0262	Yipf1	Mm.29118	Yip1 domain family, member 1
	H538045	0.0263	Apoc1	Mm.182440	Apolipoprotein C-I
	H3135A05	0.0264	Hmga2	Mm.157190	High mobility group AT-hook 2
	H3002D12	0.0264	Surf6	Mm.245577	Surfeit gene 6
	H3142G05	0.0264	Tep1	Mm.318736	Telomerase associated protein 1
	H580033	0.0265	2310008H09Rik	Mm.334185	RIKEN cDNA 2310008H09 gene
	H3156C06	0.0265	Acvr2a	Mm.314338	Activin receptor IIA
	H3123E06	0.0265	Ythdf3	Mm.23834	YTH domain family 3
	H3122G03	0.0267	2010003J03Rik	Mm.299167	RIKEN cDNA 2010003J03 gene
	H3127G05	0.0267	Ppt1	Mm.277719	Palmitoyl-protein thioesterase 1
	H3080H12	0.0268	Cry1	Mm.26237	Cryptochrome 1 (photolyase-like)
	H3146F08	0.0269	Usp28	Mm.21630	Ubiquitin specific peptidase 28
	H3139C09	0.0270	D6Wsu116e	Mm.28524	DNA segment, Chr 6, Wayne State University 116, expressed
	H3148H03	0.0270	Xpo4	Mm.202747	Exportin 4
	H3144H10	0.0272	Smarcc1	Mm.85410	SWI/SNF related, matrix associated, actin dependent regulator of chromatin, subfamily c, member 1
	H721683	0.0274	Ubl3	Mm.21846	Ubiquitin-like 3
	H3081D05	0.0275	1190005F20Rik	Mm.289373	RIKEN cDNA 1190005F20 gene
	H400580	0.0275	Etv5	Mm.155708	Ets variant gene 5
	H574982	0.0279	C330019G07Rik	Mm.335866	RIKEN cDNA C330019G07 gene
	H3036E10	0.0279	Cugbp2	Mm.398543	CUG triplet repeat, RNA binding protein 2
	H373295	0.0281	Copg2as2	Mm.22891	Coatomer protein complex, subunit gamma 2, antisense 2
	H444424	0.0286	Ddit3	Mm.110220	DNA-damage inducible transcript 3
	H637576	0.0286	Phtf2	Mm.86410	Putative homeodomain transcription factor 2

	NIA	ρ ≤	Common	Unigene	Name
	H3140G01	0.0286	Timeless	Mm.6458	Timeless homolog
	H3120A04	0.0287	Cpne8	Mm.290991	Copine VIII
	H599126	0.0287	Myo9a	Mm.249545	Myosin IXa
	H385200	0.0289	Itm2c	Mm.29870	Integral membrane protein 2C
	H3066F04	0.0292	Igfbp7	Mm.157655	Insulin-like growth factor binding protein 7
	H3147B05	0.0293	Smu1	Mm.289929	Smu-1 suppressor of mec-8 and unc-52 homolog
	H617765	0.0295	data not found	Mm.385334	Transcribed locus
	H3138B04	0.0296	Arhgef17	Mm.29954	Rho guanine nucleotide exchange factor (GEF) 17
	H615477	0.0296	D10Ertd610e	Mm.272230	DNA segment, Chr 10, ERATO Doi 610, expressed
	H3118F07	0.0296	Olfm1	Mm.43278	Olfactomedin 1
	H3122C06	0.0296	Spast	Mm.19804	Spastin
	H3056F03	0.0296	Tmem49	Mm.390398	Transmembrane protein 49
	H3006C12	0.0297	Ctr9	Mm.255858	Ctr9, Paf1/RNA polymerase II complex component, homolog
	H3061F05	0.0297	Nfkb1	Mm.256765	Nuclear factor of kappa light chain gene enhancer in B-cells 1, p105
	H3140D11	0.0300	5031439G07Rik	Mm.323925	RIKEN cDNA 5031439G07 gene
	H583512	0.0300	Spna2	Mm.204969	Spectrin alpha 2
	H3133F10	0.0302	Nr2c2	Mm.383196	Nuclear receptor subfamily 2, group C, member 2
	H3119A11	0.0303	Eif4g3	Mm.268903	Eukaryotic translation initiation factor 4 gamma, 3
	H3096H06	0.0303	Hps4	Mm.238043	Hermansky-Pudlak syndrome 4 homolog+E621
	H573453	0.0303	Rsf1	Mm.211743	Remodeling and spacing factor 1
	H635679	0.0306	Tgm2	Mm.330731	Transglutaminase 2, C polypeptide
	H597548	0.0308	Fbxl11	Mm.31941	F-box and leucine-rich repeat protein 11
	H3149F06	0.0309	Chd4	Mm.333388	Chromodomain helicase DNA binding protein 4
	H3044F11	0.0309	Mtap4	Mm.217318	Microtubule-associated protein 4
	H313276	0.0309	Rpl12	Mm.250030	Ribosomal protein L12
	H3136H09	0.0309	Sfxn3	Mm.36169	Sideroflexin 3
	H3023B02	0.0310	Vps13b	Mm.309250	Vacuolar protein sorting 13B+E766
	H406857	0.0311	Trak1	Mm.305318	Trafficking protein, kinesin binding 1
	H608716	0.0311	Ucp2	Mm.171378	Uncoupling protein 2 (mitochondrial, proton carrier)
	H3133F12	0.0312	Ccnd2	Mm.333406	Cyclin D2
	H3107G05	0.0312	Gm323	Mm.391006	Gene model 323, (NCBI)
	H577341	0.0312	Sri	Mm.96211	Sorcin
	H3048B01	0.0313	Fubp1	Mm.278922	Far upstream element (FUSE) binding protein 1
	H3139H05	0.0313	Rps23	Mm.389421	Ribosomal protein S23
	H3022B11	0.0314	9230105E10Rik	Mm.425502	RIKEN cDNA 9230105E10 gene
	H638093	0.0314	Ercc3	Mm.282335	Excision repair cross-complementing rodent repair deficiency, complementation group 3
	H3151D03	0.0318	Kif1b	Mm.402393	Kinesin family member 1B
	H3093C06	0.0319	B3gntl1	Mm.386847	UDP-GlcNAc:betaGal beta-1,3-N-acetylglucosaminyltransferase-like 1
	H642610	0.0319	Trim27	Mm.32492	Tripartite motif protein 27
	H658857	0.0322	2410091C18Rik	Mm.104959	RIKEN cDNA 2410091C18 gene

	NIA	ρ ≤	Common	Unigene	Name
	H3128G05	0.0322	Aff1	Mm.6949	AF4/FMR2 family, member 1
	H3001D03	0.0324	AI428936	Mm.227325	Expressed sequence AI428936
	H3140D05	0.0324	Enpp5	Mm.30145	Ectonucleotide pyrophosphatase/phosphodiesterase 5
	H3133D04	0.0324	Ndufv2	Mm.2206	NADH dehydrogenase (ubiquinone) flavoprotein 2
	H3012D05	0.0324	Yeats4	Mm.233529	YEATS domain containing 4
	H3144F11	0.0326	Matr3	Mm.215034	Matrin 3
	H3022B06	0.0326	Triobp	Mm.123714	TRIO and F-actin binding protein
	H3144A03	0.0327	2210010B09Rik	Mm.86627	RIKEN cDNA 2210010B09 gene
	H3118G11	0.0327	Klhl23	Mm.138073	Kelch-like 23
	H3096H05	0.0327	Stag1	Mm.42135	Stromal antigen 1
	H3150E11	0.0328	AW822216	Mm.236454	Expressed sequence AW822216
	H3112F12	0.0329	1810035L17Rik	Mm.355701	RIKEN cDNA 1810035L17 gene
	H3085C06	0.0329	D17Ertd657e	Mm.372318	DNA segment, Chr 17, ERATO Doi 657, expressed
	H662272	0.0330	Exoc3	Mm.261859	Exocyst complex component 3
	H596223	0.0331	data not found	Mm.262757	Transcribed locus
	H636050	0.0331	data not found	Mm.32452	Transcribed locus
	H3131B11	0.0331	Glg1	Mm.276271	Golgi apparatus protein 1
	H575876	0.0333	data not found	Mm.392224	Transcribed locus
	H332401	0.0333	Dchs1	Mm.334108	Dachsous 1+E661
	H599174	0.0335	Macf1	Mm.402299	Microtubule-actin crosslinking factor 1
	H3102C12	0.0336	Arl6ip1	Mm.29924	ADP-ribosylation factor-like 6 interacting protein 1
	H537167	0.0336	Ttc14	Mm.275710	Tetratricopeptide repeat domain 14
	H3150E05	0.0337	Rai12	Mm.13430	Retinoic acid induced 12
	H3119A08	0.0338	Clic1	Mm.29524	Chloride intracellular channel 1
	H582992	0.0340	Dleu2	Mm.32886	Deleted in lymphocytic leukemia, 2
	H3128C05	0.0341	6720457D02Rik	Mm.343450	RIKEN cDNA 6720457D02 gene
	H3155H03	0.0342	Rpl3	Mm.290771	Ribosomal protein L3
	H3043G10	0.0344	Snrpa1	Mm.22362	Small nuclear ribonucleoprotein polypeptide A'
	H3012C11	0.0345	2900026A02Rik	Mm.160131	RIKEN cDNA 2900026A02 gene
	H3056C10	0.0345	Arid2	Mm.17166	AT rich interactive domain 2 (Arid-rfx like)
	H3055H05	0.0345	Carhsp1	Mm.142095	Calcium regulated heat stable protein 1
	H3115G11	0.0345	Evl	Mm.238841	Ena-vasodilator stimulated phosphoprotein
	H3129E12	0.0345	Gorasp2	Mm.271950	Golgi reassembly stacking protein 2
	H3097B12	0.0345	Nt5c2	Mm.248652	5'-nucleotidase, cytosolic II
	H3125H06	0.0345	Rpl4	Mm.280083	Ribosomal protein L4
	H3135F12	0.0346	Crebl1	Mm.4068	CAMP responsive element binding protein-like 1
	H3077D10	0.0347	BC026590	Mm.268148	CDNA sequence BC026590
	H573864	0.0348	Gm826	Mm.291144	Gene model 826, (NCBI)
	H3124F10	0.0349	Pxmp2	Mm.21853	Peroxisomal membrane protein 2
	H3105F06	0.0351	2310039H08Rik	Mm.358702	RIKEN cDNA 2310039H08 gene

	NIA	ρ ≤	Common	Unigene	Name
	H3143B11	0.0351	Ccdc124	Mm.295791	Coiled-coil domain containing 124
	H3076D05	0.0351	Lrrc50	Mm.432018	Leucine rich repeat containing 50
	H3141H11	0.0352	Amhr2	Mm.60331	Anti-Mullerian hormone type 2 receptor
	H3106E05	0.0355	data not found	Mm.272185	Membrane-associated ring finger (C3HC4) 6
	H637060	0.0356	Bclaf1	Mm.294783	BCL2-associated transcription factor 1
	H635134	0.0357	Htr7	Mm.254266	5-hydroxytryptamine (serotonin) receptor 7
	H3103G06	0.0358	Rplp2	Mm.432008	Ribosomal protein, large P2
	H3146C12	0.0359	Lrp1	Mm.271854	Low density lipoprotein receptor-related protein 1
	H3044E06	0.0359	Psme1	Mm.830	Proteasome (prosome, macropain) 28 subunit, alpha
	H3107A11	0.0364	Abl1	Mm.1318	V-abl Abelson murine leukemia oncogene 1
	H3138H10	0.0364	Ctsd	Mm.231395	Cathepsin D
	H3123B01	0.0367	6720475J19Rik	Mm.273536	RIKEN cDNA 6720475J19 gene
	H3034C12	0.0369	Arhgap29	Mm.229287	Rho GTPase activating protein 29
	H3131F02	0.0370	2310045N01Rik	Mm.644	RIKEN cDNA 2310045N01 gene
	H3116E12	0.0370	Ceecam1	Mm.296336	Cerebral endothelial cell adhesion molecule 1
	H3117F02	0.0370	Pde1a	Mm.40678	Phosphodiesterase 1A, calmodulin-dependent
	H3103D05	0.0372	Cth	Mm.28301	Cystathionase (cystathionine gamma-lyase)
	H3129H03	0.0373	Hadh	Mm.260164	Hydroxyacyl-Coenzyme A dehydrogenase
	H403277	0.0374	R3hcc1	Mm.27738	R3H domain and coiled-coil containing 1
	H3051B09	0.0374	Stxbp6	Mm.285400	Syntaxin binding protein 6 (amisyn)
	H533282	0.0378	Actr10	Mm.29317	ARP10 actin-related protein 10 homolog
	H3019A03	0.0380	Jtb	Mm.13912	Jumping translocation breakpoint
	H618846	0.0380	Zdhhc15	Mm.30574	Zinc finger, DHHC domain containing 15
	H3147E11	0.0382	Ptprf	Mm.29855	Protein tyrosine phosphatase, receptor type, F
	H721890	0.0382	Selpl	Mm.332590	Selectin, platelet (p-selectin) ligand
	H3153F11	0.0386	Sltm	Mm.22379	SAFB-like, transcription modulator
	H3041A06	0.0387	Srpk2	Mm.288728	Serine/arginine-rich protein specific kinase 2
	H3147C04	0.0388	Lamb1-1	Mm.172674	Laminin B1 subunit 1
	H3038H04	0.0388	Pnpla8	Mm.54126	Patatin-like phospholipase domain containing 8
	H3004E01	0.0388	Tmem68	Mm.212266	Transmembrane protein 68
	H3087E01	0.0389	Anxa4	Mm.259702	Annexin A4
	H3067D10	0.0391	BC014805	Mm.207060	CDNA sequence BC014805
	H3096D05	0.0391	Nipbl	Mm.240329	Nipped-B homolog
	H3046H10	0.0391	Upf3b	Mm.271160	UPF3 regulator of nonsense transcripts homolog B
	H3074D03	0.0392	5730403B10Rik	Mm.157648	RIKEN cDNA 5730403B10 gene
	H366995	0.0392	H2-D1	Mm.195061	Histocompatibility 2, D region locus 1
	H3049F05	0.0392	Rae1	Mm.4113	RAE1 RNA export 1 homolog
	H3044B02	0.0394	Slc2a3	Mm.395108	Solute carrier family 2 (facilitated glucose transporter), member 3
	H3146H06	0.0395	Slc44a2	Mm.148425	Solute carrier family 44, member 2
	H642492	0.0396	Asxl3	Mm.392310	Additional sex combs like 3

	NIA	ρ ≤	Common	Unigene	Name
	H3004C12	0.0396	Nt5c3l	Mm.28738	5'-nucleotidase, cytosolic III-like
	H348447	0.0400	Zfp324	Mm.133086	Zinc finger protein 324
	H3010D11	0.0401	D19Wsu162e	Mm.329895	DNA segment, Chr 19, Wayne State University 162, expressed
	H3099F03	0.0401	Prei4	Mm.211211	Preimplantation protein 4
	H3032E12	0.0401	Rpl7	Mm.379004	Ribosomal protein L7
	H3132A04	0.0402	Fis1	Mm.25849	Fission 1 (mitochondrial outer membrane) homolog+E728
	H3134C12	0.0403	Ocrl	Mm.210343	Oculocerebrorenal syndrome of Lowe
	H3015C04	0.0404	0610038F07Rik	Mm.180063	RIKEN cDNA 0610038F07 gene
	H3074E12	0.0404	Usp47	Mm.16974	Ubiquitin specific peptidase 47
	H3089H08	0.0408	2600011C06Rik	Mm.46005	RIKEN cDNA 2600011C06 gene
	H3016D03	0.0409	Stk10	Mm.8235	Serine/threonine kinase 10
	H3155H09	0.0410	Ikbke	Mm.386783	Inhibitor of kappaB kinase epsilon
	H3139G12	0.0410	Prkcm	Mm.133719	Protein kinase C, mu
	H3061D03	0.0411	Pcsk5	Mm.3401	Proprotein convertase subtilisin/kexin type 5
	H3062H04	0.0411	Utx	Mm.257498	Ubiquitously transcribed tetratricopeptide repeat gene, X chromosome
	H3137B11	0.0412	Mest	Mm.335639	Mesoderm specific transcript
	H3107F06	0.0412	Smarca2	Mm.313303	SWI/SNF related, matrix associated, actin dependent regulator of chromatin, subfamily a, member 2
‡	H3123B05	0.0413	Hdac2	Mm.19806	Histone deacetylase 2
	H3146H05	0.0413	Taf5l	Mm.291777	TAF5-like RNA polymerase II, p300/CBP-associated factor (PCAF)-associated factor
	H3039H10	0.0414	Lima1	Mm.33207	LIM domain and actin binding 1
	H3029D12	0.0415	2610507B11Rik	Mm.237103	RIKEN cDNA 2610507B11 gene
	H3010G06	0.0415	Capg	Mm.18626	Capping protein (actin filament), gelsolin-like
	H3032B05	0.0416	Eif3s6	Mm.289992	Eukaryotic translation initiation factor 3, subunit 6
	H400025	0.0416	Grin1	Mm.278672	Glutamate receptor, ionotropic, NMDA1 (zeta 1)
	H372403	0.0417	Cnnm3	Mm.256323	Cyclin M3
	H3675547	0.0418	Tnfrsf1a	Mm.1258	Tumor necrosis factor receptor superfamily, member 1a
	H3099H11	0.0419	Myo1d	Mm.151948	Myosin ID
	H3076C07	0.0419	Slc35f2	Mm.26159	Solute carrier family 35, member F2
	H3055E11	0.0421	Dtl	Mm.189102	Denticleless homolog
	H3142D02	0.0421	Usf1	Mm.8	Upstream transcription factor 1
	H3133F04	0.0422	Snrpd2	Mm.29135	Small nuclear ribonucleoprotein D2
	H3064B04	0.0426	Apobec4	Mm.158650	Apolipoprotein B mRNA editing enzyme, catalytic polypeptide-like 4
	H3113F05	0.0427	Fxyd6	Mm.208287	FXYD domain-containing ion transport regulator 6
	H3113H05	0.0427	Snrpe	Mm.249110	Small nuclear ribonucleoprotein E
	H582903	0.0427	Tpr	Mm.174256	Translocated promoter region
	H3112B09	0.0428	Ankrd10	Mm.12459	Ankyrin repeat domain 10
	H3096G05	0.0430	BC048546	Mm.259234	CDNA sequence BC048546
	H581127	0.0431	Uqcrfs1	Mm.181933	Ubiquinol-cytochrome c reductase, Rieske iron-sulfur polypeptide 1
	H3093H10	0.0432	EG627488	Mm.339215	Predicted gene, EG627488
	H3124G02	0.0436	5730405I09Rik	Mm.376607	RIKEN cDNA 5730405I09 gene

	NIA	ρ ≤	Common	Unigene	Name
	H3156H04	0.0436	Atp5g3	Mm.2966	ATP synthase, H+ transporting, mitochondrial F0 complex, subunit c (subunit 9), isoform 3
	H400429	0.0436	Hoxd9	Mm.26544	Homeo box D9
	H3097F04	0.0437	5730469M10Rik	Mm.27227	RIKEN cDNA 5730469M10 gene
	H406320	0.0437	Cx3cl1	Mm.103711	Chemokine (C-X3-C motif) ligand 1
	H3005D10	0.0437	Sema3e	Mm.134093	Sema domain, immunoglobulin domain (Ig), short basic domain, secreted, (semaphorin) 3E
	H3091B12	0.0440	Lrp6	Mm.321990	Low density lipoprotein receptor-related protein 6
	H3021F03	0.0440	Tomm70a	Mm.213292	Translocase of outer mitochondrial membrane 70 homolog A
	H3110F02	0.0441	Cct7	Mm.289900	Chaperonin subunit 7 (eta)
	H619811	0.0443	5830418K08Rik	Mm.218317	RIKEN cDNA 5830418K08 gene
	H3146A05	0.0444	4732435N03Rik	Mm.334569	RIKEN cDNA 4732435N03 gene
	H3008D04	0.0444	Gtf3c1	Mm.294173	General transcription factor III C 1
	H3150H09	0.0444	Wac	Mm.272685	WW domain containing adaptor with coiled-coil
	H3089H09	0.0446	Ern1	Mm.340943	Endoplasmic reticulum (ER) to nucleus signalling 1
	H722038	0.0448	Ilf2	Mm.272336	Interleukin enhancer binding factor 2
	H3109D12	0.0452	Kctd4	Mm.390712	Potassium channel tetramerisation domain containing 4
	H3081E06	0.0452	Spty2d1	Mm.155687	SPT2, Suppressor of Ty, domain containing 1
	H3122D12	0.0455	1700021C14Rik	Mm.273330	RIKEN cDNA 1700021C14 gene
	H636029	0.0455	data not found	Mm.83400	Transcribed locus
	H3156C03	0.0455	Ppm1d	Mm.45609	Protein phosphatase 1D magnesium-dependent, delta isoform
	H3140E06	0.0457	Cd151	Mm.30246	CD151 antigen
	H599227	0.0459	Sh3kbp1	Mm.286495	SH3-domain kinase binding protein 1
	H312860	0.0459	Tcea3	Mm.112	Transcription elongation factor A (SII), 3
	H3141A06	0.0460	Dgkq	Mm.260921	Diacylglycerol kinase, theta
	H425532	0.0462	Cic	Mm.28833	Capicua homolog+E786
	H3138G08	0.0462	Slc25a37	Mm.293635	Solute carrier family 25, member 37
	H580104	0.0464	data not found	Mm.395447	Transcribed locus
	H351781	0.0466	2610301G19Rik	Mm.218284	RIKEN cDNA 2610301G19 gene
	H636074	0.0468	1110019K23Rik	Mm.153030	RIKEN cDNA 1110019K23 gene
	H3100C09	0.0468	Ankhd1	Mm.24790	Ankyrin repeat and KH domain containing 1
	H336003	0.0468	Zfyve20	Mm.290734	Zinc finger, FYVE domain containing 20
	H333933	0.0469	Cd300lf	Mm.277387	CD300 antigen like family member F
	H3064C06	0.0469	Gldc	Mm.274852	Glycine decarboxylase
	H652862	0.0469	Mbd4	Mm.259308	Methyl-CpG binding domain protein 4
	H329500	0.0469	Ugcgl1	Mm.392153	UDP-glucose ceramide glucosyltransferase-like 1
	H3131F12	0.0470	Stfa3	Mm.327618	Stefin A3
	H3063B04	0.0473	Abpb	Mm.110829	Androgen binding protein beta
	H3135D12	0.0473	data not found	Mm.38450	Septin 9
	H583622	0.0474	AI450540	Mm.358870	Expressed sequence AI450540
	H3108C10	0.0474	Mylk	Mm.33360	Myosin, light polypeptide kinase
	H3013H11	0.0474	Ranbp1	Mm.3752	RAN binding protein 1

	NIA	ρ ≤	Common	Unigene	Name
	H3151E11	0.0474	Shc1	Mm.86595	Src homology 2 domain-containing transforming protein C1
	H331592	0.0475	data not found	Mm.385639	Transcribed locus
	H3018B03	0.0478	Mrpl12	Mm.133851	Mitochondrial ribosomal protein L12
	H3013F11	0.0478	Rest	Mm.392264	RE1-silencing transcription factor
	H3079B11	0.0478	Surb7	Mm.26212	SRB7 (suppressor of RNA polymerase B) homolog
	H477860	0.0478	Tbx2	Mm.287052	T-box 2
	H583371	0.0484	Mical1	Mm.290431	Microtubule associated monoxygenase, calponin and LIM domain containing 1
	H3078H08	0.0486	AU020206	Mm.200422	Expressed sequence AU020206
	H3089E05	0.0486	Etfdh	Mm.28336	Electron transferring flavoprotein, dehydrogenase
	H3143A06	0.0486	Gnb2	Mm.30141	Guanine nucleotide binding protein, beta 2
	H3145F12	0.0487	2410002F23Rik	Mm.274492	RIKEN cDNA 2410002F23 gene
	H3144D10	0.0489	Col18a1	Mm.4352	Procollagen, type XVIII, alpha 1
	H3143C11	0.0493	Pan3	Mm.320469	PAN3 polyA specific ribonuclease subunit homolog
	H3144E04	0.0493	Pnrc2	Mm.29159	Proline-rich nuclear receptor coactivator 2
	H313871	0.0493	Ptpn14	Mm.4498	Protein tyrosine phosphatase, non-receptor type 14
	H721451	0.0493	Trim10	Mm.299155	Tripartite motif protein 10
	H557009	0.0493	Ywhaz	Mm.3360	Tyrosine 3-monooxygenase/tryptophan 5-monooxygenase activation protein, zeta polypeptide
	H3146H04	0.0495	Fcho2	Mm.23928	FCH domain only 2
	H3096D12	0.0496	5730403M16Rik	Mm.23838	RIKEN cDNA 5730403M16 gene
	H576800	0.0496	data not found	Mm.30468	Transcribed locus
	H3155F12	0.0496	Eif3s3	Mm.289800	Eukaryotic translation initiation factor 3, subunit 3 (gamma)
	H3135H11	0.0496	Map1lc3a	Mm.196239	Microtubule-associated protein 1 light chain 3 alpha
	H3092E05	0.0497	Aco2	Mm.387042	Aconitase 2, mitochondrial
	H3149B05	0.0497	Taf12	Mm.331926	TAF12 RNA polymerase II, TATA box binding protein (TBP)-associated factor
	H3084G06	0.0498	Prpf4b	Mm.10027	PRP4 pre-mRNA processing factor 4 homolog B
	H3044A03	0.0500	Crtap	Mm.20904	Cartilage associated protein
‡	Detected in SAGE analysis				
