# Supplementary material for: Ovarian gene expression in the absence of FIGLA, an oocyte-specific transcription factor
Source: BMC Dev Biol. 2007 Jun 13;7:67. doi: 10.1186/1471-213X-7-67 (PMC1906760; doi:10.1186/1471-213X-7-67)
Supplement: Additional file 7 — SAGE libraries: genes potentially up-regulated by FIGLA [file 1471-213X-7-67-S7.pdf]

Additional file 7 – SAGE libraries: genes potentially up-regulated by FIGLA

	SAGE Tag	ρ ≤	Common	Genbank	Unigene	Normal Tags	Null Tags
	ATCTTGTGAA	0.0000	Rps2	NM_008503	Mm.299566	121	0
	TACAAATGTG	0.0000	Eef1a1	BC040689	Mm.335315	50	0
	ATGTTTTGTC	0.0000	Mrps24	NM_026080	Mm.7328	28	0
►	GTTGTGCATC	0.0000	Dppa3	NM_139218	Mm.27982	28	0
	ATTTCGGGAA	0.0000	Ube2d3	data not found	Mm.49884	27	0
►	AGGAGCCTGG	0.0000	Pou5f1	NM_013633	Mm.17031	26	0
	ACCTCTTAGG	0.0000	Gtl2	data not found	Mm.289645	26	0
►	CTGTGGCTGG	0.0000		AK087784	Mm.31556	23	0
	AGATAGGCCC	0.0000	Col1a1	data not found	Mm.277735	22	0
	TCCTCAGCAT	0.0000	data not found	data not found	Mm.387484, Mm.310479	22	0
	TGACCCCCTC	0.0000	multiple annotation			22	0
	ATGGATGCCC	0.0000	Paox	BC033913	Mm.44197	21	0
	ATTATAAAAA	0.0000	Nol7	NM_023554	Mm.279041	21	0
►	ACATACAGTA	0.0000		2410146L05Rik	Mm.26883	21	0
	ATTGTTTGTG	0.0000	Clta	NM_016760	Mm.298875	20	0
	ATGAGGCTCT	0.0000	Leprel2	data not found	Mm.35708	20	0
	ATAAATGAAA	0.0000	data not found	NM_028030	Mm.29148	18	0
	TTGTTACGGA	0.0000	data not found	BE199211	Mm.296346	17	0
	CAGTTGCCCC	0.0000	data not found	data not found		17	0
	AAGGAAGAGG	0.0000	Sp3	BC027797	Mm.124328	16	0
	ATAGAATAGC	0.0000	Tcf21	NM_011545	Mm.16497	16	0
►	ATTAAACCAG	0.0000		AK139812	Mm.372896	16	0
	TGTGCCCCAC	0.0000	Peg3	data not found	Mm.7952	16	0
	CGGCCCTACT	0.0000	data not found	BG099818	Mm.300002	15	0
	ATGTAAAAAA	0.0000	data not found	data not found	Mm.260009, Mm.361978	15	0
	CTGTAGCAGC	0.0000	data not found	data not found		15	0
	ACTCTGGCCG	0.0001	Flna	BB670893	Mm.295533	14	0
	GGGCGTAGGT	0.0001	Hspa8	NM_031165	Mm.290774	14	0
	ACAACTGTAG	0.0001	Ivns1abp	data not found	Mm.33764	14	0
	ATTTGACCCT	0.0001	Scamp5	NM 020270	102278	14	0
	CCCTTGCCTC	0.0001	data not found	data not found	Mm.123101, Mm.222496	14	0
	AGTGTGTGCC	0.0001	data not found	data not found	Mm.20943, Mm.242644	13	0
	AATGCTAGAG	0.0003	Vbp1	NM_011692	Mm.8294	12	0
	ATTTAACTGG	0.0003	Obrgrp	BC004744	Mm.4756	12	0
	AAGCATCTGC	0.0003	Lum	NM_008524	Mm.18888	12	0
	AGAAGCTGGA	0.0003	Stxbp2	NM_011503	Mm.7247	12	0
	GAGATGATTT	0.0003	Cri1	NM_025613	Mm.44244	12	0
	GGCGTCATTG	0.0003	Siat9	NM_011375	Mm.38248	12	0
	CTATAGCCAA	0.0003	Dtx2	data not found	Mm.275574	12	0
	TTTCTTCTGG	0.0003	data not found	data not found	Mm.4419, Mm.323357	12	0
	CTTCTGCACA	0.0003	data not found	data not found	Mm.290953, Mm.378795	12	0
►	ATCGAGGTGC	0.0005	Oas1h	NM_145228	Mm.115351	11	0
	ATGACATTCC	0.0005	data not found	NM_023191	Mm.28437	11	0
	CACCTCAGGC	0.0005	Mfap2	NM_008546	Mm.7386	11	0
►	CCGCAGCTCT	0.0005	Padi6	NM_153106	Mm.271661	11	0
	TATTAAAGAA	0.0005	Hdac2	NM_008229	Mm.19806	11	0
	ACAAAGGTGG	0.0005	Rraga	AK004955	Mm.31178	11	0
	AGCTTGCCAA	0.0005	data not found	AK002397	Mm.272687	11	0
	TTTAAACAAT	0.0005	Ogt	AK075653	Mm.259191	11	0
	TCATTACGGG	0.0005	data not found	data not found	Mm.220328, Mm.4480	11	0
	TGATATTAAG	0.0005	Prdx1	data not found	Mm.30929	11	0

	SAGE Tag	ρ ≤	Common	Genbank	Unigene	Normal Tags	Null Tags
	ATGAACCACC	0.0005	data not found	data not found	Mm.32012, Mm.71498	11	0
	CACACAATGG	0.0005	data not found	data not found	Mm.307022, Mm.234965	11	0
	TCCCCACCAC	0.0005	data not found	data not found	Mm.252244, Mm.3815	11	0
	TGTAACCAAG	0.0000	Zp2	NM_011775	Mm.6510	10	0
►	CCCAGGCCCC	0.0000		BG074389	Mm.300962	10	0
	TGCATCCCCA	0.0000	Akr1a4	NM_021473	Mm.30085	10	0
	ATCAAAGCCC	0.0000	data not found	data not found	Mm.250030, Mm.378999	10	0
	TAATAGAGAA	0.0000	data not found	data not found	Mm.29870, Mm.200373	10	0
	ATATACATTT	0.0000	data not found	data not found	Mm.27090, Mm.29773	10	0
	CTATTCTTTG	0.0000	Tde1	data not found	Mm.218473	10	0
	TCGGACGGA	0.0000	data not found	data not found		10	0
	ACTAATTGAG	0.0000	multiple annotations			10	0
►	Genes highlighted in paper						
