# Supplementary material for: Ovarian gene expression in the absence of FIGLA, an oocyte-specific transcription factor
Source: BMC Dev Biol. 2007 Jun 13;7:67. doi: 10.1186/1471-213X-7-67 (PMC1906760; doi:10.1186/1471-213X-7-67)
Supplement: Additional file 8 — SAGE libraries: genes potentially down-regulated by FIGLA [file 1471-213X-7-67-S8.pdf]

Additional file 8– SAGE libraries: genes potentially down-regulated by FIGLA

	SAGE Tag	ρ ≤	Common	Genbank	Unigene	Normal Tags	Null Tags
	GAGTACTGAG	0.0000	data not found	data not found		0	149
	TATCAGAGTG	0.0000	data not found	data not found		0	111
	ACCAATGCTT	0.0000	Tuba3	NM_009446	Mm.287784	0	95
	TACGAAAGGG	0.0000	Fabp9	NM_011598	Mm.26654	0	94
	AGCCAGGAGC	0.0000	Gsg1	NM_010352	Mm.272306	0	75
	TTGAAGAAAG	0.0000	4922502D21Rik	NM_199034	Mm.46114	0	74
	CAAAATGAGA	0.0000	Fut8		Mm.35628	0	63
	TCTCTTGGTC	0.0000	Dnajb3	NM_008299	Mm.3075	0	57
	GAACCGTATA	0.0000	data not found	data not found		0	51
	ACAGAAACAA	0.0000	Sdpr	data not found	Mm.255909	0	47
	TTTAGAAAAT	0.0000	I1C0003E20	data not found	Mm.379869	0	47
	CTCTTGGTCT	0.0000	Mxd3	data not found	Mm.20350	0	45
	TACTGGGGCT	0.0000	Jarid1c	data not found	Mm.142655	0	44
■	CTACCCTCTG	0.0000	Crisp2	NM_009420	Mm.1296	0	40
	GAACCGGAAC	0.0000	Cypt4	NM_173412	Mm.46158	0	39
■	AATTATAAAT	0.0000	Cetn1	data not found	Mm.195831	0	38
	AGTGTCTTTA	0.0000	AL024210	data not found	Mm.291504	0	36
	CTTGCTATAC	0.0000	Gabra2	data not found	Mm.5304	0	35
	CTGTAAACAG	0.0000	data not found	data not found		0	35
	ACCCAACTGC	0.0000	4930571K11	data not found	Mm.229259	0	34
	GGGATGTGGC	0.0000	LOC435793	data not found	Mm.373689	0	34
	CCTAGGTGAG	0.0000	data not found	data not found		0	34
■	AATACCAGAA	0.0000	Adam3	NM_009619	Mm.5168	0	34
	AGCAATAAAG	0.0000	4930412F15Rik	NM_175517	Mm.383283	0	33
	CTAGTGAGAG	0.0000	data not found	data not found		0	33
	CTTCTCGCGC	0.0000	data not found	data not found		0	33
	AACAATTCAT	0.0000	1700074P13Rik	data not found	Mm.333010	0	33
	CTAGGTCTCC	0.0000	data not found	data not found		0	32
	TTGCCGGCAA	0.0000	Ubiquitin B	data not found	Mm.371592	0	32
	AATACATCTA	0.0000	Sp8	data not found	Mm.51682	0	32
	AAAAACTCAT	0.0000	1700027M21	NM_025499	Mm.358694	0	31
	AGAAAAATGC	0.0000	Fhl4	data not found	Mm.46163	0	31
	CACCCAAAGG	0.0000	Osp94	data not found	Mm.39330	0	31
	CCCAACTGCA	0.0000	Sorcs3	data not found	Mm.70980	0	31
	AGTGAACCTC	0.0000	Kcnk4	data not found	Mm.12894	0	30
	CGGAATAGTA	0.0000	1700010M22	data not found	Mm.45377	0	30
	GTGAACCTCC	0.0000	data not found	data not found		0	30
	AAAACTCATA	0.0000	data not found	data not found		0	29
	CCTTACTTAG	0.0000	data not found	data not found		0	29
	CAAGGCCAAG	0.0000	1700061J05	data not found	Mm.73222	0	29
■	CCGAAGGAGG	0.0000	Prm2	data not found	Mm.325769	0	29
	TGAGACAGGG	0.0000	5730596K20	data not found	Mm.264016	0	28
	GTACATAGAA	0.0000	data not found	data not found		0	28
	AGTGTGTCTC	0.0000	4933414G08	data not found	Mm.247201	0	28
	AATTGTCATG	0.0000	data not found	data not found		0	27
	CAAAGTTACC	0.0000	1700023A16	data not found	Mm.252733	0	27
	CATTATGAAA	0.0000	data not found	data not found		0	26
	CTGTAATAGT	0.0000	data not found	data not found		0	26
	CAGGACGCGC	0.0000	Tmsb10	data not found	Mm.3532	0	26
	GAGAAGCAGA	0.0000	data not found	data not found		0	25

	SAGE Tag	ρ ≤	Common	Genbank	Unigene	Normal Tags	Null Tags
	AACTAATCAG	0.0000	Tpp2	data not found	Mm.234139	0	25
	CCATGCCTTG	0.0000	Cyp17a1	NM_007809	Mm.1262	0	25
	TTTCACCTCC	0.0000	Txndc2	NM_153519	Mm.255732	0	25
	GTGCAGGGTA	0.0000	data not found	data not found		0	24
	CCCTTCTCTC	0.0000	Asrgl1	NM_025610	Mm.272847	0	24
	GTGGAAGGGC	0.0000	Gapdhs	NM_008085	Mm.374789	0	24
	CAACCTATTC	0.0000	1110018J18	data not found	Mm.227240	0	24
	AGCATCCCAC	0.0000	4930455B06	XM_127913	Mm.159281	0	24
	CCCGCATCTT	0.0000	Irgc1	NM_199013	Mm.33811	0	24
	AAAACCCTGT	0.0000	1700051I15	data not found	Mm.271161	0	24
■	ATGAAGATGC	0.0000	Dkkl1	NM_015789	Mm.27287	0	24
	TCTTCTCGCG	0.0000	LOC545867	data not found	Mm.380076	0	24
	CAGGGGGATG	0.0000	Tcp10b	data not found	Mm.264651	0	24
■	TAAGAAACAT	0.0000	Cypt12	XM_619877	Mm.27023	0	23
	GTCATCACAT	0.0000	data not found	data not found		0	23
	GCCAGGAGCT	0.0000	data not found	data not found		0	23
	GACACCCAAG	0.0000	data not found	data not found		0	23
	CCTCCTTATT	0.0000	data not found	data not found		0	22
	GCATATGGAT	0.0000	data not found	data not found		0	22
	TCTGCTCACT	0.0000	Cklfsf2a	NM_027022	Mm.272746	0	22
	AAAGACCCCC	0.0000	1500006O09	data not found	Mm.379105	0	22
	GAACTATGGG	0.0000	LOC546598	data not found	Mm.326801	0	22
■	ACGGCAGTCC	0.0000	Ldhc	data not found	Mm.16563	0	22
	TTGGGGGAGG	0.0000	Dusp18	data not found	Mm.32588	0	22
	GTGTTGAAAG	0.0000	data not found	data not found		0	22
	CCACCCGATA	0.0000	1700123L14	NM_030107	Mm.84977	0	22
	CACTCTGAAA	0.0000	Ldhal6b	NM_175349	Mm.358813	0	21
	CCACTGTCAC	0.0000	Slc2a3	data not found	Mm.269857	0	21
	TGTGATGGTG	0.0000	Klk6	NM_010639	Mm.142722	0	21
	TTAGAAGAAG	0.0000	Cdca7l	data not found	Mm.281149	0	21
	ACCTCCTTTG	0.0000	1700016P04	data not found	Mm.177847	0	20
	CATCAGGATT	0.0000	Senp7	data not found	Mm.255784	0	20
	ACTTGCTATA	0.0000	Prdx6-rs1	data not found	Mm.60992	0	20
	CCTCTCCCAG	0.0000	Glul	data not found	Mm.210745	0	20
	AAAACATCAC	0.0000	Cdc2l6	data not found	Mm.200924	0	20
	TTGGACAATC	0.0000	TISP22	data not found	Mm.326636	0	20
	CTAGACAAGA	0.0000	Stx6	data not found	Mm.66264	0	20
	CTCCCTTCCC	0.0000	D830044I16	data not found	Mm.304201	0	20
	CTCATGGGTG	0.0000	Cklfsf2b	NM_028524	Mm.232593	0	20
	CTTCTAATTA	0.0000	2410015A16	NM_029627	Mm.273319	0	19
	TGCAGAAGAA	0.0000	Lmo7	data not found	Mm.218981	0	19
	CAAGCTTCAG	0.0000	Spata19	NM_029299	Mm.45824	0	19
	GTGTACAAGG	0.0000	4933402P03	NM_175368	Mm.73263	0	19
	CCTTAATACA	0.0000	4930572D21	XM_355845	Mm.160043	0	19
	AACAACCCAA	0.0000	Sf3a1	data not found	Mm.156914	0	19
	GTATTGTTCT	0.0000		data not found		0	19
	AAAAAGCCAA	0.0000	Nexn	data not found	Mm.200188	0	19
	CAGCGCAGCT	0.0000	1700095G12	XM_126335	Mm.159159	0	18
	ACCGTGCTGT	0.0000	5730436H21	data not found	Mm.29627	0	18
■	GGTAATTGCG	0.0000	Tnp1	data not found	Mm.661	0	18
■	ACAGCCATCC	0.0000	Tisp78	NM_144827	Mm.156123	0	18
	TCGTAGCTGC	0.0000	Gpd2 TISP38	NM_010274	Mm.3711	0	18

	SAGE Tag	ρ ≤	Common	Genbank	Unigene	Normal Tags	Null Tags
	ACTGGGGCTG	0.0000	Ppil1	data not found	Mm.328928	0	18
	GCCTCACAGG	0.0000	D10Wsu52e	data not found	Mm.9257	0	18
	CCCTTCCCTG	0.0000		data not found		0	18
	CACGAGGAGG	0.0000	Fscn3	NM_019569	Mm.31708	0	18
	CTGCAGCCAT	0.0000	Hspb9	data not found	Mm.46175	0	18
	TGTGAAAAGG	0.0000	1700019M22	NM_027076	Mm.46121	0	18
	ACTAGACAAG	0.0000	Mcsp	data not found	Mm.331192	0	17
	ATCCAATGGG	0.0000	data not found	data not found		0	17
	GTGTACCGTC	0.0000	data not found	data not found		0	17
	GAACCCATTG	0.0000	1700012L04	data not found	Mm.327561	0	17
	GGGCATCTAG	0.0000	Clgn	NM_009904	Mm.358581	0	17
	ATGGCCCAGA	0.0000	Sdh1	NM_146126	Mm.371580	0	17
	GATTCAATAA	0.0000	data not found	data not found		0	17
	GTGTAAATGG	0.0000	data not found	data not found		0	17
	CCGAGTAGAG	0.0000	Meig1	data not found	Mm.2688	0	17
	GCACCTAAGA	0.0000	Pgam2	NM_018870	Mm.219627	0	17
	CAATAAATGC	0.0000	data not found	data not found		0	17
	AATATTGAAG	0.0000	4930511I11	NM_026290	Mm.46170	0	17
	CTCACCATGT	0.0000	Rai14	data not found	Mm.212395	0	17
	CTAAACTGAG	0.0000	data not found	data not found		0	17
	AAAGAATCAC	0.0000	glycogen phosphorylase		Mm.183821	0	17
	CTCACCTCTG	0.0000	Ropn1l	NM_145852	Mm.309193	0	16
	AACGAAGAAC	0.0000	Speer1-ps1	XM_620159	Mm.335888	0	16
	CAGACAGTGG	0.0000	A630018P17	data not found	Mm.336318	0	16
	AAATGGACAC	0.0000	Tnp2	data not found	Mm.206798	0	16
	CAAGAGAGCA	0.0000	4930583C14	NM_029472	Mm.268996	0	16
	CTCACAGGCC	0.0000	Ctsd	data not found	Mm.231395	0	16
	ATACCAGAAG	0.0000	data not found	data not found		0	16
	CTGCTGCAGA	0.0000	data not found	data not found		0	16
	CAAGAGGCCC	0.0000	CPN60	data not found	Mm.1777	0	16
	TTGATGGAAC	0.0000	Gkrs2	NM_010294	Mm.61206	0	16
	GAAGGAAGAG	0.0000	Ppp1r3a	data not found	Mm.209429	0	16
	TTGGATACAG	0.0000	Kif2b	XM_126653	Mm.67677	0	16
	AACATGGAAG	0.0000	Csnb	data not found	Mm.268737	0	16
	AAGAAACATC	0.0000	Bub3	data not found	Mm.927	0	16
	CTATTCCTTT	0.0000	Papolb	NM_019943	Mm.358676	0	16
	CATGTGCCCC	0.0000	Pdcd6ip	data not found	Mm.29816	0	16
	GGGACCACGA	0.0000	data not found	data not found		0	15
	AACGCAAGCT	0.0000	data not found	data not found		0	15
■	CTGAGAGGGG	0.0000	Dnahc8	NM_013811	Mm.254797	0	15
	TTCCAGGAGA	0.0000	Ubxd3	NM_178671	Mm.292226	0	15
■	TTATAAGGTG	0.0000	Odf1	NM_008757	Mm.252830	0	15
	AGCTCTCCTC	0.0000	Morc2b	NM_177719	Mm.157765	0	15
	AAACCCTGTC	0.0000	I830058E16	data not found	Mm.310642	0	15
	CTTCCCAGGG	0.0000	Rps25	data not found	Mm.297486	0	15
	CATCTCAAAG	0.0000	1700001F22	NM_027036	Mm.331893	0	15
■	AAGTGACTGG	0.0000	Tcfl5	NM_178254	Mm.23379	0	15
■	ATCCTGCCCT	0.0000	Oaz3 TISP15	NM_016901	Mm.331200	0	15
	ACTTCCCAGG	0.0000	Mkrn1	data not found	Mm.270484	0	15
	GACTGAGGGT	0.0000	1700093K21	NM_026105	Mm.87328	0	15
	ACTCACAGGC	0.0000	D11Wsu68e	data not found	Mm.301020	0	15
	TGTTTGATAT	0.0001	Mkl1 MKL	NM_153049	Mm.24862	0	14

	SAGE Tag	ρ ≤	Common	Genbank	Unigene	Normal Tags	Null Tags
	CACGAACTGG	0.0001	data not found	data not found		0	14
	AATACTCTGC	0.0001	data not found	data not found		0	14
	GCAGGAACCC	0.0001	data not found	data not found		0	14
	GTTGCCTGAT	0.0001	data not found	data not found		0	14
	AAACTATAAA	0.0001	4933400N17	data not found	Mm.380540	0	14
	AAAATCGCAG	0.0001	Sqrdl	NM_021507	Mm.28986	0	14
	CGGGCTTCTT	0.0001	Cyp11a1	NM_019779	Mm.302865	0	14
	CTGAAAGACC	0.0001	Rnaseh2a	data not found	Mm.182470	0	14
	CCCCCAATTG	0.0001	Ctsb	data not found	Mm.236553	0	14
	CAAATCAGAA	0.0001	B4galt1	data not found	Mm.15622	0	14
	CTGCTCACAG	0.0001	Stk33	XM_489715	Mm.253614	0	14
	AACTGAAGAA	0.0001	Rnf139	data not found	Mm.4537	0	14
	TAAGCACAAT	0.0001	Herc4	data not found	Mm.234437	0	14
	AATGTCGAGG	0.0001	Gkrs1	NM_010293	Mm.32242	0	14
	TAAGGTTCCC	0.0001	data not found	data not found		0	14
	AGGGCACTGT	0.0001	4930449C09	data not found	Mm.148858	0	14
	AACTCAAAGG	0.0001	2510027N19	data not found	Mm.4467	0	14
	CTGCATTCTT	0.0001	LOC545951	XM_622405	Mm.212736	0	13
	GTGTGTCTCC	0.0001	1300007B12	data not found	Mm.379087	0	13
	CTAATATTTT	0.0001	D330027G24	data not found	Mm.101339	0	13
	TGTGTGCTGT	0.0001	Akr1c19	data not found	Mm.22832	0	13
	GATGCCATCA	0.0001	Dnmt3b6	data not found	Mm.89772	0	13
	CCCTTTTGCC	0.0001	Fbxo36	NM_025386	Mm.27888	0	13
	GACCACAGCC	0.0001	Slc4a1	data not found	Mm.7248	0	13
	TTTGCAACTC	0.0001	data not found	data not found		0	13
	AAATTGAGAG	0.0001	1810015C04	data not found	Mm.25311	0	13
	TAAGAGAAAG	0.0001		data not found		0	13
	AAACCAGGTG	0.0001	4921533L14	data not found	Mm.87130	0	13
	AAATCTCACT	0.0001	0610010D20	data not found	Mm.24196	0	13
	AAAGGATTGC	0.0001	data not found	data not found		0	13
	TGTGGAGGAA	0.0001	AV340375	NM_001001295	Mm.268341	0	13
	CCTGTGAAAA	0.0001	Lancl3	data not found	Mm.245119	0	13
	TTTACAGTGT	0.0001	data not found	data not found		0	13
	AAAGAGAAAA	0.0001	Pbx3	data not found	Mm.239941	0	13
	TCCTTGTCAA	0.0001	data not found	data not found		0	13
	GCATCCCACT	0.0001	data not found	data not found		0	13
	GCTCTGTGGT	0.0001	data not found	data not found		0	13
	CTGTCACATC	0.0001	data not found	data not found		0	13
	CCACCCCAGT	0.0001	Ephx2	NM_007940	Mm.15295	0	13
	TCCAGACCGT	0.0001	Dnajb1	data not found	Mm.282092	0	13
	TGCGGGTAGT	0.0001	Bnipl	NM_001024841	Mm.331209	0	13
	CTGCTCACTG	0.0002	Plxnb3	data not found	Mm.380177	0	12
	CAGAAACAAT	0.0002	BC029103	XM_621455	Mm.34723	0	12
	TTTGAGCATG	0.0002	data not found	data not found		0	12
■	GGCTGCAGAC	0.0002	Tex101	NM_019981	Mm.23385	0	12
	ATAAAGGGAA	0.0002	Tnfsf5	data not found	Mm.4861	0	12
	GAAAAATGCT	0.0002	data not found	data not found		0	12
	CTTGTGTTGA	0.0002	data not found	data not found		0	12
	CGCACTCGAA	0.0002	data not found	data not found		0	12
	ATCGAGTGTA	0.0002	Ube2n	data not found	Mm.371667	0	12
	GCAGAGATGT	0.0002	4933409I22	NM_172914	Mm.248902	0	12
	TTCAACAAGT	0.0002	Rsn	NM_019765	Mm.241109	0	12

	SAGE Tag	ρ ≤	Common	Genbank	Unigene	Normal Tags	Null Tags
	TTGTACTTTT	0.0002	Dncl2b	NM_029297	Mm.23114	0	12
	CAGCGCATTG	0.0002	1700006D24	NM_027026	Mm.250465	0	12
	TGATGTGTTT	0.0002	1700008K24	data not found	Mm.46140	0	12
	GTGCTATCAG	0.0002	1700126L10	data not found	Mm.374829	0	12
	CAAAACACAC	0.0002	4932419B04	data not found	Mm.292354	0	12
	GATACCGTCC	0.0002	data not found	data not found		0	12
	AAAAACATGA	0.0002	E430034L04	data not found	Mm.290530	0	12
	GATACTCTGC	0.0002	Tns	XM_619639	Mm.309975	0	12
	GCTCTCCTCC	0.0002	Hba-x	data not found	Mm.141758	0	12
	CCTTCTCTCT	0.0002	D130072O21	data not found	Mm.88364	0	12
	GATGTGTCCT	0.0002	1700010I14	NM_025851	Mm.67591	0	12
	AAAAATAAAT	0.0002	2300009A05	data not found	Mm.113942	0	12
	CACATCCGGT	0.0002	Adn	data not found	Mm.4407	0	12
	GGTTGAGGGG	0.0002	data not found	data not found		0	12
	GACATATGGA	0.0002	Dyrk3	NM_145508	Mm.39299	0	12
	TGTTGAAGAG	0.0002	4930431J08	data not found	Mm.86986	0	12
	GAAGCGTAAG	0.0002	data not found	data not found		0	12
	ACTGCTTGGG	0.0002	Mgmt MGMT	data not found	Mm.71906	0	12
	AGGTCCTCGT	0.0002	Rnf133	NM_198251	Mm.379572	0	12
	AAGGTTCCCC	0.0002	data not found	data not found		0	12
	CCCGCCATGG	0.0002	1700029H14	NM_025601	Mm.46136	0	12
	GGTCCTCGTG	0.0002	data not found	data not found		0	12
	CTGCTGCTAT	0.0002	Miz1	data not found	Mm.6370	0	12
	AAATCAACTG	0.0002	4932408B21	data not found	Mm.61131	0	12
	GGTAGAAATG	0.0002	Sas	data not found	Mm.35650	0	12
	TGGTACGGGT	0.0002	1700009N14	XM_131323	Mm.23522	0	12
	TTGTAAACCC	0.0002	data not found	data not found		0	12
	GGGGAATGGT	0.0002	data not found	data not found		0	12
	CACTGTCACC	0.0002	B230382K22	data not found	Mm.41219	0	12
	TCCATCCCTT	0.0002	Sprr1a	data not found	Mm.331191	0	12
	AGACCTGTCT	0.0002	GAPDH	data not found	Mm.370388	0	12
■	CACAAGAAAT	0.0004	Ppp3r2	data not found	Mm.46125	0	11
	CAGGCCTTGT	0.0004	Iqcf3	data not found	Mm.141451	0	11
	GTATGTATTT	0.0004	Fbxo41	data not found	Mm.38777	0	11
	GTATTTCAAT	0.0004	data not found	data not found		0	11
	CCAACGAGAA	0.0004	data not found	data not found		0	11
	GTCCCGGAAG	0.0004	data not found	data not found		0	11
■	GCCGAGCTGC	0.0004	Sstk	NM_032004	Mm.379270	0	11
	GGAGAATAAC	0.0004	Etf1	data not found	Mm.329353	0	11
	CCAACAATGT	0.0004	Tubb5	data not found	Mm.273538	0	11
	GGGCATTAGA	0.0004	4933407P14	data not found	Mm.117063	0	11
■	GTCAGCAACC	0.0004	Akap3	NM_009650	Mm.87748	0	11
	TCCCCTTGCC	0.0004	6430706D22	data not found	Mm.386915	0	11
■	AGTATGTATT	0.0004	Crisp2	data not found	Mm.1296	0	11
	GACCCCCTTG	0.0004	data not found	data not found		0	11
■	CGGTTGGTGA	0.0004	SP18	XM_149824	Mm.33683	0	11
	CAGGAGATAA	0.0004	Cdv3 Pp36	XM_135479	Mm.261025	0	11
	TCTGCATTCT	0.0004	data not found	data not found		0	11
	CTGTCACTTC	0.0004	Elavl2	data not found	Mm.318042	0	11
	TGACAAATGA	0.0004	Pvrl3	NM_021495	Mm.328072	0	11
	TCTTAAGCAA	0.0004	1700019B01	data not found	Mm.307198	0	11
	TAAAGAATCA	0.0004	Dpysl3	data not found	Mm.8180	0	11

	SAGE Tag	ρ ≤	Common	Genbank	Unigene	Normal Tags	Null Tags
	AGTGAGAGAG	0.0004	9630025C22	data not found	Mm.54257	0	11
	CCGGGGACAC	0.0004	Acsl1	NM_007981	Mm.210323	0	11
	GTTAGACCAC	0.0004	data not found	data not found		0	11
	ACCCAAGCCA	0.0004	Armc3	XM_622876	Mm.276696	0	11
■	AACCACAATG	0.0004	Tekt1	NM_011569	Mm.42257	0	11
	GAAGGACACT	0.0004	data not found	data not found		0	11
	GATACTTACT	0.0004	data not found	data not found		0	11
	AAACCAACCC	0.0004	Hoxb9	data not found	Mm.258271	0	11
■	ATGCATGTGA	0.0004	Hils1 TISP64	NM_018792	Mm.30482	0	11
	GTTGAGAATT	0.0004	data not found	data not found		0	11
	GAAATGCGTC	0.0004	Tdrd6	NM_198418	Mm.329058	0	11
	CGACACCAAG	0.0004	9130403J09	data not found	Mm.42767	0	11
	GGGTTTACCT	0.0009	data not found	data not found		0	10
	TCTCTTCAGA	0.0009	Mjd	data not found	Mm.271914	0	10
	AGAAGGTTCA	0.0009	Iqwd1	data not found	Mm.227605	0	10
	GTGCCTGGCG	0.0009	Siat5	data not found	Mm.200388	0	10
	AATGGACACA	0.0009	Mass1	data not found	Mm.288694	0	10
	GGGCAGAGAT	0.0009	data not found	data not found		0	10
■	TGGTCCCTAG	0.0009	Ppp3r2	data not found	Mm.46125	0	10
	GGCCCTAAAG	0.0009	data not found	data not found		0	10
	GCTCCAACCC	0.0009	data not found	data not found		0	10
	CATAGTGTCT	0.0009	1700013F07	XM_131080	Mm.271255	0	10
	ACCACTGAAT	0.0009	XP_148894	XM_148894	Mm.301487	0	10
	TGATGACTCA	0.0009	data not found	data not found		0	10
	TGGACAATCC	0.0009	data not found	data not found		0	10
	TGAAGATGCC	0.0009	data not found	data not found		0	10
	CCTGTAAATG	0.0009	2610205H19	data not found	Mm.195625	0	10
	CCTGCCCTCC	0.0009	2510039O18	data not found	Mm.183076	0	10
	CCTGCCTCAG	0.0009	Sox17	data not found	Mm.279103	0	10
	CTTTAGATAG	0.0009	data not found	data not found		0	10
	GGCTCTTAAA	0.0009	data not found	data not found		0	10
	CGTCTGGGTC	0.0009	Actl7a	NM_009611	Mm.30959	0	10
	CATGCCTTGA	0.0009	data not found	data not found		0	10
	GGGAAGCAGA	0.0009	Sec14l4	data not found	Mm.337476	0	10
	CTTCATGAGG	0.0009	1700034O15	NM_029671	Mm.272520	0	10
	TCAACATGAG	0.0009	Ndufb4	data not found	Mm.29065	0	10
	GTCTGTGTGC	0.0009	data not found	data not found		0	10
	CAACAGAAGT	0.0009	1700052H20	data not found	Mm.307436	0	10
	TAAGATAATC	0.0009	Enpp3	data not found	Mm.338425	0	10
	CACCCGATAT	0.0009	data not found	data not found		0	10
	ATCGGAAGGA	0.0009	Hsbp1	data not found	Mm.358714	0	10
	GTGATGGTAT	0.0009	data not found	data not found		0	10
	CTCTCCCACC	0.0009	Jmy	data not found	Mm.100273	0	10
	TTCAACATGA	0.0009	Kcne3	NM_020574	Mm.282386	0	10
	TCAAAGCCAA	0.0009	BC049635	NM_177785	Mm.212727	0	10
	GTATTGATTT	0.0009	data not found	data not found		0	10
	TTAAGATAAT	0.0009	Sdh1	data not found	Mm.371580	0	10
	TCAACCTCTT	0.0009	Rnf151	data not found	Mm.46159	0	10
	CAATAAATGT	0.0009	2410002F23	data not found	Mm.274492	0	10
	GCAGCACAGC	0.0009	1700008D07	XM_620533	Mm.214841	0	10
	TTTGAATAAC	0.0009	data not found	data not found		0	10
	TCCTTGGGCA	0.0009	data not found	data not found		0	10

	SAGE Tag	ρ ≤	Common	Genbank	Unigene	Normal Tags	Null Tags
	GTAAATTGGC	0.0009	data not found	data not found		0	10
■	Testis-specific genes						
