# Supplementary material for: Ovarian gene expression in the absence of FIGLA, an oocyte-specific transcription factor
Source: BMC Dev Biol. 2007 Jun 13;7:67. doi: 10.1186/1471-213X-7-67 (PMC1906760; doi:10.1186/1471-213X-7-67)
Supplement: Additional file 9 — Primers used for genotyping Figla null mice (5'-3') [file 1471-213X-7-67-S9.pdf]

Additional file 9 - Primers used for genotyping *Figla* null mice (5`- 3`)

Figla Forward	TGGCC CCTTCGTGGTACCACC
Figla Reverse - Normal	GGGCCTGTGAAGGGTCAGCCC
Figla Reverse - Null	CATTTGTCACGTCCTGCACGAC

Conditions: 95°C 5 min, 94°C 30 sec, 60°C 30 sec, 72°C 30 sec for 28 cycles; 72°C 7 min
